# Spatially resolved C1QC^+^ macrophage-CD4^+^ T cell niche in colorectal cancer microenvironment: implications for immunotherapy response

**DOI:** 10.1038/s41421-025-00811-2

**Published:** 2025-07-01

**Authors:** Hangyu Zhang, Libing Hong, Zhen Dong, Shan Xin, Bo Lin, Jinlin Cheng, Weihong Tian, Bin Li, Jing Wang, Xiaoyan Liu, Chuan Liu, Yuzhi Jin, Yanzhi Feng, Ge Su, Xuqi Sun, Qiqi Liu, Xiaomeng Dai, Yang Gao, Zhou Tong, Lulu Liu, Xudong Zhu, Yi Zheng, Peng Zhao, Tiannan Guo, Weijia Fang, Xuanwen Bao

**Affiliations:** 1 Department of Medical Oncology, The First Affiliated Hospital, School of Medicine, Zhejiang University, Hangzhou, Zhejiang China; 2 Westlake Center for Intelligent Proteomics, Westlake Laboratory of Life Sciences and Biomedicine, Hangzhou, Zhejiang China; 3 School of Medicine, School of Life Sciences, Westlake University, Hangzhou, Zhejiang China; 4 Research Center for Industries of the Future, Westlake University, Hangzhou, Zhejiang China; 5 Department of Genetics, Yale School of Medicine, New Haven, USA; 6 Innovation Centre for Information, Binjiang Institute of Zhejiang University, Hangzhou, Zhejiang China; 7 School of Software Technology, Zhejiang University, Ningbo, Zhejiang China; 8 State Key Laboratory for Diagnosis and Treatment of Infectious Diseases, The First Affiliated Hospital, School of Medicine, Zhejiang University, Hangzhou, Zhejiang China; 9 Changzhou Third People’s Hospital, Changzhou Medical Center, Nanjing Medical University, Changzhou, Jiangsu China; 10 Department of Pathology, The First Affiliated Hospital, School of Medicine, Zhejiang University, Hangzhou, Zhejiang China; 11 Department of lmmunobiology, Yale School of Medicine, New Haven, USA; 12 College of Computer Science, Zhejiang University, Hangzhou, Zhejiang China; 13 Department of Radiation Oncology, First Affiliated Hospital, School of Medicine, Zhejiang University, Hangzhou, Zhejiang China; 14 National Key Laboratory of Advanced Drug Delivery and Release Systems, Zhejiang University, Hangzhou, China; 15 Key Laboratory of Cancer Prevention and Intervention, Ministry of Education, Hangzhou, China

**Keywords:** Cancer microenvironment, Colorectal cancer

## Abstract

Colorectal cancer (CRC), including both microsatellite instability (MSI) and microsatellite stability (MSS) subtypes, frequently exhibits intrinsic resistance to immunotherapy. However, the spatial tumor microenvironment (TME) and its role in distinguishing immunotherapy responders from non-responders remain poorly understood. In this study, spatial multiomics, including imaging mass cytometry (*n* = 50 in-house), spatial proteomics (*n* = 50 in-house), and spatial transcriptomics (*n* = 9 in-house), were employed to elucidate the spatial TME of metastatic CRC (mCRC) patients receiving immunotherapy. These methodologies were integrated with single-cell RNA sequencing (scRNA-seq), bulk RNA-seq, and bulk proteomics for comprehensive analysis and validation. A spatial immune atlas containing 314,774 cells was constructed. We found that C1QC^+^ resident tissue macrophages (RTMs) were more abundant in responders regardless of microsatellite status. Co-localization of C1QC^+^ RTMs with CD4^+^ T cells was observed in responders, and MHC-II expression facilitated their interaction. In contrast, cancer-associated fibroblasts inhibited this interaction in non-responders. Moreover, whole genome screening identified key genes involved in antigen presentation in C1QC^+^ RTMs. Hence, our study highlights the importance of spatial immune mapping in revealing the complex spatial topology of CRC and corresponding immunotherapy response.

## Introduction

Colorectal cancer (CRC) is the third most common cancer, the second leading cause of cancer-related death, and a major public health issue^1^. Despite clinical advances in recent years, the 5-year survival rate for patients with metastatic CRC (mCRC) remains low, at approximately 14%^2^. New treatment strategies for mCRC, especially immunotherapy, have radically changed the cancer treatment paradigm^3^. There are remarkable dichotomy of tumor immune phenotypes in CRC: microsatellite stability (MSS) and microsatellite instability (MSI). Among them, MSS mCRC (accounting for 95% of all mCRC cases) presents primary resistance to immune checkpoint inhibitors (ICIs)^4^. MSI mCRC is thought to be sensitive to ICIs, but the reported 43.8% objective response rate (ORR)^5^ is barely satisfactory. Multiple combination therapeutic modalities, such as ICIs combined with tyrosine kinase inhibitors (TKIs)^6,7^ or systemic chemotherapy^8^, have been used in an attempt to improve immunotherapy efficacy for CRC. However, there are no reproducible or consensus results to support their effectiveness, especially in patients with the MSS subtype; therefore, challenges remain, concerning the mechanism of immunotherapy resistance as well as potential biomarkers for CRC patients.

With the advent of single-cell RNA sequencing (scRNA-seq), emerging evidence suggests that the efficacy of ICIs largely depends on the diversity and phenotype of cells in the tumor microenvironment (TME). For instance, tumor-associated macrophages (TAMs) are relevant immunotherapy targets, as their coexpressed genes encode immunostimulatory, immunosuppressive and reparative factors^9,10^. Molecules, such as IL1B, TREM2, FOLR2 and C1Q, have emerged as more identifiable macrophage biomarkers for specific populations. Additionally, the single-cell landscape of CRC has been well elucidated. For instance, Zhang et al. reported that two strictly exclusive TAM populations, consisting of C1QC^+^ and SPP1^+^ TAMs, contribute to different functions like antigen presentation or angiogenesis regulations in CRC, and are responsible for opposite anti-tumor or pro-tumor activity^11^. Li et al. found that proinflammatory IL1B^+^ TAMs decreased after response to immunotherapy, providing potential target for improving treatment efficacy^12^. Notably, scRNA-seq studies provide detailed information on cell populations with distinct molecular features; however, they have inherent limitations in preserving spatial topology information. This loss occurs during the tissue dissociation process. Currently, spatial-omics provides new insights into the network and the spatial topology of the TME. For example, Qi et al. revealed that the infiltration of FAP^+^ fibroblasts and SPP1^+^ macrophages is highly correlated in CRC and that their presence is negatively correlated with lymphocyte infiltration^13^. However, the previous studies were conducted with treatment-naïve samples and had limited information on patient immunotherapy outcome. The impact of spatial topology on immunotherapy response in mCRC patients has yet to be investigated, particularly at a single-cell spatial resolution.

In this study, we employed an integrative approach that combines imaging mass cytometry (IMC), spatial transcriptomics, spatial proteomics, scRNA-seq, bulk proteomics and bulk RNA-seq to construct a comprehensive analysis of spatial immune atlas at the single-cell level, offering high-throughput spatial phenotypic information in mCRC patients who underwent immunotherapy. By utilizing advanced deep learning methods, we investigated the spatial topology of CRC and explored the potential mechanism of resistance to immunotherapy in CRC.

## Results

### A subset of MSS and MSI mCRC patients responded to immunotherapy

**Fig. 1:**
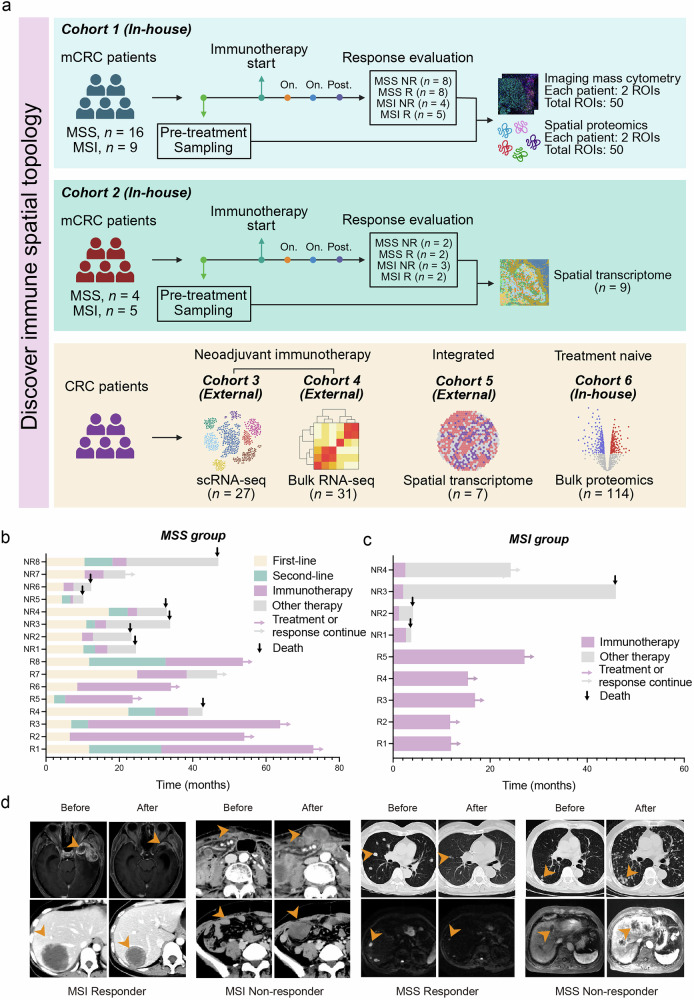
A subset of MSS and MSI mCRC patients responded to immunotherapy. **a** Overview of the study design. **b** Progression-free survival (PFS) of systemic chemotherapy and immunotherapy in 16 microsatellite stable (MSS) distant mCRC patients, including 8 responded (R) patients and 8 non-responded (NR) patients. **c** PFS of immunotherapy in 9 microsatellite instable (MSI) CRC patients, including 5 R patients and 4 NR patients. **d** Representative CT images of MSS/MSI_R and NR patients.

The research methodology is illustrated in Fig. 1a. The immune spatial topology of 25 mCRC patients who underwent immunotherapy (in-house cohort 1) was investigated via both spatial IMC analysis (regions of interest (ROIs), *n* = 50) and spatial proteomics analysis (ROIs, *n* = 50). Furthermore, spatial transcriptomics was employed to explore the spatial TME characteristics at the whole-slide level in 9 mCRC patients receiving immunotherapy (in-house cohort 2). To validate our findings, we analyzed scRNA-seq (*n* = 27) and bulk RNA-seq (*n* = 31) data from CRC patients who received neoadjuvant immunotherapy (external cohorts). Additionally, we utilized spatial transcriptomics data (*n* = 7) from the integrated external CRC cohort and proteomics data (*n* = 114) from treatment-naïve patients in the in-house cohort.

For cohort 1, all 25 mCRC patients in this study were enrolled at The First Affiliated Hospital of Zhejiang University. We screened a total of 502 mCRC patients and finally acquired 16 MSS mCRC patients from the previous study we presented at the last ASCO annual meeting^14^ and another 9 MSI mCRC patients who met all the criteria to be included in this study (Supplementary Fig. S1). The inclusion and exclusion criteria are available in Materials and Methods. Sixteen mCRC patients with the MSS subtype received ICIs combined with regorafenib as a third-line therapy according to the REGONIVO study^15^, and another nine MSI mCRC patients received ICI monotherapy as a first-line treatment according to the KEYNOTE 177 study^16^. Responders were defined as patients who maintained stable disease (SD) status or achieved partial remission for more than 6 months, whereas non-responders were those whose disease progressed within the same period. This classification aligns with the complexity of tumor response in immunotherapy. SD patients were considered responders as emerging evidence suggests that those who maintain SD status for more than 6 months without tumor growth derive real clinical benefit^17^. In general, immunotherapy-responded patients demonstrated significantly prolonged survival (Supplementary Fig. S2a, b). At the last follow-up visit in November 2023 (median follow-up of 16.89 months (IQR 11·12–26·33)), almost all patients (11/13) who responded to immunotherapy were in continuous remission or treatment (Fig. 1b, c). Among the MSS group, the median progression-free survival (PFS) was not reached vs 3.00 months (95% CI 2.53–3.47) between R and NR (*P* < 0.001) (Fig. 1b; Supplementary Fig. S2c). In the MSI group, the median PFS was also not reached vs 2.07 months (95% CI 0.77-3.37) between R and NR (*P* = 0.003) (Fig. 1c; Supplementary Fig. S2d). Superiority of overall survival (OS) was also observed in both MSS_R and MSI_R subgroup compared with NR subgroups (Supplementary Fig. S2e, f).

The clinical characteristics are shown in Supplementary Table S1. Consistent with previous report^18,19^, the liver was the most common site of metastasis (11/25), followed by the lung (9/25) and peritoneum (7/25) (Supplementary Fig. S2g). Yu et al. revealed that liver metastases can create a systemic immune desert properties and diminish immunotherapy efficacy^20^. In our cohort, immunotherapy response was observed in both patients with and without liver metastasis, and there was no significant difference in the proportion of patients with liver metastasis between R and NR patients (Supplementary Table S2). In addition, tumors arising from different primary tumor sites (PTSs) of the colon are also considered to have clinically and molecularly distinct heterogeneity^21^. There was no significant difference in the location of PTS between the R and NR cohorts in our study, with MSS CRC patients mainly having left-sided PTS, and MSI CRC patients having both the left-sided and right-sided PTS (Supplementary Fig. S2h). Imaging efficacy evaluation was conducted via repeated computed tomography (CT) scans, and the representative images of the four subgroups are shown in Fig. 1d.

In summary, for the primary cohort 1, data from 25 ICI-treated patients, divided into four subgroups (MSI_R, MSI_NR, MSS_R and MSS_NR), were collected for IMC and paired spatial proteomics analysis. Significantly prolonged PFS and prolonged OS were observed in MSS_R and MSI_R patients. There were no clinically related prognostic interference, such as the location of the PTS or the occurrence of liver metastases, between R and NR patients.

### Establishment of a spatial immune atlas of MSS/MSI_R and NR mCRC patients at single-cell resolution

**Fig. 2:**
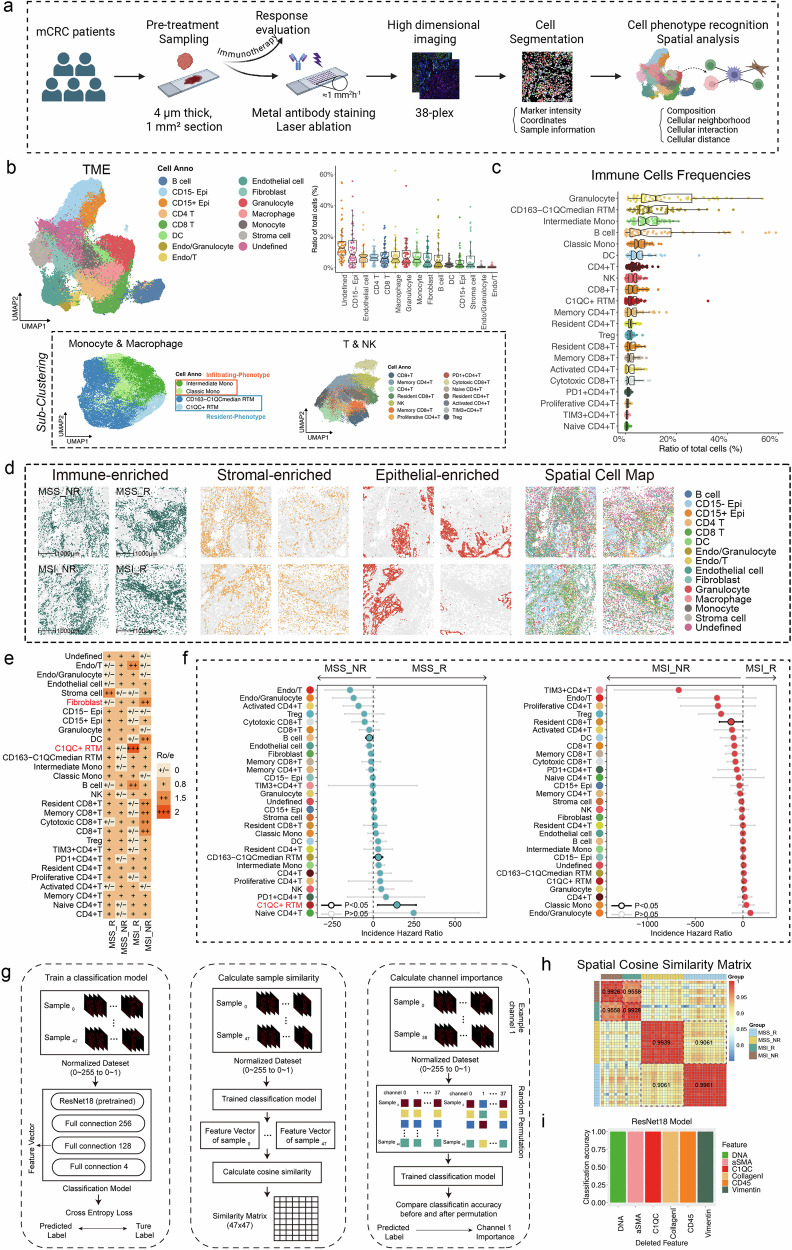
Spatial immune phenotypes of MSS/MSI_R and NR CRC patients at single-cell resolution. **a** The workflow of IMC analysis. **b** UMAP plot visualizing the clustering of broad cell types within the IMC dataset (top left). Box plot showing the relative frequencies of each major cell type among all cells in the IMC dataset, providing an overview of their abundance in the analyzed samples (top right). UMAP plots showing the clustering of monocytes and macrophages, T cells and NK cells in the IMC dataset (bottom). **c** The immune cell subsets frequencies in total cells. **d** Spatial distribution of cells (immune, stromal, and epithelial cells, and major cell subtypes) in representative sections from the four groups, annotated based on manual classification results. **e** Prevalence of immunotherapy outcomes for each cell cluster, as estimated by the cell frequency-based Ro/e analysis. **f** Risk ratios illustrating the associations between cell frequency and immunotherapy response are shown for each cell subpopulation in the MSS (left) and MSI (right) TME, as determined by logistic regression analysis. **g** Workflow of cosine similarity and ResNet18-based deep learning. **h** Heatmap visualization of cosine similarity scores between TME images. Each cell represents the pairwise similarity score between two samples, with warmer colors indicating higher similarity (scale: 0.75–1.0). The labeled values indicate the average similarity computed within individual groups or between different groups. **i** Feature importance calculated by ResNet18-based deep learning.

We first set out to investigate whether spatial TME features differ between MSS/MSI_R and NR CRC tumors. We collected formalin-fixed, paraffin-embedded (FFPE) samples from 25 mCRC patients prior to immunotherapy for IMC analysis. Treatment response was evaluated after the completion of immunotherapy to stratify patients into four subgroups (MSS_NR, *n* = 8; MSS_R, *n* = 8; MSI_NR, *n* = 4; MSI_R, *n* = 5) (Fig. 2a). Two ROIs for each patient were selected for a comprehensive overview of the spatial organization of cells within the TME (Supplementary Figs. S3, S4). First, samples were stained using a 38-plex metal-tagged antibody panel (Fig. 2a). The panel included lineage and functional immune cell markers, such as CD4 for T cells and CD163 for macrophages, surrogates of cell states like Ki-67 for proliferation, and structural markers cover epithelium, and stroma cells (Supplementary Fig. S5a and Table S3). Next, laser ablation was performed to acquire high-dimensional histopathological images (Fig. 2a), which were further improved by signal compensation, image denoising and image contrast enhancement according to published methods^22^, as shown in Supplementary Fig. S5b. Lymph node tissues served as positive controls to assess the staining efficacy of the IMC panel (Supplementary Fig. S5c). Following confirmation of the staining efficacy, the CRC tissues were processed using the identical IMC staining protocol. Typical regions of ROIs in CRC tissues, marked by DNA and staining markers, were clearly visible in the scanned images (Supplementary Fig. S6). Finally, cell segmentation^23–25^ and phenotyping were performed, followed by spatial analysis. (Fig. 2a).

In general, we identified 314,774 high-quality cells with accompanying spatial information. The cells were further clustered according to the presented lineage and functional markers. The clustering and sub-population strategies are shown in Supplementary Fig. S7a. Briefly, Rphenograph clustering method was applied to identify the 15 major cell clusters with the lineage markers (Fig. 2b; Supplementary Fig. S7b). Uniform manifold approximation and projection (UMAP) demonstrated the expression patterns of Pan-CK (tumor cell marker), CD3 and CD45RO (memory T cell markers), CD20 (B cell marker), CD15 (one of the granulocyte markers), and CD68 and HLA-DR (macrophage markers) (Supplementary Fig. S7b). We subsequently conducted unsupervised clustering to identify distinct subpopulations within monocytes and macrophages based on the expression of the associated lineage and functional markers (Supplementary Fig. S7c). This analysis revealed a total of four cell subclusters among monocytes/macrophages, including infiltrating-like classic monocytes, intermediate status monocytes, CD163^*−*^C1QC^median^ resident-tissue macrophages (CD163^*−*^C1QC^median^ RTMs), and C1QC^+^ RTMs (Fig. 2b; Supplementary Fig. S7c). Natural killer (NK) and T cells were grouped into 14 subclusters using activation and immune checkpoint markers, including cytotoxic and memory CD8^+^ T cells, Tregs, PD1^+^CD4^+^ T cells, and TIM3^+^ CD4^+^ T cells (Fig. 2b; Supplementary Fig. S7d). We also identified B cells marked with CD20, fibroblasts marked with collagen I and several other cell types (Supplementary Fig. S7e). Finally, a total of 30 cell populations with distinct expression patterns were identified in our spatial immune atlas (Supplementary Fig. S7e). The proportions of major cell types and immune cell subtypes among all cells were separately presented (Fig. 2b, c). The cellular composition of all ROIs, based on manual annotations, is presented in Fig. 2d and Supplementary Figs. S8, S9, offering a detailed representation of the proportions of immune, stromal, and epithelial cells. These figures further provide a precise depiction of the TME.

In the next step, we used t-Distributed Stochastic Neighbor Embedding (t-SNE) (Supplementary Fig. S10a) and hierarchical clustering (Supplementary Fig. S10b) on the basis of the IMC data to determine the degree of variation among the four groups. Both t-SNE and hierarchical clustering showed significant stratification among the four patient groups. The Ro/e analysis^26^ was further performed to quantify the enrichment tendency of the cell clusters among patients in different subgroups. Consistent with a previous report^27^, it was found that the C1QC^+^ RTMs were preferentially enriched in the MSI group compared with the MSS group (Supplementary Fig. S10c). Additionally, they were enriched in the R group compared to the NR group (Supplementary Fig. S10d). Furthermore, we observed that the proportion of C1QC^+^ RTMs was the greatest in the MSI-R subgroup, followed by the MSS_R, MSI-NR, and MSS_NR subgroups (Fig. 2e). In contrast, we observed a significant enrichment of fibroblasts in non-responders, regardless of microsatellite status (Fig. 2e). To account for both individual sample variability and population-level variability, and to ensure statistical robustness, we performed additional analyses, including χ2 tests, sample-level *t* tests, odds ratio (OR) analysis^28^, and Milo differential abundance testing^29^ (Supplementary Figs. S11, S12). Furthermore, we computed the cell frequency of cell clusters in each genomic phenotype (MSS/MSI) and investigated their associations with the immunotherapy response (Fig. 2f). Consistent with the results from the Ro/e analysis, the cell frequency of C1QC^+^ RTM exhibited a robust correlation with the MSS_R phenotypes calculated by logistic regression.

Lastly, we analyzed the spatial TME ecosystem on the basis of the IMC output images (Fig. 2g). We observed that the differences in the TME between MSS_R and MSS_NR were more pronounced than those between MSI_R and MSI_NR (Fig. 2h). This suggests that the TME may have a greater impact on immunotherapy response in MSS patients compared to MSI patients. Specifically, the greater heterogeneity in the TME of MSS_R and MSS_NR groups highlights the potential role of TME composition in driving differential responses to immunotherapy within the MSS subtype. We then employed a ResNet18-based deep learning model (Materials and Methods for details) to validate the associations of C1QC^+^ RTMs and fibroblasts with the immunotherapy response by quantifying the importance ranking of features. The results indicated that DNA, αSMA, C1QC, Collagen I, CD45, and Vimentin were the most important features for stratifying the four groups (Fig. 2i). Hence, we believe that C1QC^+^ RTMs and fibroblasts may both act as important players in orchestrating the response to immunotherapy.

Taken together, the IMC analysis provided a spatially resolved view of the TME in ICI-treated MSS/MSI_R and NR CRC patients. We observed a greater proportion of C1QC^+^ RTMs, a subset of macrophages, in ICI-responsive CRC patients than in non-responsive patients, whereas a lower proportion of fibroblasts were detected in ICI-responsive patients.

### The spatial architecture of C1QC^+^ RTMs and fibroblasts orchestrates antitumoral immunity in MSI and MSS CRC patients

**Fig. 3:**
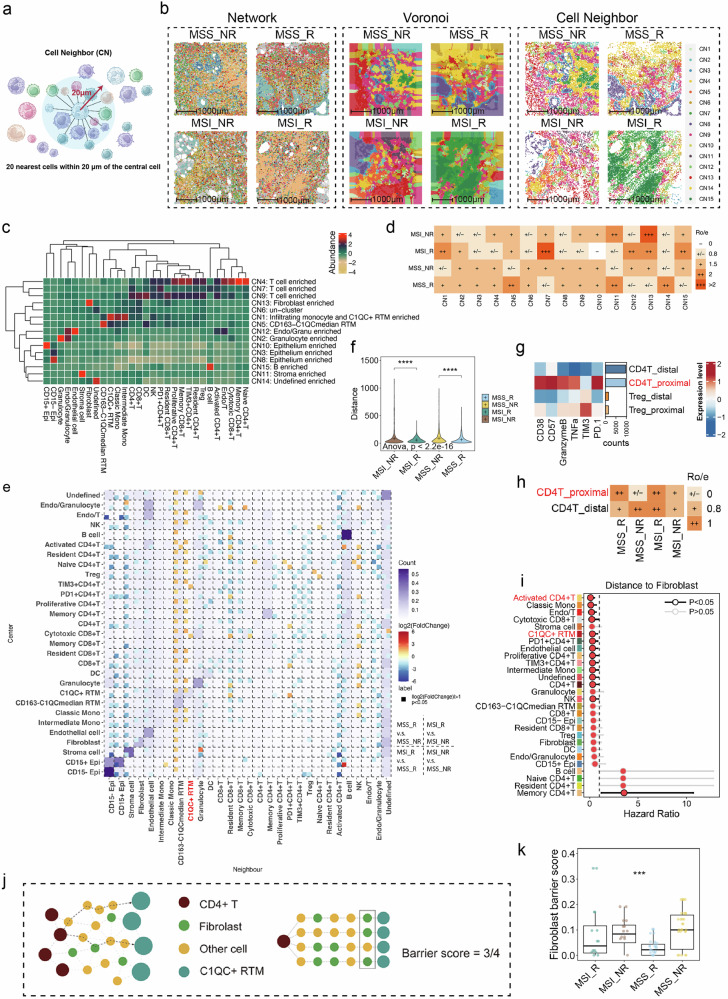
The spatial cell neighboring and cell–cell distance phenotype of MSS/MSI_R and NR CRC patients. **a** Schematic diagram of cell neighbor (CN) identification. **b** Representative Network, Voronoi and CN diagrams of the TME in MSS/MSI_R and NR CRC samples. **c** Heatmap of 15 distinct CNs based on the 29 original cell clusters and their respective abundances within each CN. **d** Tissue prevalence of each CN cluster estimated by the Ro/e analysis. **e** Pairwise cell‒cell interaction heatmap. The background color represents overall interaction count intensity across all samples, with darker shades indicating higher interaction intensities. Small squares denote statistically significant interactions in comparisons between different patient groups, with their color representing the log2 fold change (log2FC) in interaction strength. **f** Violin plots illustrating the distances between C1QC^+^ RTM and CD4^+^ T cells across the four groups. Significance between the two groups was evaluated by the *t* tests with ****P < 0.0001. The one-way ANOVA test was used to compare the four groups. **g** Heatmap displaying the expression levels of T cell-related markers (e.g., CD38, CD57, GZMB, TNFα, PD-1) in CD4⁺ T cells proximal to C1QC⁺ RTMs vs those distal to C1QC⁺ RTMs, colored by z-score normalized expression levels. **h** Ro/e analysis of CD4⁺ T cells proximal to C1QC⁺ RTMs and distal to C1QC⁺ RTMs in the four groups. **i** The distance of immune cell subsets to fibroblasts associated with the prognosis of CRC. The Hazard Ratio and *P* value was calculated on data from IMC by univariate Cox analysis. (**j**) Schematic diagram of fibroblast barrier score calculation. The barrier score measures the degree of spatial interpositioning of C1QC^+^ RTM–adjacent fibroblasts between CD4^+^ T cells and their nearest C1QC^+^ RTM (s) in a tissue core. In the lower half of the schematic, four nearest C1QC^+^ RTMs are defined for the purple CD4^+^ T cells. C1QC^+^ RTMs–adjacent fibroblasts are found on three of these four paths from CD4^+^ T-cell to C1QC^+^ RTMs, resulting in a barrier score of 3/4. (**k**) Boxplot comparing the fibroblast barrier scores in the four groups. Statistical significance was determined by one-way ANOVA comparing MSI_R, MSI_NR, MSS_R, and MSS_NR groups.

Antitumor immunity requires organized, spatial interactions between tumor, immune, and stromal cells and extracellular components of the TME^30^. To generate higher-order information beyond cell type and abundance, we conducted a cellular neighborhood (CN) analysis. The CN was defined as a central cell and its 20 nearest neighboring cells (Fig. 3a). To analyze cell interactions and functional units within MSS/MSI CRC samples, we employed network, Voronoi, and CN plots derived from IMC images (Fig. 3b). We systematically evaluated various values of k (the number of neighborhoods, k = 5, 10, 15, and 20) to identify the optimal clustering resolution, ultimately selecting k = 15 as it provided the best balance between granularity and biological interpretability (Fig. 3c; Supplementary Fig. S13). The spatial distribution and visualization of CNs within the TME for each MSS and MSI sample are presented in Supplementary Figs. S14 and S15, offering a comparative analysis of CN patterns across different response groups. Specifically, CN1 was highly abundant in the MSI_R subgroup, less abundant in the MSS_R and MSI_NR subgroup, and sparsely present in the MSS_NR subgroup (Fig. 3d; Supplementary Fig. S16). We also found that the MSI_NR subgroup had a high abundance of CN13, which was enriched with fibroblasts.

To explore the underlying interactions of the cell populations in the TMEs of the four groups, we conducted a spatial analysis of paired cell–cell interactions (PCIs), which was driven by spatial proximity between cells. Compared with those in the MSS_NR group, CD4^+^ T cell populations, such as resident CD4^+^ T cells, naïve CD4^+^ T cells, and memory CD4^+^ T cells, showed significant interactions with C1QC^+^ RTMs in the MSS_R group (Fig. 3e), highlighting the spatial proximity between C1QC^+^ RTMs and CD4^+^ T cells in MSS_R. Consistent with the results of the PCI analysis, the distances between C1QC^+^ RTMs and CD4^+^ T cells were significantly lower in the R groups than in the NR groups (Fig. 3f), further supporting their spatial colocalization and potential interaction in responders. Further survival analysis revealed that patients with shorter median distances between C1QC^+^ RTMs and CD4^+^ T cells presented a greater survival benefit than those with longer median distances (Supplementary Fig. S17a). Based on the spatial distances between C1QC⁺ RTMs and CD4⁺ T cells, we categorized CD4⁺ T cells into two groups: those proximal to C1QC⁺ RTMs and those distal to C1QC⁺ RTMs. The heatmap demonstrates that CD4⁺ T cells located proximal to C1QC⁺ RTMs display significantly elevated expression of activation markers, such as CD38, CD57, GZMB, TNFα, and PD-1, relative to their distal counterparts (Fig. 3g). Furthermore, Ro/e analysis indicates that responders, especially within the MSS subgroup, exhibit a greater proportion of proximal CD4⁺ T cells, implying that spatial proximity to C1QC⁺ RTMs may potentiate CD4⁺ T cell activation (Fig. 3h). In the next step, we measured the distances from fibroblasts to immune cells (Fig. 3i). Notably, we discovered that a shorter distance between certain CD4^+^ T cell subsets (e.g., activated CD4^+^ T cells) and fibroblasts, was correlated with poorer outcomes (Fig. 3i; Supplementary Fig. S17b). The same tendency was also observed between C1QC^+^ RTMs and fibroblasts (Fig. 3i; Supplementary Fig. S17c). Lastly, we constructed a cellular spatial graph for each sample to derive a barrier metric based on fibroblasts that impedes C1QC^+^ RTMs and CD4^+^ T cells (Fig. 3j). The MSS_NR and MSI_NR groups displayed significantly higher barrier scores attributed to fibroblasts (Fig. 3k), leading to the spatial segregation of C1QC^+^ RTMs by fibroblasts in non-responders.

In summary, we conducted CN, PCI, and cell distance analyses to study the TME characteristics in MSS/MSI CRC patients with different immunotherapy responses. Our findings suggest active communication between C1QC^+^ RTMs and T cells in responders. Furthermore, we observed greater distances between C1QC^+^ RTMs and T cells, in MSS/MSI_NR tumors than that in MSS/MSI_R tumors, which may be attributed to the barrier effect of fibroblasts. These observations may indicate specific cellular interactions and relationships within the TME that are in accordance with the general understanding of the immunotherapy response.

### The scRNA-seq analysis indicates the antigen presenting role of C1QC^+^ RTMs to CD4^+^ T cells via upregulated MHC class II signaling

**Fig. 4:**
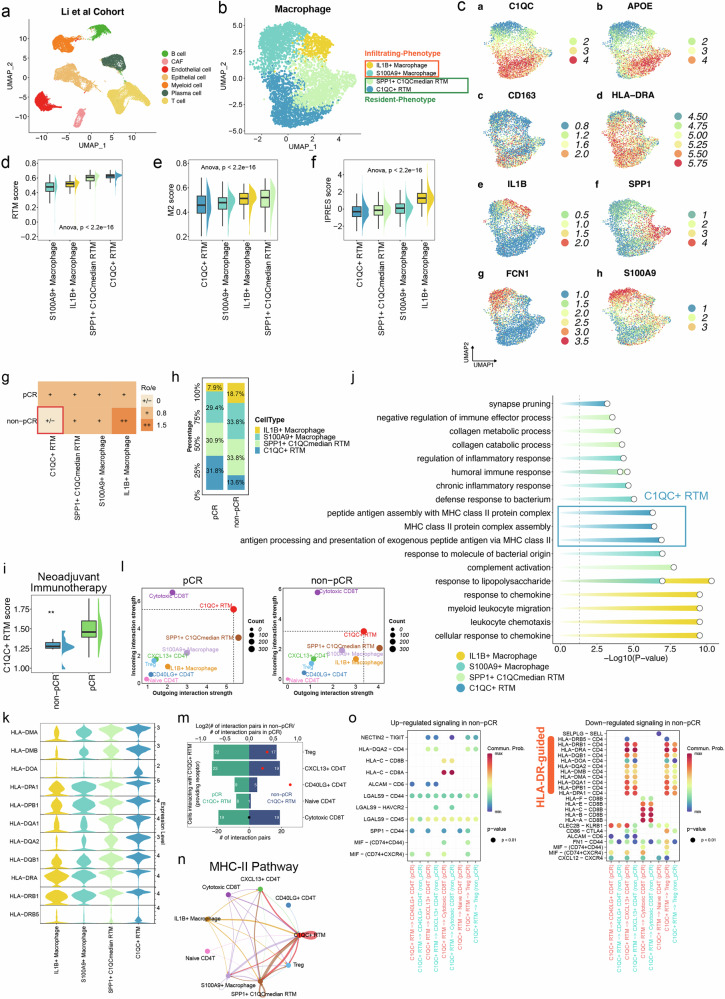
scRNA-seq analysis indicates C1QC^+^ RTM-mediated immune activation via MHC class II signaling. **a** UMAP plot of broad cell types from the Li et al. cohort. **b** UMAP plot of the macrophages showing transcriptionally distinct clusters. **c** UMAP plots showing the expression of selected marker genes in macrophages. **d**–**f** The Resident tumor macrophage (RTM) score, M2 score and innate anti-PD-1 resistance (IPRES) score in distinct cell clusters. The one-way ANOVA test was adopted to evaluate the statistical significance. **g** Abundance of each macrophage cluster in the tissue of pCR and non-pCR groups was estimated via Ro/e analysis. **h** Cell cluster frequency shown as a fraction of total macrophages in pCR and non-pCR group. **i** C1QC^+^ RTM score calculated by single sample gene set enrichment analysis (ssGSEA) method on the basis of bulk transcriptome from pCR and non-pCR group. The Wilcoxon Rank-Sum Test was adopted to evaluate the statistical significance. **j** GO analysis of C1QC^+^ RTMs and the other three macrophage clusters. **k** Violin plot showing the expression levels of MHC-II molecules in C1QC^+^ RTMs and the other three macrophage clusters. **l** The outgoing and ingoing interaction strength of immune cells in pCR and non-pCR group. The *x*-axis and *y*-axis scales differ between the pCR and non-pCR groups. **m** The number of pair–ligand interactions between T cells and C1QC^+^ RTM in the pCR and non-pCR groups. **n** Differences in the MHC-II pathway interaction of various cell types. The thicker the line, the stronger the connection. **o** Up-regulated and down-regulated receptor–ligand pairs that differ significantly between pCR and non-pCR based on C1QC^+^ RTM and other cell clusters. Dot size indicates the *P* value, colored according to the communication probability of pathways.

To further investigate the role of C1QC^+^ RTMs in immunotherapy, we performed scRNA-seq analysis on 27 CRC tissue samples (22 pathological complete response (pCR) and 5 non-pCR), which were obtained from 19 patients who underwent neoadjuvant therapy, as reported by Li et al. ^31^ (Fig. 4a). To ensure consistency with our IMC antibody panel and in-house IMC data, as well as to further explore the functional characteristics of the macrophage subsets identified in IMC at the high-throughput transcriptomic level, we re-annotated the macrophage subsets in the published scRNA-seq dataset based on established marker genes, including *SPP1* and *C1QC* for functionally distinct macrophage populations^10,11,32^, as well as *IL1B* and *S100A9*, which define inflammatory and immunosuppressive monocyte/macrophages^11,33–35^, respectively (Supplementary Fig. S18a). We successfully identified C1QC^+^ RTMs in the scRNA-seq data. This subset highly expressed C1QC and CD163 (Fig. 4b, c; Supplementary Fig. S18a), and matched resident macrophages^36–38^, corresponding to C1QC^+^ RTMs identified in IMC. Additionally, we obtained three other major macrophage cell clusters, including IL1B^+^ macrophages, S100A9^+^ macrophages, and SPP1^+^ C1QC^median^ RTMs (Fig. 4b), with distinct gene expression patterns (Supplementary Fig. S18a and Table S4). Among them, IL1B^+^ macrophages and S100A9^+^ macrophages exhibited an infiltrating cell phenotype. In contrast, SPP1^+^C1QC^median^ RTMs displayed a resident cell phenotype. Specifically, IL1B^+^ macrophages expressed the inflammatory response gene *IL1B*^35^ (Fig. 4c; Supplementary Fig. S18a). S100A9^+^ macrophages, which we identified previously^34^, expressed the extracellular matrix-associated genes *S100A9* and *FCN1* (Fig. 4c; Supplementary Fig. S18a). SPP1^+^C1QC^median^ RTMs expressed the lipid metabolism gene *APOE* and pro-angiogenic gene *SPP1*. Additionally, this subset exhibited low expression of *CD163* and moderate expression of *C1QC* (Fig. 4c; Supplementary Fig. S18a), similar to the phenotype of CD163^*−*^C1QC^median^ RTMs identified in IMC.

We calculated the RTM score for each subcluster on the basis of the core gene signature previously reported^37,39^. As shown in Fig. 4d, the C1QC^+^ RTMs had the highest RTM score (the gene set is shown in Supplementary Table S5), indicating the resident phenotype of C1QC^+^ RTMs. Next, we calculated the M2 score to evaluate M2 polarization in the four macrophage clusters. Among them, the C1QC^+^ RTMs presented the lowest M2 score (Fig. 4e; gene set shown in Supplementary Table S5). We also applied the innate anti-PD-1 resistance (IPRES) signature (gene set shown in Supplementary Table S6)^40^ to evaluate the immunosuppressive status of the four macrophage subtypes and found that the C1QC^+^ RTMs had the lowest IPRES signatures and tended to be sensitive to immunotherapy (Fig. 4f). In summary, C1QC^+^ RTMs displayed a resident macrophage phenotype, which tends to be associated with the immunotherapy response. Next, to further analyze the distributions of the macrophage subpopulations, we performed a Ro/e analysis. This analysis revealed greater infiltration of C1QC^+^ RTMs and a lower infiltration of IL1B^+^ macrophages in the pCR group than in the non-pCR group (Fig. 4g). The cell proportion analysis also showed that compared with the non-pCR samples, the pCR samples presented a greater proportion of C1QC^+^ RTM (31.8% vs 13.6%) and a lower proportion of IL1B^+^ macrophages (7.9% vs 18.7%) (Fig. 4h). Subsequently, the top 20 genes with high expression in C1QC^+^ RTMs from the scRNA-seq analysis were defined as the C1QC^+^ RTM signature which demonstrated high specificity in distinguishing C1QC^+^ RTMs from other macrophages (Supplementary Fig. S18 and Table S4). We further validated the role of C1QC^+^ RTM infiltration in pCR in a bulk transcriptome dataset^41^ using ssGSEA scoring based on the C1QC^+^ RTM signature. The results showed that the C1QC^+^ RTM score was significantly greater in the pCR group than in the non-pCR group among CRC patients who were receiving neoadjuvant immunotherapy (Fig. 4i).

Next, we sought to explore the potential functions and relevant pathways of C1QC^+^ RTMs and the other three macrophage clusters. Gene Ontology (GO) analyses provided evidence of the immunogenicity-enhancing effects of C1QC^+^ RTMs, mainly related to increasing expression of pathways pertaining to antigen processing via MHC-II signaling (Fig. 4j). MHC-II molecules are expressed by macrophages that deliver antigens to CD4^+^ T cells and play an important role in adaptive immunity^42^. As shown in Fig. 4k, the C1QC^+^ RTMs expressed high levels of MHC-II molecules such as *HLA-DMA*, *HLA-DMB*, *HLA-DQA1* and *HLA-DRA*. Next, we identified T cell clusters such as CD40LG^+^CD4^+^ T cell, CXCL13^+^CD4^+^ T cell, cytotoxic CD8^+^ T cell, naïve CD4^+^ T cell, and Treg cell clusters (Supplementary Fig. S19a) and their top marker genes (Supplementary Fig. S19b) for further cell–cell interaction analysis. Our analysis revealed that the interaction strength of C1QC^+^ RTMs with other immune cells was greater in pCR tumors than in non-pCR tumors (Fig. 4l). Specifically, the C1QC^+^ RTMs in pCR tumors showed greater interaction activities with CD4^+^ T cells, including CXCL13^+^CD4^+^ T cells, CD40LG^+^CD4^+^ T cells, naïve CD4^+^ T cells, and Treg compared to the C1QC^+^ RTMs in non-pCR tumors (Fig. 4m, n). Furthermore, we observed that certain ligand-receptor pairs related to C1QC^+^ RTM and T cells were dysregulated in non-pCR samples. For instance, NECTIN2-TIGIT and LGALS9-HAVCR2 pairs were upregulated in non-pCR samples, while HLA-DR guided pairs such as HLA-DRB5-CD4, HLA-DOA-CD4, and HLA-DQA1-CD4 were downregulated in non-pCR samples (Fig. 4o), indicating a potential defect in antigen presentation and T cell activation in this group. Pathway activity analysis further showed reduced activation, antigen presentation, and immune signaling in CD4⁺ T cells, Tregs, and cytotoxic CD8⁺ T cells in non-pCR samples (Supplementary Fig. S19c). The expression of key T cell activation and differentiation markers in CD4^+^ T cells, including *CD69*, *CD40LG*, *CD28*, *TNF*, *IL7R*, *CCR7*, *LEF1*, and *TCF7*, was significantly lower in non-pCR patients, indicating impaired CD4^+^ T cell function (Supplementary Fig. S19d, e). These findings collectively highlight a weakened CD4^+^ T cell-mediated immune response in non-pCR patients, suggesting impaired anti-tumor immunity.

Taken together, these findings suggest that C1QC^+^ RTMs may promote antitumor immunity in CRC patients receiving ICI treatment by enhancing their interaction with CD4^+^ T cells through upregulation of MHC-II molecule expression.

### Paired spatial resolved proteomics analysis confirms the strong APC function of C1QC^+^ RTMs in immunotherapy

To enhance our comprehension of the spatial diversity and possible roles of the proteome in MSI/MSS_R and NR CRC tissues, we have devised a method that integrates manual tissue microdissection by increasing the spatial resolution through tissue expansion with bottom-up mass spectrometry (MS)-based proteomic analysis^43^. This technique involves sequentially slicing tissue samples and conducting both IMC and spatial proteomics analysis to examine the molecular features within the ROIs of IMC (Supplementary Fig. S20a). The tumor tissues were sequentially sectioned, with adjacent pairs selected for processing. One section was stained with hematoxylin and eosin (H&E) for ROI selection, while the other was utilized for IMC analysis. The H&E-stained sections were subsequently used for spatial proteomics investigations. Following H&E staining, tissue expansion was conducted, and the chosen ROIs were subsequently expanded for dissection. These dissected ROIs were then subjected to LC‒MS/MS analysis. Through the integration of IMC and spatial proteomics methods, we were able to elucidate molecular features. As the demo shown in Supplementary Fig. S20a, we chose the exact same 50 ROIs consistent with the IMC outputs in 25 samples. Microdissection was performed via tissue expansion, which was executed after the tissue stained with H&E and embedded in a hydrogel. Next, peptide extraction and analysis was performed using a timsTOF Pro mass spectrometer in parallel accumulation-serial fragmentation combined with data-independent acquisition (diaPASEF) mode. MS data quality control demonstrated robust reproducibility in the coefficient of variation and Pearson correlation analysis for global precursors and proteins (Supplementary Fig. S20b, c). When examining pooled samples across different batches, we observed high consistency with pooled samples (Supplementary Fig. S20d, e). We have thus confirmed that integrated IMC and spatial proteomics analysis provides detailed protein information underlying the TME components.

**Fig. 5:**
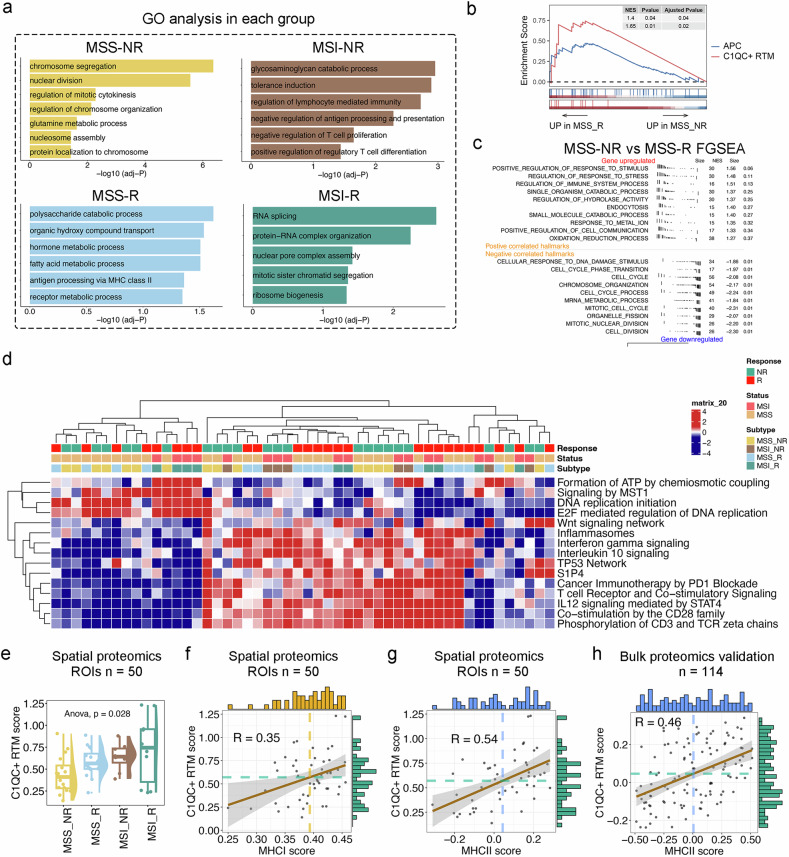
Paired spatial resolved proteomics confirm the antigen presenting role of C1QC^+^ RTMs in immunotherapy. **a** The potential biological functions and relevant signaling pathways of MSS/MSI_R and NR CRC patients were evaluated by the GO analyses. The hypergeometric test for over-representation was adopted to evaluate the statistical significance with multiple tests corrections. **b** Gene set enrichment analysis (GSEA) enrichment for APC and C1QC^+^ RTMs in MSS_NR and MSS_R samples. NES, normalize enrichment score. The Kolmogorov-Smirnov test was adopted to evaluate the statistical significance with multiple tests corrections. **c** Fast gene set enrichment analysis (FGSEA) enrichment for top 10 upregulated pathways and top 10 downregulated pathways comparing MSS_NR and MSS_R according to the hallmark gene sets. **d** Heatmap showing different immunotherapy-related pathways enriched in the integrated MSS/MSI_R and NR groups by gene set variation analysis (GSVA) analysis, colored by z-score transformed mean GSVA scores. **e** Boxplot illustrating the C1QC^+^ RTM scores, calculated using the C1QC^+^ RTM signature, in spatial proteomics analyses. The one-way ANOVA test was adopted to evaluate the statistical significance. **f** Scatter plots showing Pearson’s correlation between C1QC^+^ RTM and MHC-I score, calculated using the C1QC^+^ RTM signature and MHC-I signature, in spatial proteomics analyses. **g**, **h** Scatter plots depicting the Pearson’s correlation between C1QC^+^ RTM and MHC-II scores, as determined through spatial proteomics analyses and further validated through bulk proteomics. The Pearson Coefficient Test was adopted to evaluate the statistical significance.

The highly expressed proteins were determined within each group, and subsequent GO analyses were performed to explore the potential biological functions (Fig. 5a). The upregulated proteins in the MSI_R group were associated with RNA splicing, protein–RNA complex organization, and nuclear pore complex assembly. Interestingly, we observed heightened activities of negative regulation of antigen processing and presentation, negative regulation of T cell proliferation, and positive regulation of regulatory T cell differentiation in MSI_NR samples. Moreover, the upregulated proteins in the MSS_R group were involved in polysaccharide catabolic processes, antigen processing via MHC-II, and hormone metabolism. Lastly, the biological processes of MSS_NR are centered around chromosome segregation, nuclear division, and the regulation of mitotic cytokinesis. Therefore, we investigated the expression patterns and signaling pathways differences in MSS_R/NR samples via GSEA. The MSS_R showed enrichment of the APC and C1QC^+^ RTM signatures (Fig. 5b). Furthermore, the genes whose expression was upregulated in MSS_R samples were associated with pathways related to immune process regulation pathways, whereas those whose expression was downregulated were associated with pathways related to the response to DNA damage stimulus and the cell cycle (Fig. 5c). The unsupervised immunotherapy-related pathway enrichment analysis highlighted the intratumoral heterogeneity observed among CRC subgroups, including the MSS/MSI subgroups and immunotherapy R/NR subgroups (Fig. 5d). In general, samples from R groups showed more enriched pathways related to inflammasomes, interferon gamma signaling, cancer immunotherapy by PD-1 blockade, T cell receptor and co-stimulatory signaling, IL-12 signaling mediated by STAT4, and co-stimulation by the CD28 family. Signals emanating from inflammasomes, and T cell co-stimulatory receptors are thought to influence the fate and effector functions of CD8^+^ T cells^44,45^. The IL12/JAK2/STAT4 pathway is considered to significantly activate effector T cells^46^. Moreover, the costimulatory receptor CD28 is essential for adaptive immunity against malignant tumor^47^. We subsequently evaluated the C1QC^+^ RTM score in the four groups of CRC patients. The C1QC^+^ RTM score significantly differed among the groups (*P* = 0.028), with the highest score observed in the MSI_R and the lowest in the MSS_NR (Fig. 5e). To further assess the functionality of C1QC^+^ RTMs, we calculated the Pearson correlation coefficient between the C1QC^+^ RTM score and the MHC I/II score (the gene set is shown in Supplementary Table S7). Our analysis revealed a positive but moderate correlation between the C1QC^+^ RTM score and MHC I expression (Pearson correlation coefficient (R) = 0.35) (Fig. 5f). In contrast, there was a notably stronger positive correlation between the C1QC^+^ RTM score and MHC II expression (R = 0.54) (Fig. 5g), which was further confirmed in an additional in-house set^48^ of 114 bulk proteomics samples (R = 0.46) (Fig. 5h).

Overall, our findings underscore the distinct abundance of C1QC^+^ RTM across the four groups through spatial proteomics analysis, which corroborates the data obtained from IMC analysis. Furthermore, the new proteomics analysis provides compelling evidence supporting a correlation between increased APC activities mediated by MHC II and the abundance of C1QC^+^ RTMs. This finding further strengthens and validates the results obtained from the scRNA-seq and IMC analyses.

### CRISPR-Cas9-mediated genome-wide knockout screen identifies regulators and pathways involved in modulating the antigen presentation function of C1QC^+^ RTMs

**Fig. 6:**
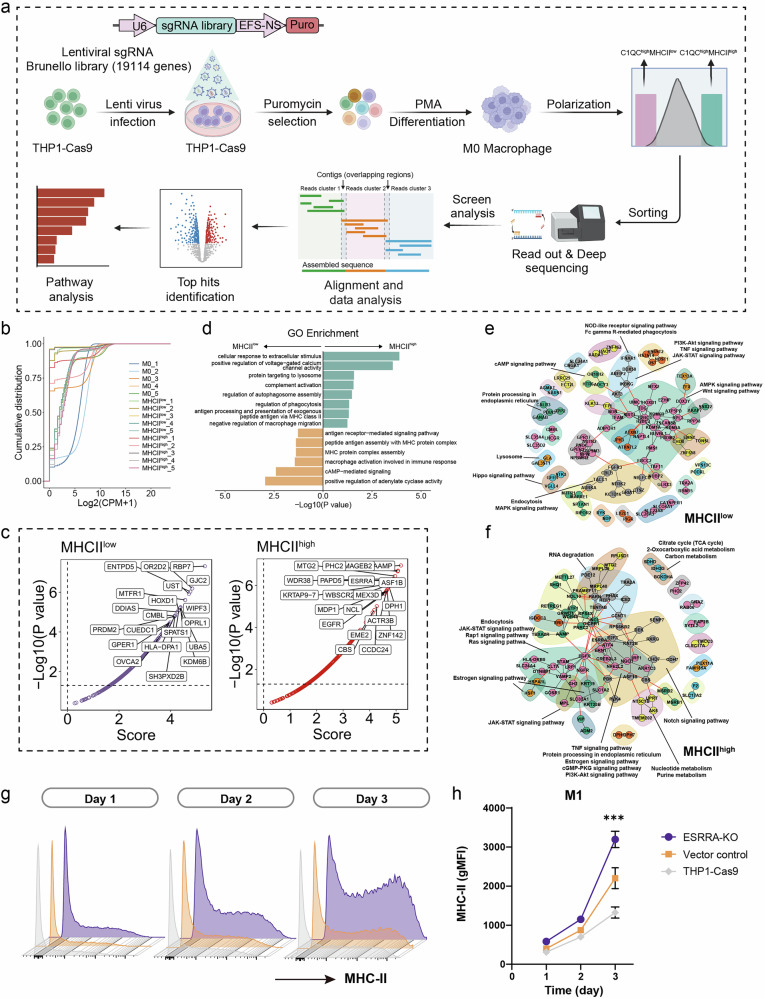
Whole genome screening detecting the potential related pathways involved in regulating APC in C1QC^+^ RTMs. **a** Schematic representation of the workflow for the genome-wide CRISPR/Cas9 screen. **b** Cumulative distribution function (CDF) of biological replicates of 5 representative samples of M0, MHC^low^, and MHC^high^ cells. CPM, counts per million. *n* = 5 in each group. **c** Top hit genes of MHC^low^ and MHC^high^ cells. The *P* value was corrected by Benjamini-Hochberg test. **d** Gene ontology (GO) term analysis of MHC^low^ and MHC^high^ cells. **e**, **f** KEGG gene interaction network of the hit genes in MHC^low^ and MHC^high^ cells. Subnetworks (Neighborhoods) are colored and annotated with enriched functional categories. Gray lines, connections within a neighborhood; red lines, connections between neighborhoods. **g**, **h** Representative flow cytometric plot and quantification of MHC-II expression levels in *ESRRA*-KO THP-1 cells during macrophage polarization. Vector control and THP-1-Cas9 were used as control for comparison. Vector control indicates THP-1 cells infected with lentivirus carrying an empty plasmid lacking gRNA. gMFI, geometric mean fluorescence intensity. *P* values in **h** were determined using one-way ANOVA.

To identify the genes and pathways involved in the antigen presentation function of C1QC^+^ RTMs, we performed a CRISPR-Cas9-mediated genome-wide knockout screen using the Brunello library, which comprises 76,441 single guide RNAs (sgRNAs) targeting 19,114 genes (Fig. 6a). We first generated THP-1 cell lines that constitutively expressed Cas9. Next, the cells were subjected to transduction with lentivirus containing the Brunello library at a multiplicity of infection (MOI) of 0.3 to ensure that the cells were individually infected. Two days after transduction, we started selection with puromycin for 5–6 days to isolate infected cells and to reach 400-fold coverage. The cells were then differentiated into M0 macrophages following PMA priming and further polarized with IFNγ + LPS. At the endpoint, two distinct groups of macrophages with high C1QC expression, C1QC^high^MHC-II^high^ macrophages and C1QC^high^MHC-II^low^ macrophages, were harvested for next-generation sequencing (NGS) to identify enriched sgRNAs and corresponding mutant genes. Biological replicates across all three cell clusters demonstrated high sequencing quality on the basis of cumulative distribution function plots (Fig. 6b). Deep sequencing revealed notable gene hits, including *RBP7*, *UST*, *OPRL1*, *HOXD1* and other genes in the C1QC^high^MHC-II^low^ macrophage populations, and *ESRRA*, *MEX3D*, *ASF1B*, *DPH1, MAGEB2, ACTR3B* and other genes in the C1QC^high^MHC-II^high^ macrophage populations (Fig. 6c). GO analysis of these hits from both C1QC^high^MHC-II^high^ and C1QC^high^MHC-II^low^ macrophage populations revealed significant enrichment in terms related to antigen-presentation capacity (APC) and macrophage activity, such as complement activation, the regulation of phagocytosis, peptide antigen assembly with the MHC protein complex, and antigen receptor-mediated signaling pathways (Fig. 6d). These findings validate the robustness of our CRISPR-Cas9-mediated genome-wide knockout screening. Subsequent Kyoto Encyclopedia of Genes and Genomes (KEGG) and network analyses elucidated the pathway networks involved in APC regulation in C1QC^+^ macrophages. The results showed that, following genome-wide knockout, lysosome pathways, the cAMP signaling pathway, the Hippo signaling pathway, MAPK signaling pathways, and endocytosis were involved in MHC-II^low^ macrophage populations (Fig. 6e). In contrast, the Notch signaling pathway, endocytosis pathway, the JAK-STAT signaling pathway, and the estrogen signaling pathway were associated with MHC-II^high^ macrophage populations after genome-wide knockout (Fig. 6f). Hence, the pathways identified in our screen were potentially involved in regulating APC function in C1QC^+^ macrophage populations. Notably, *ESRRA*, an orphan nuclear receptor implicated in cancer progression and resistance to immunotherapy, was identified as a key hit^49,50^. Previous studies by Sahu et al. have shown that *ESRRA* inhibitor can stimulate cytokine secretion, induce proinflammatory macrophage polarization, and enhance antigen-presentation, thereby recruiting T cells into tumors^50^. To validate our genome-wide screening results, we specifically knocked out *ESRRA* in THP-1 cells and subjected them to M1 polarization with IFNγ + LPS. Compared with the vector control and pHK14-Cas9 groups, the *ESRRA* KO cells exhibited significantly higher MHC-II expression (Fig. 6g, h). Collectively, the whole genome screening identifies critical genes and pathways involved in regulating APC function in C1QC^+^ macrophages.

### Spatial transcriptomics reveals the colocalization between C1QC^+^ RTMs and CD4^+^ T cells in the CRC TME of responders

**Fig. 7:**
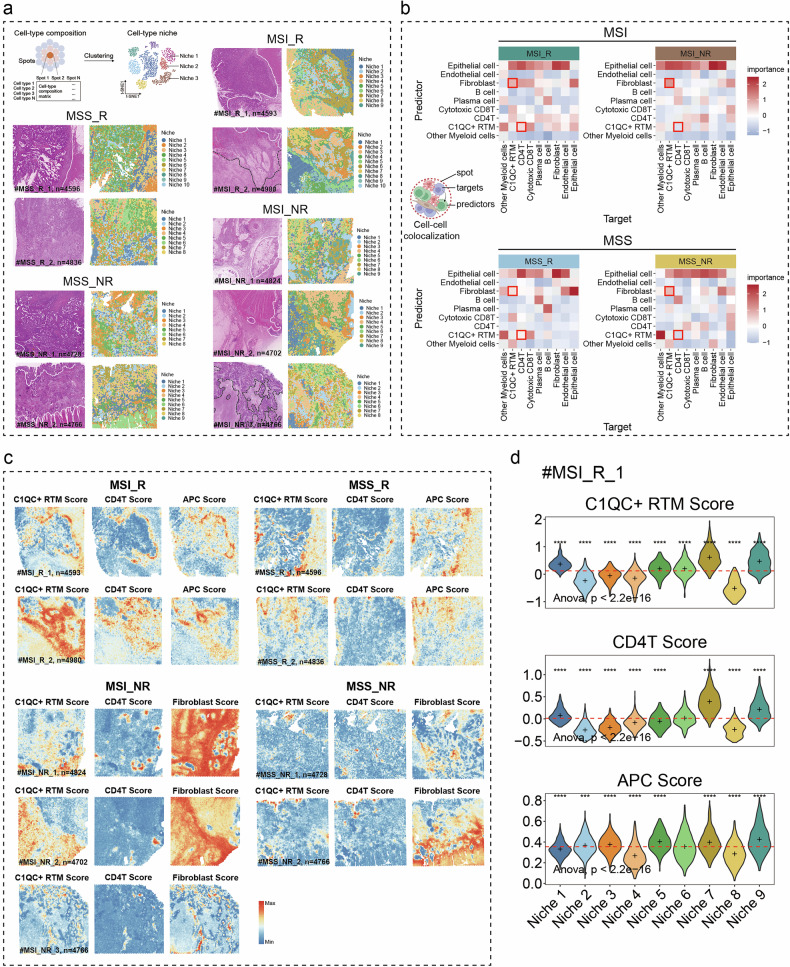
Differences in the co-localization of C1QC^+^ RTMs and CD4^+^ T cells between R and NR CRC patients revealed by spatial transcriptomics. **a** Schematic diagram of niche computation, H&E staining of each sample, and the spatial map of niches. The white dashed line in the H&E section separated the para tumor from the tumor, and the black dashed line separated the tumor from the necrotic region. **b** Co-localization analysis using the MISTy algorithm. Median importance of cell-type abundance in predicting the abundances of other cell types within a spot for MSI_R (top left), MSI_NR (top right), MSS_R (bottom left), and MSS_NR (bottom right). **c** Spatial feature plots showing signature scores for MSI_R, MSI_NR, MSS_R, and MSS_NR samples. **d** Violin plots illustrating the gene set scoring results for each niche in the MSI_R_1 sample.

Owing to the limitations of IMC, the investigation of the relationship between C1QC^+^ RTMs and CD4^+^ T cells was restricted to a small spatial area. Nevertheless, we aimed to examine the interaction between C1QC^+^ RTMs and CD4^+^ T cells at the broader histological level of CRC. To achieve this, we performed spatial transcriptomic analysis on 9 CRC samples from an independent in-house cohort. CRC tissues were obtained before the initiation of immunotherapy, and patients were stratified based on their treatment response post-immunotherapy (MSS_NR, *n* = 2; MSS_R, *n* = 2; MSI_NR, *n* = 3; MSI_R, *n* = 2). The spatial expression patterns of key lineage markers in each sample are shown in Supplementary Fig. S21. Next, we integrated spatial transcriptomic data with scRNA-seq data using the deconvolution method, robust cell type decomposition (RCTD)^51^, to quantify the proportions of different cell types within each spot. Based on the spot-cell type composition matrix, unsupervised clustering was performed to identify the cellular niches within each sample (Fig. 7a). We delineated the cellular composition of these niches across different samples (Supplementary Fig. S22), representing potential structural building blocks among tissue samples. Additionally, to uncover the spatial distribution patterns of TME cells in different regions, we analyzed the intensity of various TME cell types both inside and outside the tumor region (Supplementary Fig. S23), with tumor boundaries defined by expert pathologists.

To further explore the spatial dynamics of cellular interactions in CRC, we applied the MISTy algorithm^52^ to assess whether the abundance of major cell types in individual transcriptomic spots could be predicted based on their spatial context, defined by the composition of neighboring cell types. Specifically, we assessed the importance of cell type abundance within a single spot (colocalization) in the tumor region. Our analysis revealed that, in both MSS and MSI CRC samples, responders exhibited a higher abundance of C1QC^+^ RTMs in the vicinity of CD4^+^ T cells compared to non-responders (Fig. 7b). Additionally, we observed that C1QC^+^ RTMs in non-responders were frequently surrounded by fibroblasts (Fig. 7b), suggesting a potential physical barrier role of fibroblasts in regulating the spatial distribution of C1QC^+^ RTMs. Furthermore, gene signature scoring (with gene lists provided in Supplementary Tables S4, S8) confirmed the colocalization of C1QC^+^ RTMs and CD4^+^ T cells in the tumor regions of responders, with overlapping increased scores and enhanced APC activity. In contrast, in non-responders, high C1QC^+^ RTM scores showed less pronounced overlap with high CD4^+^ T cell scores but correlated with elevated CAF scores (Fig. 7c, d; Supplementary Fig. S24).

Subsequently, to validate the colocalization of C1QC^+^ RTMs and CD4^+^ T cells, we analyzed publicly available spatial transcriptomic data from 7 CRC specimens^12,53,54^. Dimensionality reduction clustering based on the spot-gene expression matrix, combined with gene set scoring analysis (with gene lists provided in Supplementary Tables S4 and S8), revealed the colocalization of C1QC^+^ RTMs and CD4^+^ T cells across all samples (Supplementary Fig. S25a–n). More importantly, KEGG pathway enrichment analysis revealed that clusters enriched with C1QC^+^ RTMs and CD4^+^ T cells were significantly associated with antigen presentation and processing, phagosomes, and helper T cell differentiation, in contrast to other clusters (Supplementary Fig. S25o). We further spatially focused on the MHC-II signaling pathway network that mediates antigen presentation using CellChat^55^. Our analysis identified a substantial MHC-II signaling network in regions enriched with C1QC^+^ RTMs and CD4^+^ T cells, primarily driven by the HLA-DR family (Supplementary Fig. S25p). In addition, C1QC^+^ RTMs and CD4^+^ T cells in the tumor region interacted more significantly through the MHC-II signaling pathway than those in the normal region did (Slice2 and Slice3 in Supplementary Fig. S25p), indicating the enhanced APC of C1QC^+^ RTMs in the tumor region. These findings highlight the close spatial proximity of C1QC^+^ RTMs and CD4^+^ T cells in CRC tumors and suggest that their interaction, mediated by HLA-DR, may contribute to the establishment of an active TME.

In conclusion, the spatial transcriptome analysis further substantiated the findings obtained through IMC and spatial proteomics, confirming the close spatial relationship between C1QC^+^ RTMs and CD4^+^ T cells within CRC tumors. This association was more clearly observed in CRC tissues from immunotherapy-responsive patients regardless of microsatellite status. Notably, compared with R group tissues, NR group tissues were significantly enriched in fibroblasts that colocalized with C1QC^+^ RTMs. Therefore, we hypothesized that fibroblasts have the potential to impede the APC of C1QC^+^ RTMs–CD4^+^ T cell pairs.

### Detection of whole slide imaging location of CD4^+^ T cells and C1QC^+^ RTMs by multiplex immunofluorescence (mIF) staining

To evaluate the potential spatial interaction of CD4^+^ T cells and C1QC^+^ RTMs at the whole slide imaging (WSI) level, mIF staining was used to detect the spatial patterns of CD68, C1QC, HLA-DR, CD4 and αSMA. The co-localization of CD4^+^ T cells with C1QC^+^CD68^+^ cells was assessed by staining for CD4, C1QC, and CD68, and DAPI (Supplementary Fig. S26a). Spatial analysis with HALO^TM^ system was applied to measure the distance between C1QC^+^CD68^+^ cells and the nearest CD4^+^ T cells (Supplementary Fig. S26b). The results revealed that in the R groups, the proportion of co-localization (defined as a distance of less than 50 μm between C1QC^+^CD68^+^ cells and CD4^+^ T cells) was greater compared to the NR groups, confirming our data from IMC and spatial transcriptome analysis (Supplementary Fig. S26b, c).

### Pan-cancer survival analyses and immunotherapy response prediction according to the infiltration density of C1QC^+^ RTMs

Given our recognition of the pivotal role of C1QC^+^ RTMs in anti-tumor immune response, we next sought to evaluate the prognostic value of C1QC^+^ RTMs in cancer immunotherapy. We obtained 18 microarray datasets obtained from the Gene Expression Omnibus (GEO) database of 898 immunotherapy-treated cancer patients, including non-small cell lung cancer (NSCLC), skin cutaneous melanoma (SKCM), breast cancer (BRCA), esophageal cancer (ESCA), urothelial carcinoma (UC), renal cell carcinoma (RCC), and stomach adenocarcinoma (STAD) (Supplementary Table S9). Additionally, the difference in the abundance of C1QC^+^ RTMs between R and NR in each dataset was further evaluated using the gene signature determined in the scRNA-seq analysis. In general, the C1QC^+^ RTM abundance could be used to distinguish the R and NR groups in each dataset (Supplementary Fig. S27a). Meta-analysis further revealed that C1QC^+^ RTM infiltration was positively correlated with immunotherapy responsiveness in 14/18 independent datasets, including all NSCLC, RCC, STAD and GBM datasets and partial SKCM datasets (Supplementary Fig. S27b). The hazard ratio of C1QC^+^ RTMs in predicting R vs NR in all tumors was found to be 1.69 in both the common effect model and the random effects model. We subsequently conducted survival analysis across 7 datasets and found that greater infiltration of C1QC^+^ RTMs tended to be associated with longer survival (Supplementary Fig. S27c). The hazard ratios of C1QC^+^ RTMs contributing to impaired survival versus prolonged survival were 0.70 and 0.72 in the common effect model and random effects model, respectively. Collectively, we found that C1QC^+^ RTMs accumulate in immunotherapy responsive cancer tumor tissue samples and contribute to prolonged survival, which suggest its potential role as an effective prognostic marker in cancer immunotherapy.

## Discussion

Immunotherapy for mCRC has been disappointing, with only the MSI-H subtype demonstrating promising benefits. However, approximately 50% of MSI-H mCRC patients still exhibit primary resistance to immunotherapy^16^. In MSS mCRC, tumor regression has been observed in only 5%–20% of patients following combination immunotherapy^15,56^. We hypothesize that there may be commonalities between MSI-H resistant patients and MSS immunoinsensitive patients. Therefore, we designed this study to uncover the spatial microenvironment ecosystem in both immunotherapy-responding and non-responding MSS/MSI-H patients to identify spatial features associated with immunotherapy benefit.

The TME has been proven to play a role in the efficacy of immunotherapy in various tumor types. The TME of CRC has been extensively investigated in previous studies^31,34,57^. For instance, Huang et al. revealed that TAMs and granulocytic myeloid-derived suppressor cells may hinder immunotherapeutic efficacy by increasing the expression of immunoreceptor tyrosine-based inhibitory motif-bearing receptors, especially *SIRPA*^58^. Bahar et al. reported that the close proximity of PD-L1^+^ macrophages and PD1^+^ T cells is a potential predictive biomarker for the effectiveness of PD-1 blockade in MSI CRC^59^. Spatial topology information is typically lost during scRNA-seq analysis, but recent studies have employed spatial techniques to obtain the spatial information on the CRC TME^13,60^. A pivotal study by Bortolomeazzi et al. used IMC and mIF to analyze samples from MSI-H CRC patients included in the KEYNOTE-177 trial to investigate their response to immunotherapy. Their findings revealed that PD-L1^+^CD74^+^ macrophages were consistently the only immune cell subset associated with a durable response in CRC patients who benefited from immunotherapy^61^. These macrophages express PD-L1 and are closely situated to PD-1^+^CD8^+^ T cells, suggesting that the interaction between PD-1 and PD-L1 on these cells might impede CD8^+^ T cell activity^62^. However, the mechanism underlying the effectiveness of immunotherapy in some MSS patients is still poorly understood. In this study, we employed various techniques, including IMC, spatial proteomics, spatial transcriptome, scRNA-seq, bulk RNA-seq, and bulk proteomics, to comprehensively investigate the spatial topology of the TME landscape in patients with MSI/MSS who are responsive and non-responsive to immunotherapy at the single-cell level.

TAMs often act as immunosuppressive agents by secreting tumor-promoting factors and interacting with other cells, such as fibroblasts and tumor cells, within the TME. M1-phenotype macrophages are MHC-II-positive cells that are more active in initiating and promoting the immune response in tumors. The loss of the M1 phenotype of macrophages in the TME may lead to a loss of MHC-II molecules, impairing the anti-tumor capacity. RTM is a type of macrophage that gain increasing insights into TME of tumors. The RTMs play a vital role in maintaining tissue structure and integrity, both in normal and tumor tissues, owing to their high self-renewal capacity^63^. These cells are closely involved in regulating immunity, maintaining homeostasis, and promoting fibrosis within the TME. They have been reported to express specific markers such as C1QC, FOLR2, and MHC-II class molecules^37,64^. Recent studies have made the C1QC^+^ RTM a controversial prognostic biomarker and acts as a contradictory player in anti-tumor immunity. For instance, it has been reported that the high number of C1QC^+^ RTMs in RCC tumor is associated with higher postsurgical recurrence^65^. Zhang et al. also verified the negative association between survival time and the abundance of C1QC^+^ RTMs in hepatocellular carcinoma^66^. However, RTMs in triple-negative breast cancer have been proven to be positively correlated with T cell infiltration and favorable prognosis^37^. Importantly, C1QC^+^ TAMs in CRC are considered to interact with T cells and promote their recruitment and activation, resulting in prolonged survival^11^.

C1QC^+^ macrophages can be identified in the colon mucosa of ulcerative colitis and healthy individuals^67^, suggesting the resident phenotypes in the CRC TME. According to a study by Zhang et al. ^11^, C1QC^+^ macrophages are associated with complement activation as well as enriched antigen processing and presentation pathways. CSF1R blockade may selectively deplete C1QC^+^ macrophages. The elimination of C1QC^+^ macrophages featured with high APC capacity could potentially serve as an unrecognized mechanism of resistance against non-specific macrophage depletion caused by anti-CSF1R therapy^11^. However, importantly, the results obtained were derived from the scRNA-seq data from treatment-naïve CRC patients, and lack spatial information and real-world treatment-related data. Consistent with the findings of this study, our data also suggest that the C1QC^+^ RTMs promoted immune response via the regulation of MHC-II molecules which mediate antigen presentation. We found that higher C1QC^+^ RTM abundance was positively correlated with immunotherapy response and resulted in prolonged survival. Our analyses also revealed that, in responders, C1QC^+^ RTMs colocalize and interact with CD4^+^ T cells through the high expression of MHC-II molecules. Moreover, IMC analysis demonstrated that CD4^+^ T cells in closer proximity to C1QC^+^ RTMs significantly upregulated activation markers (CD38, CD57, GZMB, TNFα, and PD-1) compared to their distal counterparts, and these cells were more abundant in responders. CD4^+^ T cells play a central, multifaceted role in coordinating immune responses and are an essential component of the immune system that cannot be overlooked. Depending on the cytokine environment, TCR stimulation, and functional state of the APC, CD4^+^ T cells can differentiate into various conventional subsets, including helper T cells (Th) 1, Th17 and follicular helper T cells (Tfh)^68^. These subsets could activate and regulate other immune cells, thereby participating in host anti-tumor immunity by directly or indirectly influencing antigen presentation, co-stimulation signaling, and CD8^+^ T/NK cell activation and cytotoxicity^68–71^. Therefore, CD4^+^ T cells play an indispensable role in orchestrating precise anti-tumor immune responses. Our study demonstrates that C1QC^+^ RTMs may promote CD4^+^ T cell activation through MHC-II molecules, thereby enhancing anti-tumor immunity and benefiting immunotherapy.

Furthermore, in this study, IMC analysis revealed that non-responders exhibited a higher abundance of fibroblasts compared to responders, prompting us to further investigate the role of fibroblasts in modulating immunotherapy response. Using a ResNet18 deep learning model, we further demonstrated that fibroblasts are a critical determinant of immunotherapy outcomes. In addition, inspired by a recent study^72^, we developed a fibroblast barrier score based on IMC data to directly assess the barrier effect of CAFs on the interaction between C1QC^+^ RTMs and CD4^+^ T cells. The results showed that, regardless of microsatellite status, non-responders exhibited a higher fibroblast barrier score. Moreover, colocalization analysis of spatial transcriptomic data further demonstrated that in both MSS and MSI CRC samples, C1QC^+^ RTMs were frequently surrounded by fibroblasts in non-responders. These findings provide evidence that the physical obstruction imposed by fibroblasts on the interaction between C1QC^+^ RTMs and CD4^+^ T cells represents a key feature contributing to impaired immunotherapy response. Fibroblasts play a pivotal role in shaping the TME and modulating immune infiltration. Broz et al. revealed that fibroblasts can impede cytotoxic T-cell infiltration into the tumor parenchyma via the expression of CXCL16^73^. And Liu et al. reported that SPP1^+^ macrophages and fibroblasts combine to form a tumor immune barrier in HCC, thereby limiting immune infiltration into the tumor core^74^. On the basis of these observations, we speculate that fibroblasts may act as a barrier, hindering the anti-tumor immune capacity of C1QC^+^RTM/CD4^+^ T cell pairs.

Regorafenib can modify the TME in multiple ways, distinguishing it from other tyrosine kinase inhibitors. Studies have demonstrated that regorafenib not only inhibits the interaction between tumor cells and fibroblasts but also decreases tumor angiogenesis and lymphangiogenesis^75^. This dual action results in reduced invasion and metastasis of colon cancer. Our findings indicate that fibroblasts impede the anti-tumor immune response mediated by C1QC^+^ RTMs and CD4^+^ T cell pairs. Therefore, our study elucidated the potential mechanism of enhanced treatment efficacy of regorafenib in combination with ICIs in MSS mCRC. However, regorafenib also diminishes the population of macrophages in a dose-dependent manner by inhibiting CSF1R^76^, which may decrease the presence of C1QC^+^ RTMs, thereby limiting the utility of regorafenib. Hence, a more precise approach would involve utilizing therapeutics that specifically target fibroblasts^77^ or pro-tumorigenic TAMs in CRC treatment.

Our study has several limitations that should be acknowledged. First, the sample size may constrain the generalizability of our findings. Second, the selection of IMC ROIs to represent the TME of each patient limits our ability to fully capture intratumoral spatial heterogeneity. Third, although we employed L2 regularization and 5-fold cross-validation to mitigate overfitting, the small cohort size may still result in the ResNet18 deep learning model capturing noise rather than meaningful biological relationships. Future studies with larger sample sizes are needed to validate and extend these findings. Additionally, while our multi-omics approach provides robust evidence of co-localization and inferred interactions between C1QC⁺ RTMs and CD4⁺ T cells—particularly those mediated by HLA-DR molecules—it does not directly establish functional validation. Experimental validation of these interactions remains challenging due to the inherent complexity of reconstituting a functional MHC-II complex in humanized monocytic cell lines and primary human-derived cells.

**Fig. 8:**
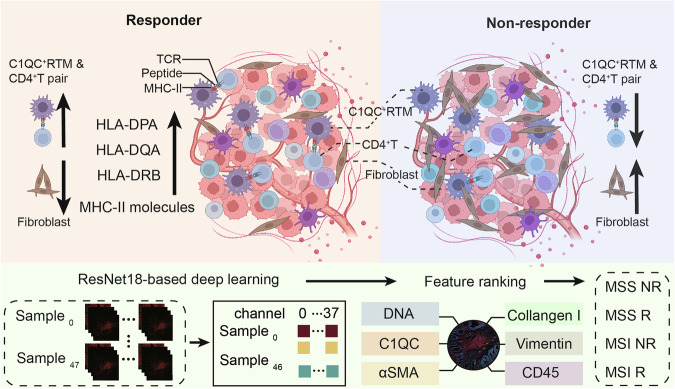
Heterogeneous TME of immunotherapy responder and non-responder CRC patients and the working model for ResNet18-based deep learning. Immunotherapy-sensitive CRC presents higher infiltration of C1QC^+^ RTM and CD4^+^ T cell pair and lower fibroblasts than immunotherapy-resistant CRC. ResNet18-based deep learning further effectively dissects the detailed spatial topology of the CRC TME and highlights the vital role of C1QC.

In conclusion, through conducting spatial multi-omics analysis and implementing deep learning techniques, we have successfully created a spatial immune atlas that pertains to CRC patients undergoing immunotherapy in real-world settings. This atlas precisely maps the multicellular ecosystem of ICIs-treated MSS/MSI CRC, revealing the involvement of C1QC^+^ RTMs in driving the immunotherapy response through enhanced MHC-II expression and interactions with CD4^+^ T cells. Our study has uncovered the importance of spatial organization and cell‒cell interactions in antitumor immunity (Fig. 8). These findings not only provide crucial insights into predicting the response to ICIs in CRC but also lay the foundation for future pan-cancer studies in a universal manner.

## Materials and methods

### Ethics Statement

The study followed the Declaration of Helsinki principles and was approved by the Medical Ethics Committee of the First Affiliated Hospital, Zhejiang University, Hangzhou, China (Approval Number: 2023-1008-EXP).

### Patient cohort

This study comprised a total of six cohorts, including three in-house cohorts and three external cohorts.

### Cohort 1 (in-house)

Cohort 1 was the primary cohort that underwent IMC and paired spatial proteomics analyses. The cohort consisted of 25 mCRC patients who underwent immunotherapy at the First Affiliated Hospital of Zhejiang University. Samples of CRC tissues were collected from all patients prior to immunotherapy, and treatment responses were assessed following immunotherapy to guide patient stratification (MSS_NR, *n* = 8; MSS_R, *n* = 8; MSI_NR, *n* = 4; MSI_R, *n* = 5). The baseline characteristics of the enrolled mCRC patients are summarized in Supplementary Table S1. The inclusion criterion for patients was pathologically confirmed stage IV CRC, with definitive MSS/MSI results tested by PCR or NGS. MSI patients received immunotherapy as first-line treatment, while MSS patients received a combination of regorafenib and immunotherapy as third-line treatment. The main exclusion criteria were patients with insufficient tissue samples or substandard quality control for further testing. For IMC and spatial proteomics analyses, two ROIs were collected from each patient, resulting in a total of 50 ROIs for each analytical platform.

### Cohort 2 (in-house)

Cohort 2 was an independent mCRC patient cohort that underwent spatial transcriptome analysis. CRC tissues were obtained before immunotherapy initiation, and patients were stratified based on their treatment responses evaluated after immunotherapy (MSS_NR, *n* = 2; MSS_R, *n* = 2; MSI_NR, *n* = 3; MSI_R, *n* = 2). The inclusion and exclusion criteria were consistent with those of Cohort 1.

### Cohort 3 (External)

Cohort 3 consisted of a publicly available cohort of patients who received neoadjuvant therapy^31^. ScRNA-seq was conducted on 27 MSI CRC tissue samples (22 pCR and 5 non-pCR) obtained from 19 patients.

### Cohort 4 (External)

In Cohort 4, a publicly available cohort^41^, RNA-seq was performed on MSI CRC tissue samples collected from 31 patients (24 pCR and 7 non-pCR).

### Cohort 5 (integrated external)

This cohort consists of seven publicly available spatial transcriptomics datasets derived from CRC tissues. The data required for analysis are accessible from the 10X Genomics website (https://www.10xgenomics.com/resources/datasets) as well as previously published studies^12,53,54^.

### Cohort 6 (in-house)

Cohort 6 included bulk proteomics data from 114 CRC patients reported in our previous study^34^. All patients were treatment-naive.

### ROI selection criteria for IMC and spatial proteomics

In both IMC and spatial proteomics analyses, two ROIs were selected from each patient, resulting in a total of 50 ROIs analyzed per platform. The ROI had an area of 1 mm² and a thickness of 4 μm. Two consecutive tissue sections were obtained for each ROI. One section was H&E stained to guide ROI selection. The other section was used for IMC analysis, a technique that ablates tissue during data acquisition. Following ROI selection, the H&E-stained section was also used for spatial proteomics analysis with an expansion-gel-based approach. This method ensured that protein-level data corresponded precisely to the IMC-analyzed regions, ensuring that the results from both techniques were directly comparable and minimizing variability caused by tumor heterogeneity.

The ROIs were selected by an experienced pathologist following a standardized protocol to ensure consistency across samples. All ROIs were chosen from tumor regions near the invasive margin, as these areas are known to be rich in immune cells and provide a representative snapshot of the tumor–immune interface. This approach allows us to analyze the dynamic interactions between enriched immune cells, which are critical for understanding immunotherapy response.

### IMC acquisition and data analysis

FFPE tissue sections were cut into two sequential 4-μm sections and heated at 68 °C for 1 h. The first section was subjected to H&E staining, and two ROIs measuring 1 mm^2^ were identified by an experienced pathologist using the same criteria. The second section was subjected to antigen retrieval via a citric acid solution, followed by washing with ddH_2_O and PBS. SuperBlock^TM^ blocking buffer was added to the tissue and incubated for 30 min at room temperature. Since lymph nodes are rich in immune cells, they provide an ideal reference for validating antibody staining and ensuring the robustness of the experimental protocol. We used normal, non-metastatic lymph node tissue from patients independent of the IMC cohort as a positive control to evaluate the staining performance of the IMC panel. After confirming the staining quality, the same IMC staining protocol was applied to CRC tissues. A 50 µL antibody mixture was added to each tissue and incubated overnight at 4 °C. The samples were then stained with Iridium solution and dried before being stored at 4 °C for detection by the Hyperion Imaging System. The data analysis included spillover signal compensation, image denoising, image contrast enhancement, and cell segmentation based on a previous study^78^. The image channel was segmented into individual cells and components via connection-sensing segmentation. The regionprops function in MATLAB was utilized to identify connected components for cell segmentation. Artifacts in other membrane channels were eliminated if their distance to the nearest nuclei centroid exceeded 15 pixels. Marker expression was normalized, and batch effects were corrected via the “Harmony” package. Cell clustering was performed via the “Rphenograph” package with 100 nearest neighbors, and the resulting cluster means were visualized using heatmaps. The “imcRtools” package was employed for downstream analysis. To determine the CN of each cell, the 20 nearest neighboring cells were identified based on Euclidean distance. To identify the optimal number of CNs, we systematically tested different values of k (the number of clusters) and evaluated their impact on the clustering results. Specifically, we tested k = 5, 10, 15, and 20, ultimately selecting k = 15, as it provided the best balance between granularity and biological interpretability. A logistic regression model based on cell frequency (cell count/total nuclei count) was used to evaluate the association between cell abundance and immunotherapy response, implemented via the “glm” function in the “stats” package in R, as previously described by Wang et al. ^79^ The use of cell frequency allows for biologically meaningful interpretation of immune cell infiltration patterns and ensures methodological consistency with Ro/e analysis, MiloR differential abundance testing, and odds ratio calculations.

### The definition of RTMs

RTMs are defined by a set of characteristic markers, including C1QC, C1QA, CD163, FOLR2, and APOE as supported by previous literature^80–82^. In the IMC data, due to the limited number of antibodies available in the panel, we primarily used C1QC to define RTMs. C1QC^+^ RTMs exhibited high expression of C1QC and CD163, while CD163^*−*^C1QC^median^ RTMs display moderate expression of C1QC, both of which were defined as resident tissue phenotypes. In scRNA-seq, C1QC^+^ RTMs highly expressed C1QC, C1QA, CD163, and FOLR2, further indicating the tissue-resident characteristics of this subset. SPP1^+^C1QC^median^ RTMs (which share a similar phenotype with CD163^*−*^C1QC^median^ RTMs identified in IMC) exhibited high expression of APOE and moderate expression of C1QC, thus also classified as RTMs.

### t-SNE visualization

t-SNE was used to embed each category (MSS_NR/MSS _R/MSI_NR/MSI_R) in a 2D plot. We defined a label array “labels” where each category has a corresponding numerical identifier. Then, we used sklearn.manifold.TSNE to reduce the dimensionality of the high-dimensional feature vector all_sample_vec to a two-dimensional space, resulting in features_embedded. Finally, we plotted the reduced data features_embedded and assigned a different color for each category.

### Hierarchical clustering dendrogram

A dendrogram displaying the hierarchical clustering results of the feature vectors was created on the basis of the feature vector array all_sample_vec. We performed hierarchical clustering analysis on all_sample_vec using the scipy.cluster.hierarchy.linkage function with the Ward’s method to calculate the distance between clusters. The Ward’s method aims to minimize the total sum of squared errors within clusters, where the sum of squared errors within a cluster refers to the sum of the squared distances of all samples to the cluster center. The linkage function returns a matrix Z that contains information about all the clustering steps. We passed the matrix Z to the scipy.cluster.hierarchy.dendrogram function to plot the dendrogram of the hierarchical clustering.

### Tissue distribution of clusters

For each cell subtype, we evaluated its distribution pattern across the four groups through the calculation of the Ro/e as previously described^26,32,83–85^. Specifically, Ro/e is the ratio of the observed cell number to the expected cell number of a given combination of cell subtype and tissue. We used the “calTissueDist” function from the Startrac package to obtain the observed and expected cell numbers, using cell frequency as the input data. Briefly, a cell subtype was more frequently observed in a specific tissue than random expectations when Ro/e > 1 and was therefore assumed to be enriched in that group.

### Calculation of sample similarity

In our study, we evaluate the similarity between samples by computing the cosine similarity of their features. Specifically, we utilized data from 37 channels in each sample to train a classification model, denoted as M, based on the ResNet18 architecture. During the training process, we applied L2 regularization (with a weight decay parameter of 0.01) to all fully connected layers except the output layer to avoid overfitting and improve the model’s generalizability. Additionally, 5-fold cross-validation was employed to assess the model’s performance across different subsets of the data, ensuring its robustness and preventing overfitting by evaluating on multiple data splits.

Subsequently, we employed model M to transform the 37 channels of each sample into a 1D vector and calculated the cosine similarity between the vectors derived from different samples. A cosine similarity value approaching 1 indicates higher similarity between the two vectors, whereas a value nearing -1 suggests lower similarity. This process enables us to quantitatively assess the resemblance between samples based on their feature representations utilizing our classification model. The cosine similarity is calculated as follows:

$$\cos ({\vec{a}}{{\cdot }}{\vec{b}})=\frac{{\vec{a}}{{\cdot }}{\vec{b}}}{\left|{\vec{a}}\right|\left|{\vec{b}}\right|}$$

where the numerator is the inner product of two vectors and the denominator is the product of the modules (lengths) of two vectors.

### Calculation of channel importance

We employed Permutation Importance to conduct channel importance analysis. Permutation Importance is a model-agnostic variable filtering method that can be applied to any model without necessitating retraining. Initially, an input variable (channel data) is randomly shuffled, and the model is subsequently evaluated on the validation set to assess the impact on model output. A decrease in model performance indicates the variable’s significance in accurately predicting outcomes, with greater decrements reflecting higher importance of the variable in the model’s predictive capability.

Specifically, the initial step involves scrambling the data from channel x in each sample, followed by inputting model M to achieve classification results. By evaluating the disparity in classification accuracy pre- and post-scrambling, the significance level of channel x is determined. To enhance the reliability of our findings and minimize the impact of chance variation, we conducted three independent random scrambles of each channel’s data. 5-fold cross-validation was also employed to ensure that the importance results remained stable across different data splits, thus reducing the risk of overfitting to a single data subset.

### Barrier score construction

The fibroblast barrier score was calculated using the method described in Failmezger et al.’s study^86^. First, the nearest neighbor map of cell location was constructed by connecting each cell with its five nearest neighbors. To calculate the fibroblast barrier of CD4^+^ T cells relative to C1QC^+^ RTMs, we used the Python “cuGraph” search shortest path algorithm to find the shortest path for each CD4^+^ T cell to C1QC^+^ RTM. Fibroblasts were then enumerated along each path. For CD4^+^ T cells of origin with multiple C1QC^+^ RTMs at the same distance, the score was defined as the mean number of fibroblasts along all paths. To limit the score to fibroblasts that clustered at the edge of the C1QC^+^ RTMs, we counted only fibroblasts in the vicinity of the C1QC^+^ RTMs. Barrier scores were calculated as the average of all CD4^+^ T cells per image, with a barrier score of 1 (otherwise 0) for CD4^+^ T cells isolated from fibroblasts adjacent to the nearest C1QC^+^ RTM, followed by an average score for all shortest paths for each CD4^+^ T cell to the nearest C1QC^+^ RTM.

### scRNA-seq and bulk RNA-seq data analysis

The scRNA-seq data was analyzed using the Seurat package in R software with default parameters^87^. UMAP, implemented in R software, was used for dimensional reduction. Highly expressed genes within each cluster were identified using the Seurat package, with an adjusted *P* value threshold of less than 0.05. Major clusters were identified using a clustering algorithm based on shared nearest neighbors (SNNs) and modularity optimization. The “FindNeighbors” and “FindClusters” functions with default settings were used. Further clustering analysis was conducted to identify subsets within the major cell clusters. The Benjamin-Hochberg adjustment method was used to account for multiple tests. The “clusterProfiler” package was used to perform GO analysis on the identified genes. The GSVA package in R software was used to calculate the single-sample gene set enrichment analysis (ssGSEA) score for each gene set^88^. The Fast GSEA (FGSEA) was performed with the “fgsea” package. The “cellchat” package in R software was used to explore ligands and target gene pairs involved in cell-cell interactions^89^.

### Spatial proteomics acquisition and data analysis

The workflow of spatial proteomics was similar to that of our previous work^43^ with some modifications. The H&E stained sections from the IMC were used for microscopic imaging and ROI confirmation. A new continuous section was taken and incubated with a protein anchoring solution. After incubation, the reaction was stopped with an anchor stop solution. The tissue was air-dried in a fume hood and coated with a gelation solution. It was then incubated overnight in a refrigerator. The cover glass was placed on top and put into a vacuum drying oven at 37 °C to form a hydrogel in an oxygen-free environment. Next, the cover glass was removed, and a denaturation solution was added to the sample. Physical denaturation treatment was performed using a sterilization pot. The sample was washed three times with 1× PBS (pH 7.4). Coomassie Brilliant Blue staining solution was applied, followed by quick washing with 1× PBS (pH 7.4). The sample was expanded to achieve the desired expansion factor. The location of interest was selected, and the sample was transferred to a tip device. Further steps included decolorization, dehydration, reduction, alkylation, and overnight trypsin digestion, followed by sequential extraction and recovery of peptides from low to high organic phases. The samples were dried and redissolved using a mass spectrometry buffer. The redissolved samples were subjected to a 60-min linear LC gradient of diaPASEF mass spectrometry detection using timsTOF Pro. Finally, DIA-NN (version 1.8.1) software^90^ was employed to search the acquired mass spectrometry files in a self-built laboratory library and obtain the final protein matrix for subsequent data analysis.

### Genome scale CRIPSR screen in THP-1 cells

#### Cell culture

THP-1 cells (ATCC) were cultured in RPMI 1640 media + 10% FBS (Sigma‒Aldrich) + 100 U Pen/Strep and maintained at 37 °C and 5% CO_2_ in a humidified tissue culture incubator.

#### gRNA pool library production

Human CRISPR Brunello lentiviral pooled library (Addgene, Plasmid #73138) consisting of 76,441 gRNAs was co-transfected with packaging plasmids (psPAX2 and pMD2.G) into HEK293T cells using LipoD 293T transfection reagent (Signagen, #SL100668) following the manufacturer’s protocol. Twenty-four hours after transfection the media was replaced. The virus supernatant was collected at 48 and 72 h after transfection. The virus was concentrated using PEG virus precipitation (Promega, #V3011). In brief, the collected supernatant was pooled and spun down at 3000× *g* for 15 min to remove cell debris. The supernatant was carefully collected, and 8 mL of 40% PEG8000 solution was added to 32 mL of viral supernatant for a final concentration of 8% PEG8000. After mixing well with vortexing the virus supernatant was incubated overnight at 4 °C. The next day, the viral supernatant was centrifuged at 3000× *g* for 30 min at 4 °C. The supernatant was aspirated carefully to avoid disturbing the virus pellet which was then resuspended in RPMI and stored at –80 °C. To determine the virus titer, 1 × 10^6^ THP1-Cas9 cells were plated per well of a 24-well plate. THP1-Cas9 cells were transduced with different amounts of the aliquoted lentivirus in the presence of 8 µg/mL of polybrene. Twenty-four hours following infection, puromycin (2 µg/mL) was added. After 3 days of puromycin selection, infected cells in each well were counted to determine the virus efficiency.

#### Screening

THP1-Cas9 cells were transduced with the Brun86ello library lentivirus at an MOI of 0.3, and the cells were selected with puromycin (2 µg/mL) prior to PMA priming and following polarization. All the differentiation conditions involved the addition of PMA (Sigma-Aldrich) to RPMI media with 10% FBS for 24 h to prime THP-1 monocytes into macrophage-like cells. After washing off PMA, cells were rested in fresh media for 24 h before exposure to polarizing cytokines (IFN-γ + LPS). For M1 cells, the IFN-γ and LPS concentrations were maintained consistently during the 48-h cytokine exposure.

#### Library preparation and sequencing

Genomic DNA was isolated using the DNeasy Blood and Tissue Kit (Qiagen, #51192) following the manufacturer’s protocol. PCR was conducted using Q5 Hot Start High-Fidelity 2× Master Mix, with an input gDNA amount of 15 µg for each sample. The thermal cycling conditions included an initial denaturation step at 98 °C for 3 min followed by 28 cycles of amplification (10 s at 98 °C, 30 s at 60 °C, and 25 s at 72 °C) and a final extension step at 72 °C for 5 min. PCR amplification of the gRNA cassette for Illumina sequencing of the gRNA representation was done following the Broad protocol available online (https://media.addgene.org/cms/filer_public/56/71/5671c68a-1463-4ec8-9db5-761fae99265d/broadgpp-pdna-library-amplification.pdf). NGS Illumina sequencing was done by the YCGA core to a depth of 200*×*.

#### Screen data analysis

Raw sequencing fastq data had adapter sequences trimmed via Cutadapt v3.41^91^ using a 10% error rate and the following sequences: forward, 5ʹ-tcttgtggaaaggacgaaacaccg; reverse, 5ʹ-gttttagagctagaaatagcaagt. Trimmed sequences were then filtered to remove those with < 15 nt length. The remaining sequences were aligned to a reference, comprising the CRISPR sgRNA-spacer sequences. Alignment was performed using Bowtie v1.3.02 with the following settings: -v0, -m1 -best. The sgRNA counts for each sample were processed and analyzed using SAMBA R package v1.3.0 (https://github.com/Prenauer/SAMBA). Specifically, sgRNAs were filtered to include those with > 10 counts across screened samples (non-control). A two-step data analysis was performed, first with an sgRNA-level analysis by the edgeR R package v3.38.43 pipeline with TMM-wsp size factors, feature-wise dispersion, quasilikelihood (QL) generalized linear model fitting, and QL F tests. In the second analysis step, sgRNA scores were aggregated into a gene score, calculated as a weighted sum of the sgRNA log2 fold-changes (log2FC). Gene level *P* values were assessed based on a null distribution of gene scores, which were scored from randomly grouped sgRNAs of non-targeting controls. *P* values were adjusted using the method by Benjamini and Hochberg. An additional metric to assess gene enrichment was the number of sgRNA/gene with a log2FC > the 90th percentile of the randomized null data log2FC, representing a 10% FDR. Screen data were also analyzed with the commonly used MAGeCK RRA algorithm for robust comparison^92^. We assessed the effects of MHCII^high^ and MHCII^low^ coefficients and verified that there was high gRNA detection for all samples.

#### Flow cytometry

During the polarization of *ESRRA*-KO THP-1 and corresponding control cells (THP-1-Cas9, Vector control) using IFN-γ + LPS, induced cells were collected on days 1, 2 and 3 to access the dynamic changes in MHC-II expression. Harvested cells from each group were washed with pre-cooled FACS buffer (PBS containing 2% FBS) and the cell pellets were resuspended for subsequent FcR blocking using truStain FcX (BioLegend). Subsequently, staining antibodies were added at optimal concentrations and incubated for 30 min at 4 °C in the dark. The antibodies used for flow cytometry were listed as follows: anti-human CD11b FITC (ICRF44), anti-human CD80 BV421 (2D10) and anti-human HLA-DR, DP, and DQ PE (Tü39). For live/dead discrimination, LIVE/DEAD Fixable NIR commercial kit (Invitrogen) was employed. Prepared samples were run on a BD FACSAria II flow cytometer (BD Biosciences). For intracellular staining, the cells were fixed and permeabilized using the Intracellular Fixation & Permeabilization Buffer Set (Invitrogen) according to the manufacturer’s protocol. The following antibodies were used: rabbit anti-human C1QC polyclonal antibody and FITC-conjugated goat-anti-rabbit secondary antibody. For cell sorting, MHC^high^ and MHC^low^C1QC^+^ THP-1 cells were sorted with a BD FACSAria II flow cytometer (BD Biosciences) for library readout. Data analysis and visualization were performed via FlowJo software (v10.8.1).

### Spatial transcriptomics acquisition and data analysis

Nine CRC FFPE tissues were cut into small pieces (4–5 mm^3^) and fixed and embedded in paraffin. The target area of the tissue was sliced using a Lycra microtome following the 10*×* Genomics Protocol (CG000518, RevB). To prepare the FFPE samples, dewaxing, H&E staining, and decrosslinking steps were carried out according to the 10*×* Genomics Visium CytAssist Spatial Gene Expression for FFPE instructions (CG000520, RevA). The probe hybridization and library construction were performed following the product description of the 10*×* Genomics Visium CytAssist Spatial Gene Expression Reagent Kit (CG000495, Rev C). The constructed library was sequenced using Illumina NovaSeq 6000 or BGI-T7, with a PE-150 sequencing method. The raw sequencing readings were quality-checked with Space Ranger-2.0.0, followed by the generation of expression matrices and spot location information. For the in-house cohort, the RCTD deconvolution method was applied to calculate the proportions of various cell types in each spot. Based on the spot-cell type composition matrix, the cellular niche of each tissue slice was identified. The MSITy algorithm was used for co-localization analysis, which calculated the dependencies between cell types within a spot. The gene set scoring was based on the AddModuleScore algorithm. For the external cohort, the SCTransform function in the Seurat (v4.0) was used to normalize the data. Dimensional reduction and unsupervised clustering were implemented with independent component analysis (dims = 1: 30). The clusters were annotated according to the expression level of classical markers and the corresponding slice morphology. Spatial communication analysis was performed with the “CellChat (v2)” package^55^.

### mIF staining

After deparaffinization of paraffin sections (4–5 μm), antigen repair was performed in microwave (100% power for 5 min, followed by 60% power for 15 min). The sections were then blocked using blocking solution for 30 min at room temperature. The primary antibody was incubated for 1–2 h at room temperature, followed by incubation with the appropriate secondary antibody for 30 min. The cells were incubated with TSA dye and DAPI nuclear staining (TissueGnostics, Vienna, Austria) for 10 min and then washed with PBS. This procedure was repeated for each antibody. The spatial relationships between immune cells were evaluated using infiltration analysis. The HALO™ system was used to visually compare location patterns within the immune cells and measure cell-to-cell distances.

### Statistical analysis

The statistical visualizations were created using PRISM (version 10.0.3) and R (version 4.0.4). The univariate Cox test was utilized to compare the survival outcomes.

The data were presented as mean values with standard error of the mean (SEMs). A significance level of *P* < 0.05 or adjusted *P* < 0.05 was used to determine statistical significance. The following symbols were used to indicate the level of significance: *P < 0.05, **P < 0.01, ***P < 0.001, and ****P < 0.001.

## Supplementary information

Supplementary Information

## Data Availability

Raw sequencing data generated in this study are deposited in Genome Sequence Archive for Human (https://ngdc.cncb.ac.cn/gsa-human/) with accession number OMIX005760 and HRA006753. This paper does not report original code.
