# Supplementary material for: Spatially resolved C1QC+ macrophage-CD4+ T cell niche in colorectal cancer microenvironment: implications for immunotherapy response
Source: Cell Discov. 2025 Jul 1;11:60. doi: 10.1038/s41421-025-00811-2 (PMC12219098; doi:10.1038/s41421-025-00811-2)
Supplement: Supplementary file 1 — Supplementary Information [file 41421_2025_811_MOESM1_ESM.pdf]

Supplementary Materials for

Spatially Resolved C1QC$^+$ Macrophage-CD4$^+$ T Cell Niche in Colorectal Cancer Microenvironment: Implications for Immunotherapy Response

Hangyu Zhang et al.

*Corresponding author. Email: xuanwen.bao@zju.edu.cn (XB), weijiafang@zju.edu.cn (WF), guotiannan@westlake.edu.cn (TG).

This file includes:

Supplementary Figs. S1 to S27

Supplementary Tables S1 to S9

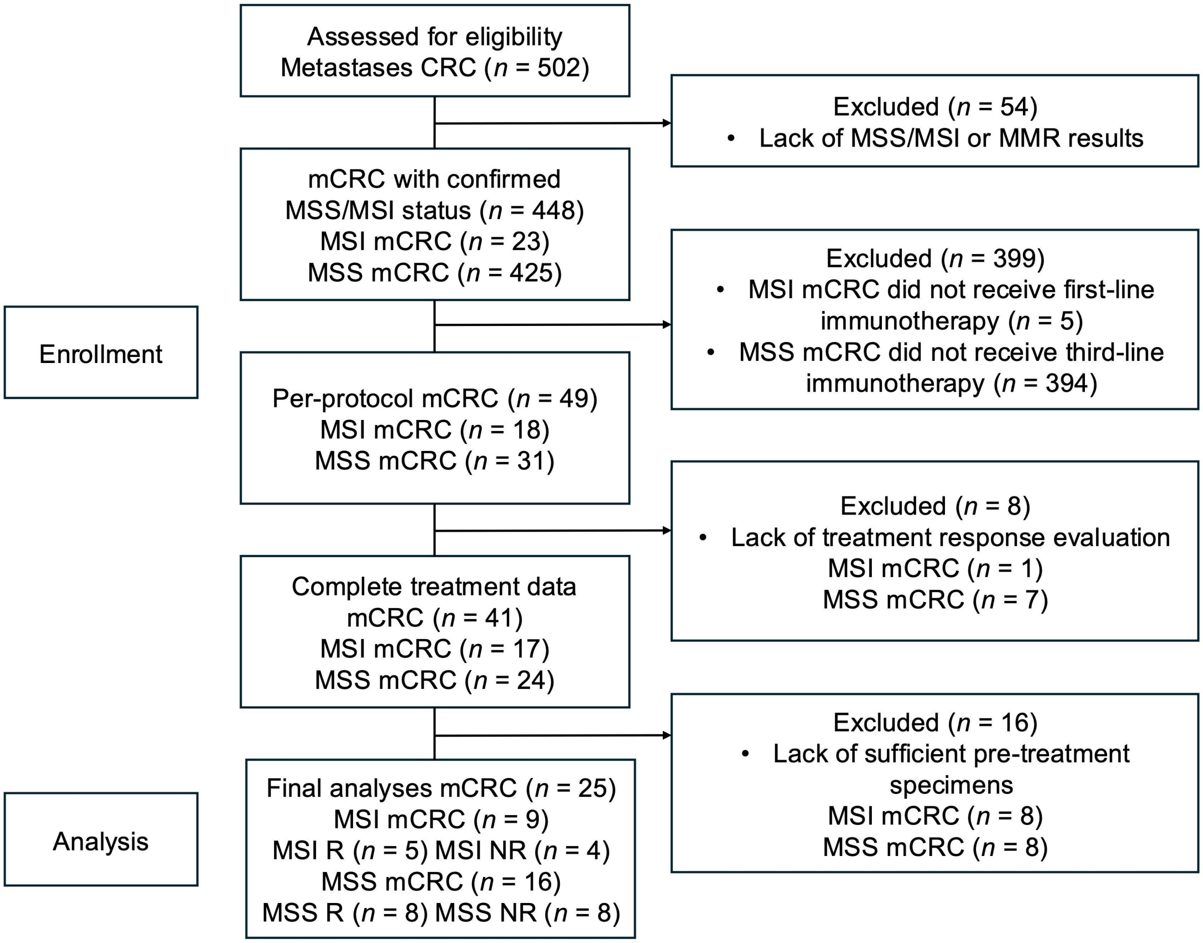

Supplementary Fig. S1. The CONSORT flow diagram of this study. Among 502 mCRC patients, 16 MSS and 9 MSI cases were selected for analysis.

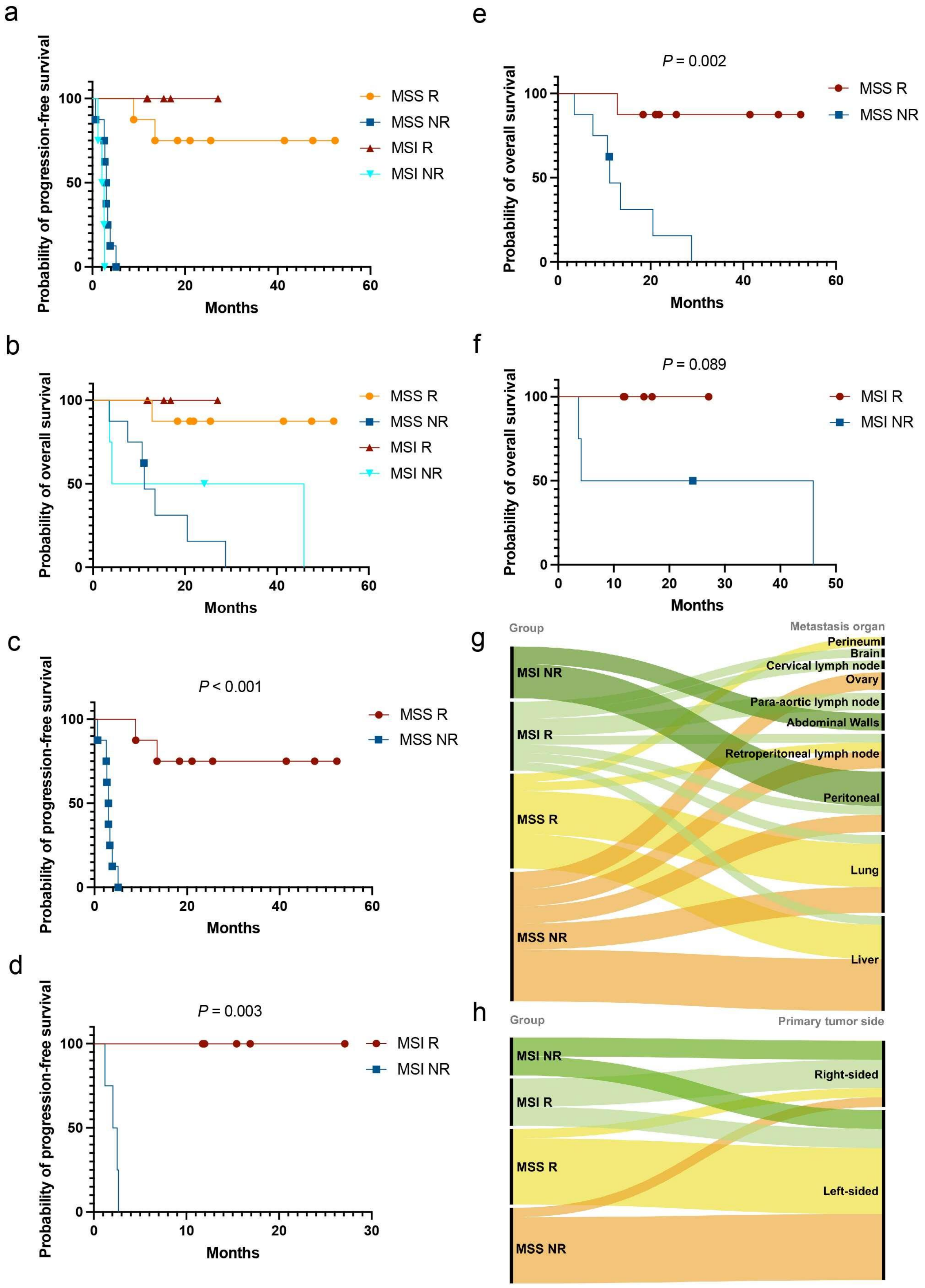

Supplementary Fig. S2. Clinical outcomes and characteristics of enrolled 25 CRC

patients. (a) Progression free survival (PFS) in all four subtypes of CRC groups. (b) Overall survival (OS) in all four subtypes of CRC groups. (c) PFS according to R and NR subgroup in MSS cohort. (d) PFS according to R and NR subgroup in MSI cohort. (e) OS according to the R and NR subgroup in the MSS cohort. (f) OS according to R and NR subgroup in MSI cohort. The Log-Rank Test were adopted to evaluate the statistical significance. (g) Metastasis sites of all enrolled patients. (h) Primary tumor sites of all participants. The log-rank method was adopted to evaluate the statistical significance.

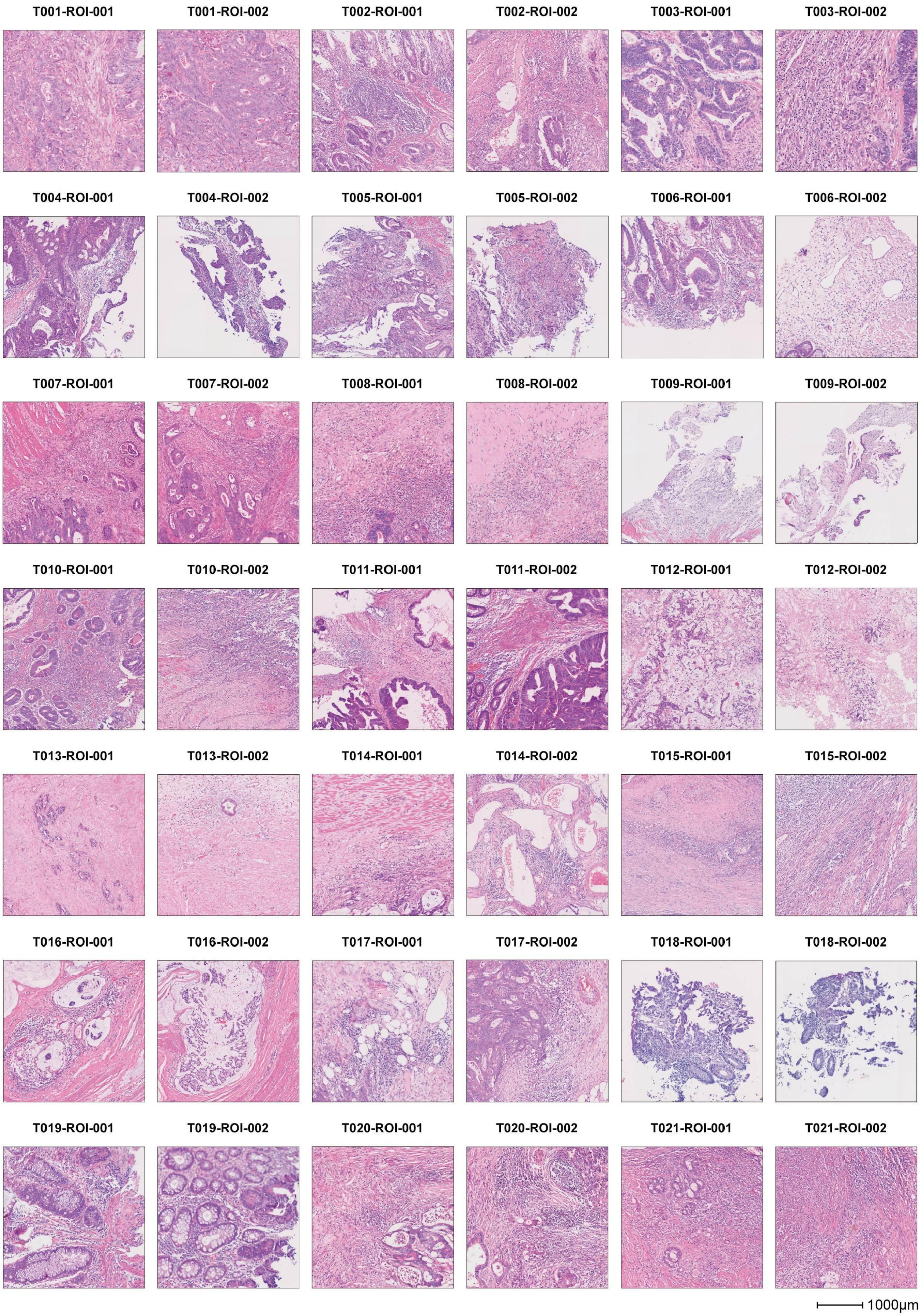

Supplementary Fig. S3. HE staining across ROIs from patients T001 to T021.

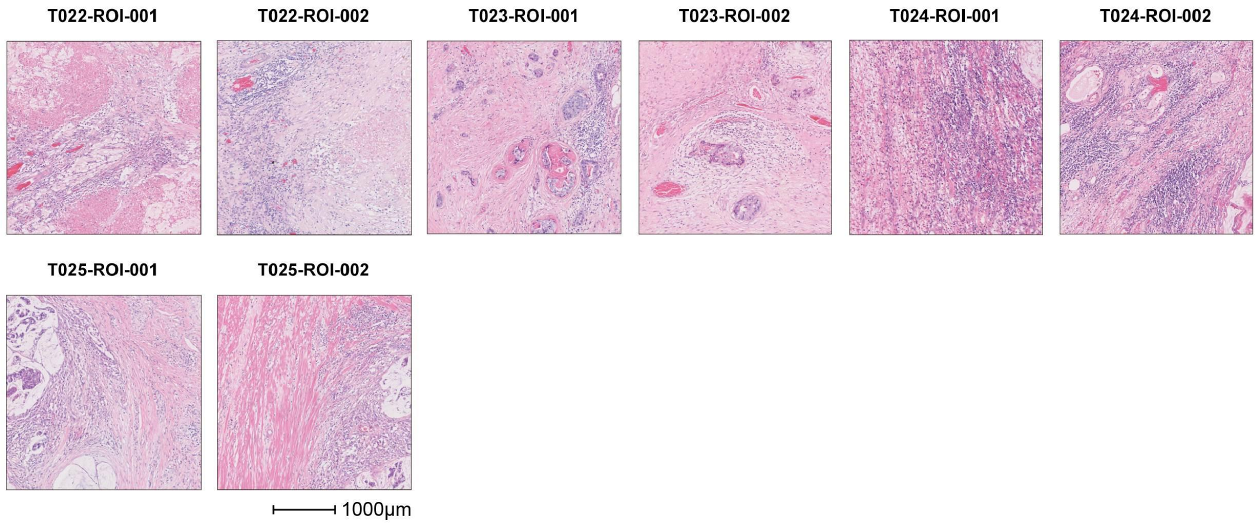

Supplementary Fig. S4. HE staining across ROIs from patients T022 to T025.

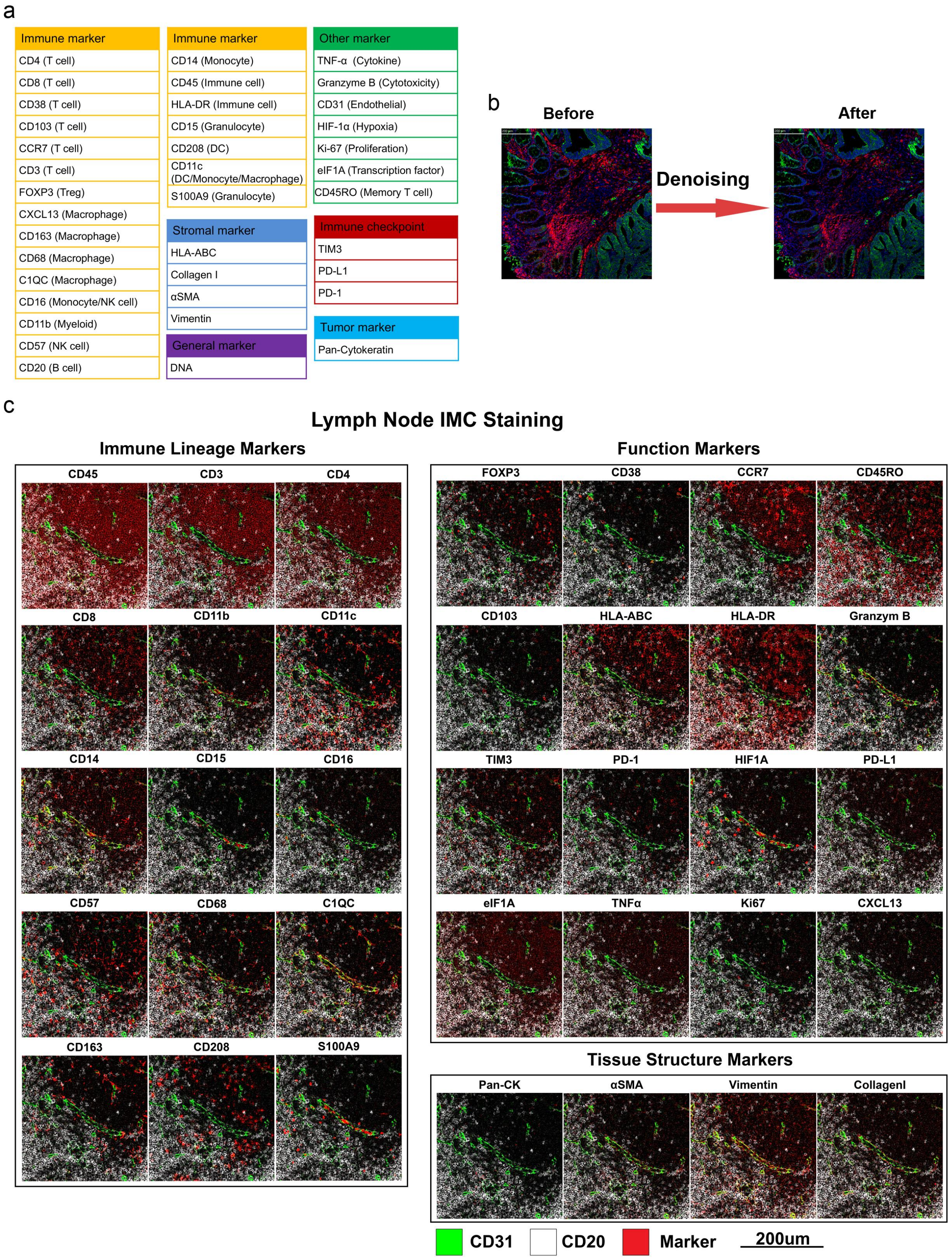

Supplementary Fig. S5. IMC marker panel and representative images of each marker on lymph node. (a) Panel of all the markers used in the IMC. (b) Raw IMC image and processed image example of HLDR staining. (c) CD31 (green), and CD20

(white) were used to portray the structure of lymph node, stained with the indicated marker (red) above each plot.

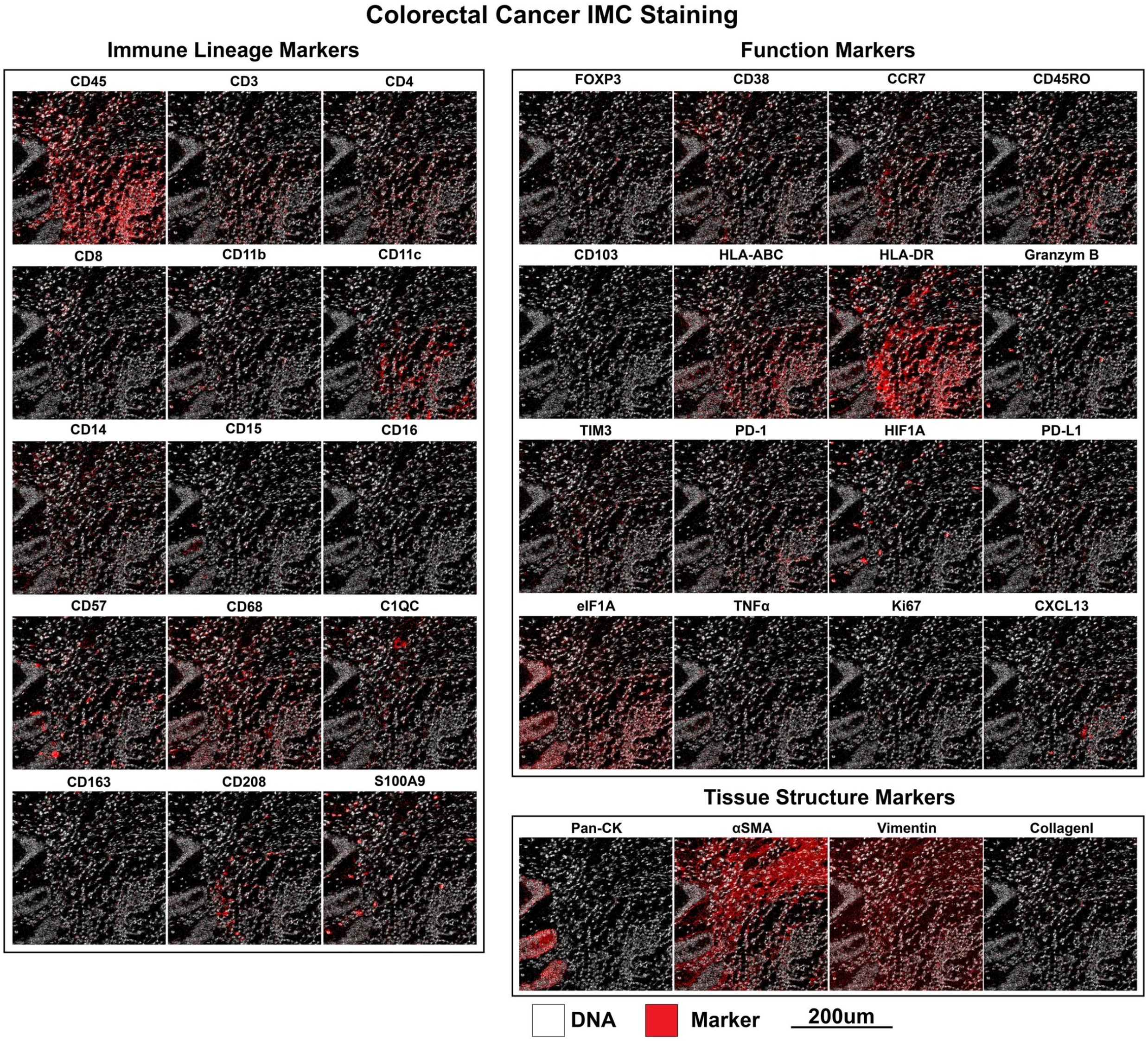

Supplementary Fig. S6. Images of each marker on one ROI from CRC tissues. Representative images of marker expression (marker (red) and DNA (white)).

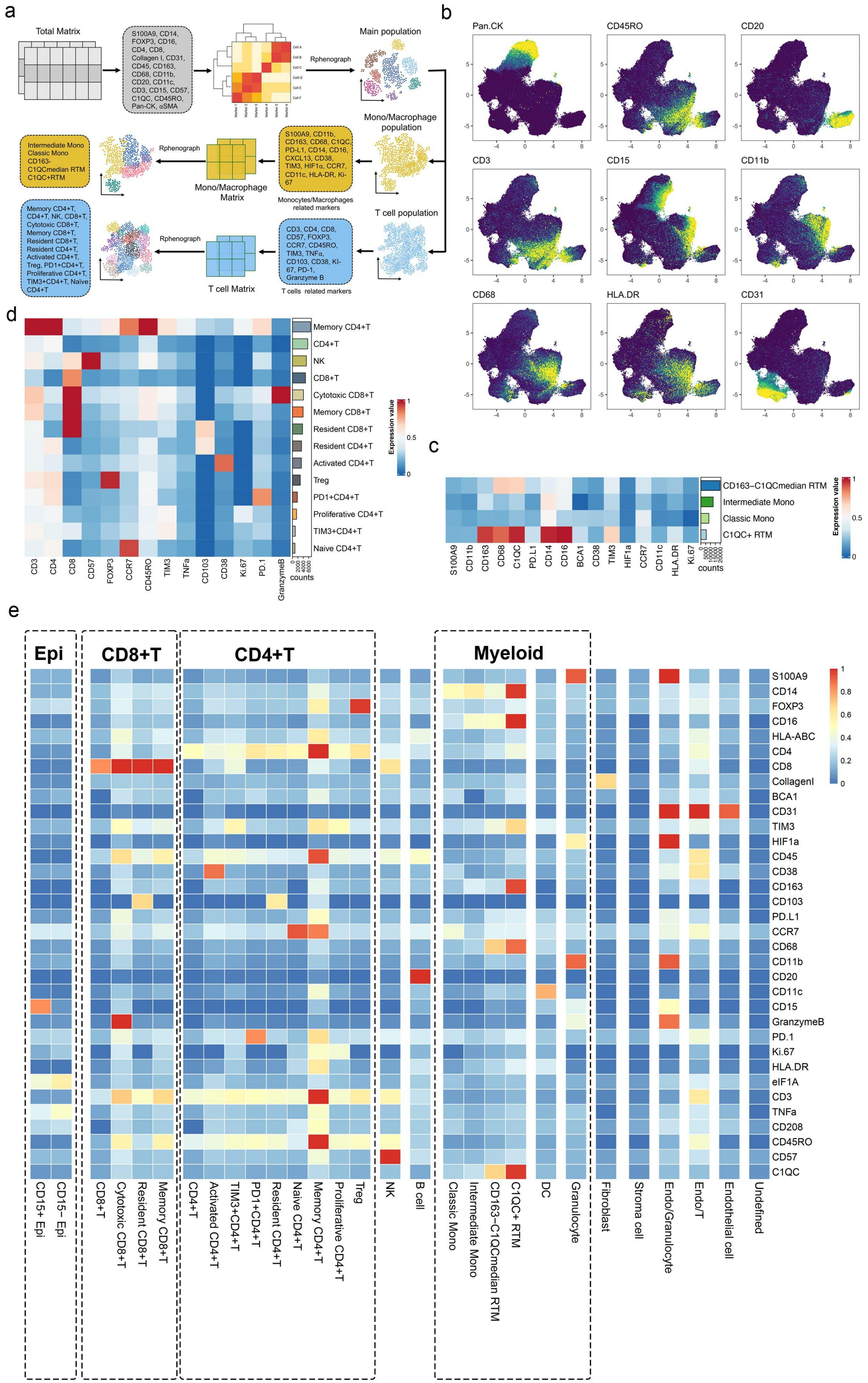
a
Total Matrix
S100A9, CD14, FOXP3, CD16, CD4, CD8, Collagen I, CD31, CD45, CD163, CD68, CD11b, CD20, CD11c, CD3, CD15, CD57, C1QC, CD45RO, Pan-CK, αSMA
Rphenograph
Main population
Mono/Macrophage population
Intermediate Mono
Classic Mono
CD163-C1QCmedian RTM
C1QC+RTM
Mono/Macrophage Matrix
S100A9, CD11b, CD163, CD68, C1QC, PD-L1, CD14, CD16, CXCL13, CD38, TIM3, HIF1α, CCR7, CD11c, HLA-DR, Ki-67
Monocytes/Macrophages related markers
T cell population
Memory CD4+T, CD4+T, NK, CD8+T, Cytotoxic CD8+T, Memory CD8+T, Resident CD8+T, Resident CD4+T, Activated CD4+T, Treg, PD1+CD4+T, Proliferative CD4+T, TIM3+CD4+T, Naive CD4+T
T cell Matrix
CD3, CD4, CD8, CD57, FOXP3, CCR7, CD45RO, TIM3, TNFα, CD103, CD38, KI-67, PD-1, Granzyme B
T cells related markers
b
Pan.CK
CD45RO
CD20
CD3
CD15
CD11b
CD68
HLA.DR
CD31
c
CD163−C1QCmedian RTM
Intermediate Mono
Classic Mono
C1QC+ RTM
S100A9
CD11b
CD163
CD68
C1QC
PD.L1
CD14
CD16
BCA1
CD38
TIM3
HIF1a
CCR7
CD11c
HLA.DR
Ki.67
counts
Expression value
d
Memory CD4+T
CD4+T
NK
CD8+T
Cytotoxic CD8+T
Memory CD8+T
Resident CD8+T
Resident CD4+T
Activated CD4+T
Treg
PD1+CD4+T
Proliferative CD4+T
TIM3+CD4+T
Naive CD4+T
CD3
CD4
CD8
CD57
FOXP3
CCR7
CD45RO
TIM3
TNFa
CD103
CD38
Ki.67
PD.1
GranzymeB
counts
Expression value
e
Epi
CD8+T
CD4+T
Myeloid
S100A9
CD14
FOXP3
CD16
HLA-ABC
CD4
CD8
CollagenI
BCA1
CD31
TIM3
HIF1a
CD45
CD38
CD163
CD103
PD.L1
CCR7
CD68
CD11b
CD20
CD11c
CD15
GranzymeB
PD.1
Ki.67
HLA.DR
eIF1A
CD3
TNFa
CD208
CD45RO
CD57
C1QC
CD15+ Epi
CD15− Epi
CD8+T
Cytotoxic CD8+T
Resident CD8+T
Memory CD8+T
CD4+T
Activated CD4+T
TIM3+CD4+T
PD1+CD4+T
Resident CD4+T
Naive CD4+T
Memory CD4+T
Proliferative CD4+T
Treg
NK
B cell
Classic Mono
Intermediate Mono
CD163−C1QCmedian RTM
C1QC+ RTM
DC
Granulocyte
Fibroblast
Stroma cell
Endo/Granulocyte
Endo/T
Endothelial cell
Undefined

Supplementary Fig. S7. Characterization of immune cells in CRC TME. (a) The workflow for identifying main cell populations and monocyte/macrophage subpopulations. (b) Representative immune markers shown on Uniform Manifold Approximation and Projection (UMAP). (c) Heatmap showing expression patterns of monocyte/macrophage-related markers across indicated macrophage and monocyte cell clusters. (d) Heatmap showing expression patterns of T cell-related markers across indicated NK and T cell clusters. (e) Heatmap showing expression patterns of all markers across all clusters.

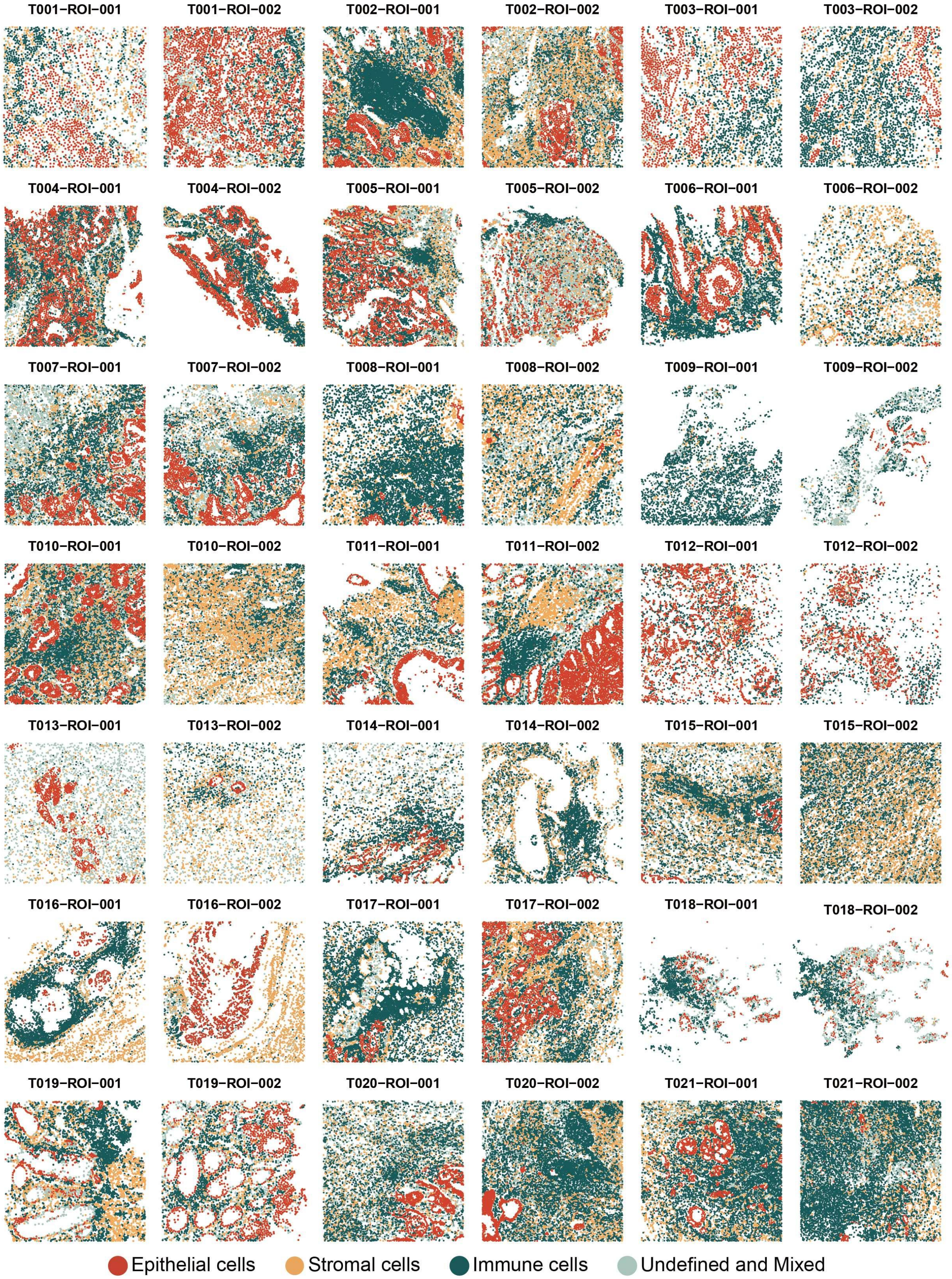

Supplementary Fig. S8. Spatial cell map across ROIs from patients T001 to T021. Spatial maps illustrating the distribution of major cell types (epithelial cells, immune cells, and stromal cells) within each ROI.

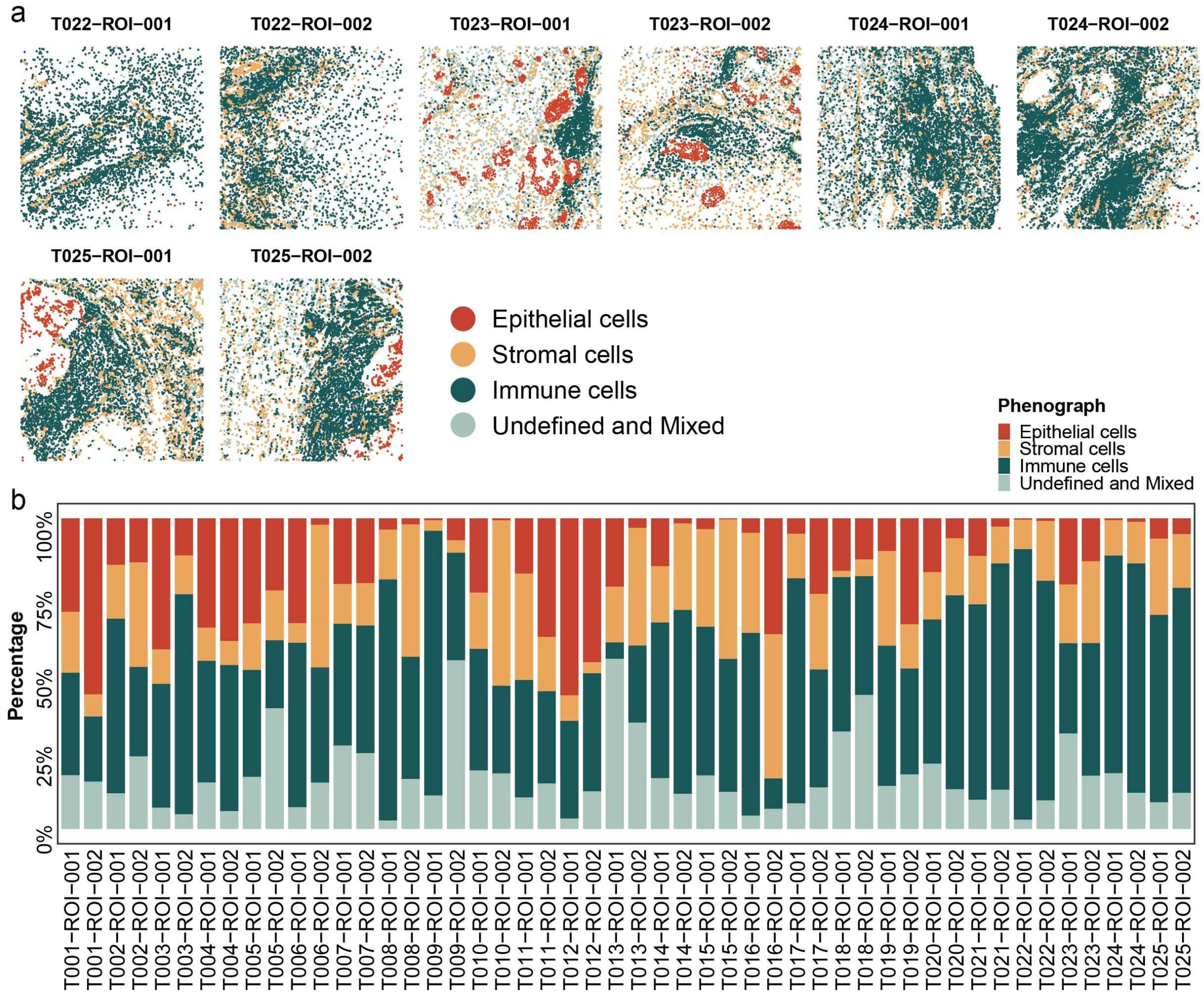

Supplementary Fig. S9. Spatial cell distribution and composition analysis. (a) Spatial cell maps of ROIs from patients T022 to T025, illustrating the distribution of major cell types within the tumor microenvironment. (b) Quantitative composition analysis of ROIs from patients T021 to T025, showing the relative proportions of immune, stromal, and epithelial cell populations.

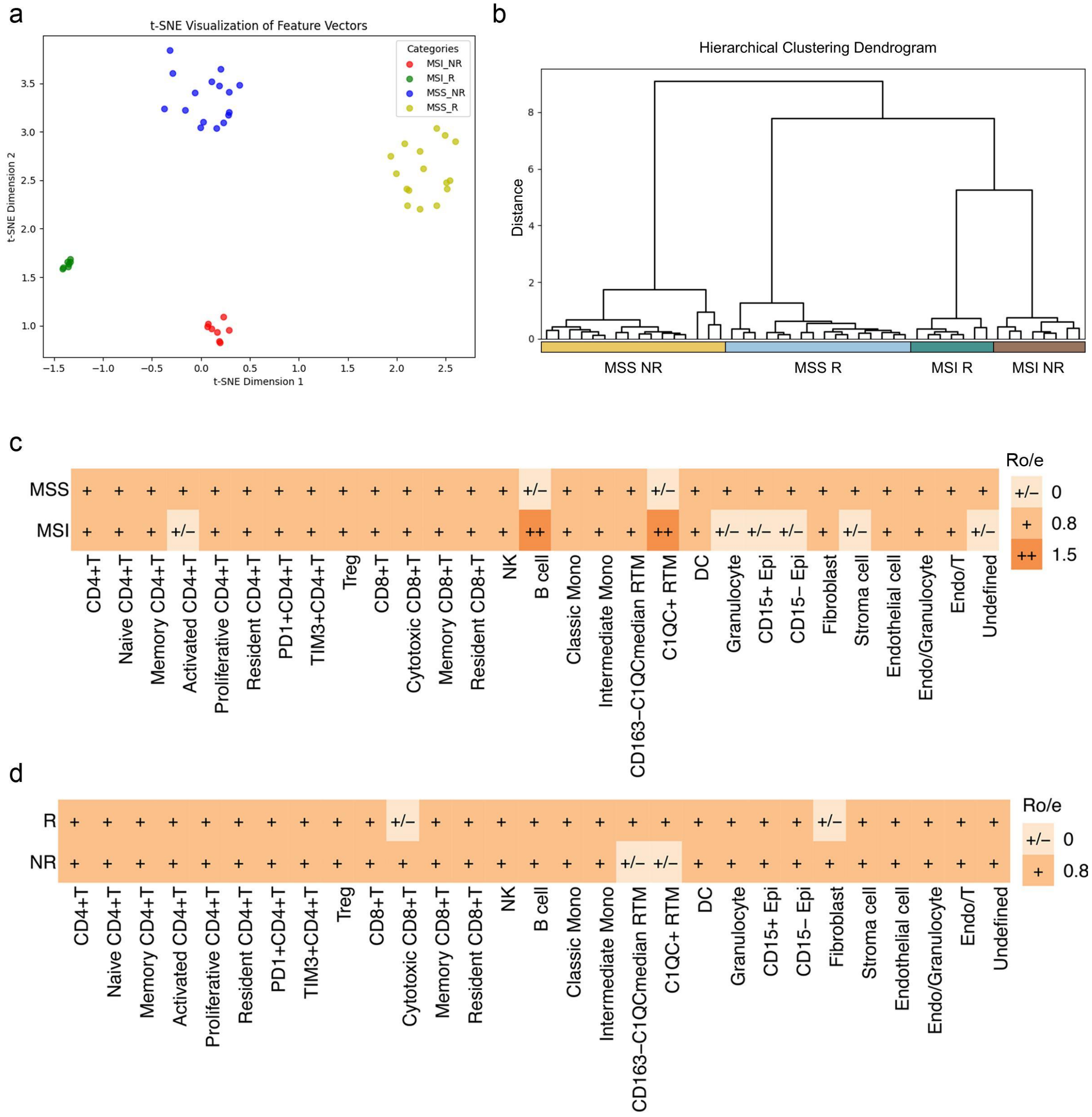

Supplementary Fig. S10. Infiltration density of cell subcluster in MSS/MSI R and NR group. (a) The t-SNE of IMC samples in this study. (b) The hierarchical clustering dendrogram of IMC samples in this study. (c) Ro/e analysis of immune cells infiltrated in MSS and MSI group. (d) Ro/e analysis of immune cells infiltrated in R and NR group.

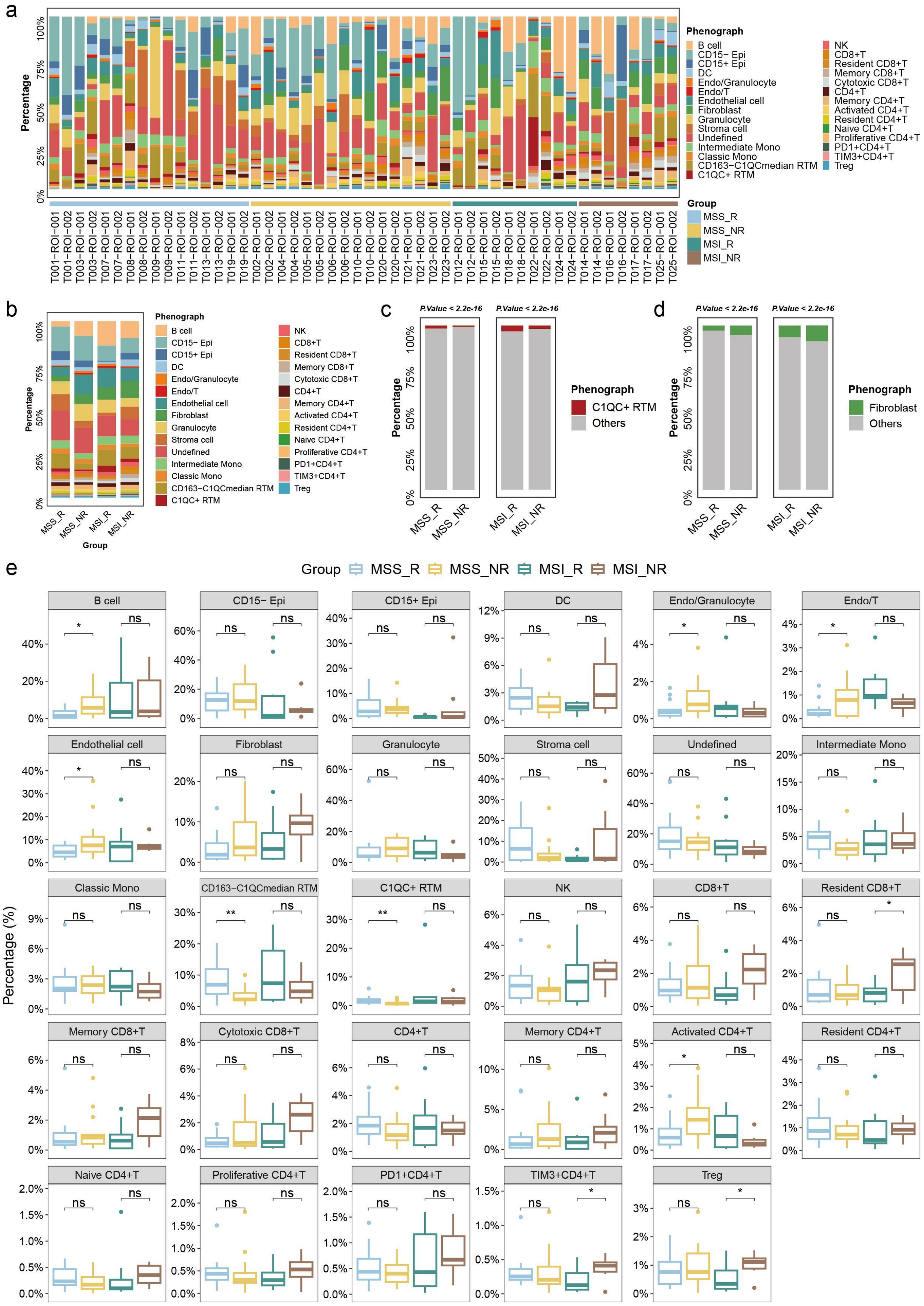
a
b
c
d
e
Percentage
Phenograph
B cell
CD15− Epi
CD15+ Epi
DC
Endo/Granulocyte
Endo/T
Endothelial cell
Fibroblast
Granulocyte
Stroma cell
Undefined
Intermediate Mono
Classic Mono
CD163−C1QCmedian RTM
C1QC+ RTM
NK
CD8+T
Resident CD8+T
Memory CD8+T
Cytotoxic CD8+T
CD4+T
Memory CD4+T
Activated CD4+T
Resident CD4+T
Naive CD4+T
Proliferative CD4+T
PD1+CD4+T
TIM3+CD4+T
Treg
Group
MSS_R
MSS_NR
MSI_R
MSI_NR
P.Value < 2.2e-16
Others
Fibroblast
Percentage (%)

Supplementary Fig. S11. Cell population composition across different groups based on IMC data. (a) Stacked bar plots displaying the percentages of various cell phenotypes in each sample. (b) The frequency of each cell phenotype across the four groups: MSS_R, MSS_NR, MSI_R, and MSI_NR. (c) Chi-square test assessing the difference in the proportion of C1QC^{+} RTMs between MSS_R and MSS_NR (left), as well as between MSI_R and MSI_NR (right). (d) Chi-square test assessing the difference in the proportion of fibroblasts between MSS_R and MSS_NR (left), as well as between MSI_R and MSI_NR (right). (e) Box plots displaying the proportions of each cell phenotype relative to the total cell populations across the four groups. Significance was evaluated by the t-tests.

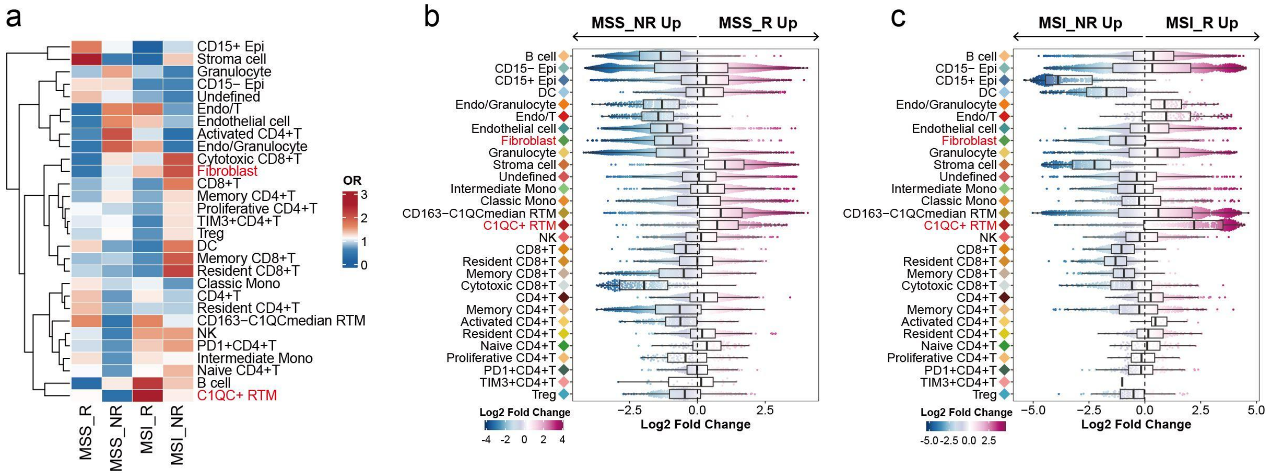

Supplementary Fig. S12. Cell population composition across different groups based on IMC data. (a) Prevalence of immunotherapy outcomes for each cell cluster, estimated by the OR analysis. (b) Beeswarm and box plots showing log2-fold differences in neighborhoods across cell type clusters using Milo differential abundance testing. Red indicates increased neighborhoods in MSS_R, and blue indicates decreased neighborhoods in MSS_NR. (c) Beeswarm and box plots showing neighborhood differences in different cell type clusters between the MSI_R and MSI_NR groups, estimated by Milo differential abundance testing.

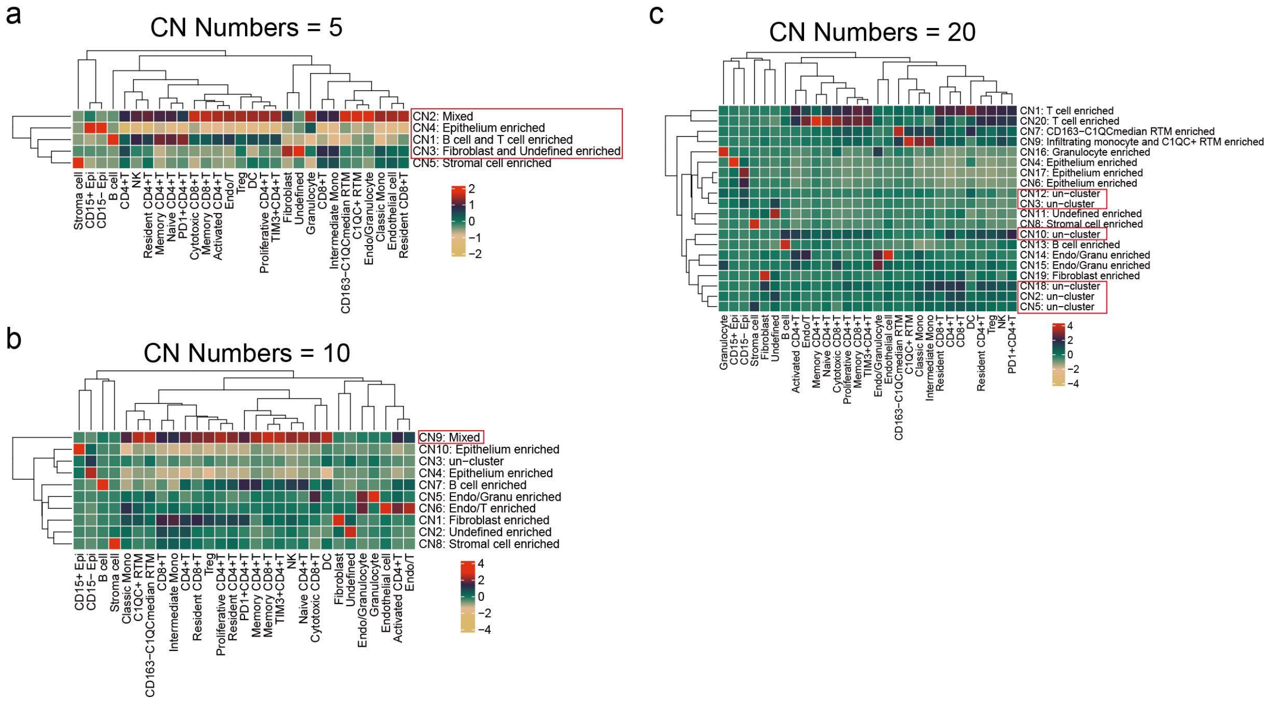

Supplementary Fig. S13. CN composition and abundance. Heatmaps depicting the composition and abundance of cellular neighborhoods (CNs) across varying numbers of distinct CNs: (a) 5 CNs, (b) 10 CNs and (c) 20 CNs. Each CN is defined based on the 29 original cell clusters, with the heatmap illustrating the relative abundance of each cell cluster within the respective CNs.

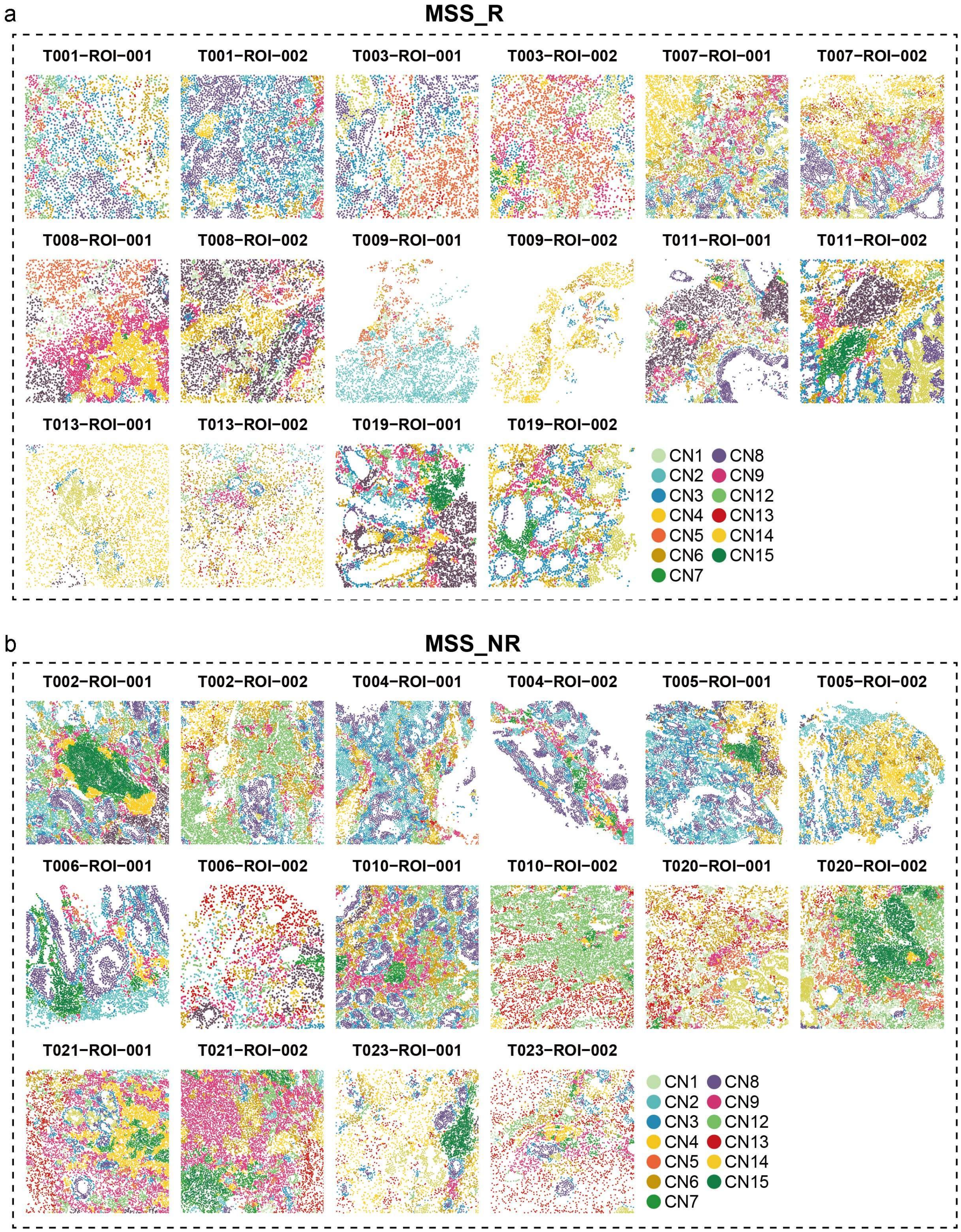

Supplementary Fig. S14. Spatial of CNs in MSS samples. (a-b) Spatial distribution of CNs in the tumor TME of MSS-R (a) and MSS-NR (b) CRC samples.

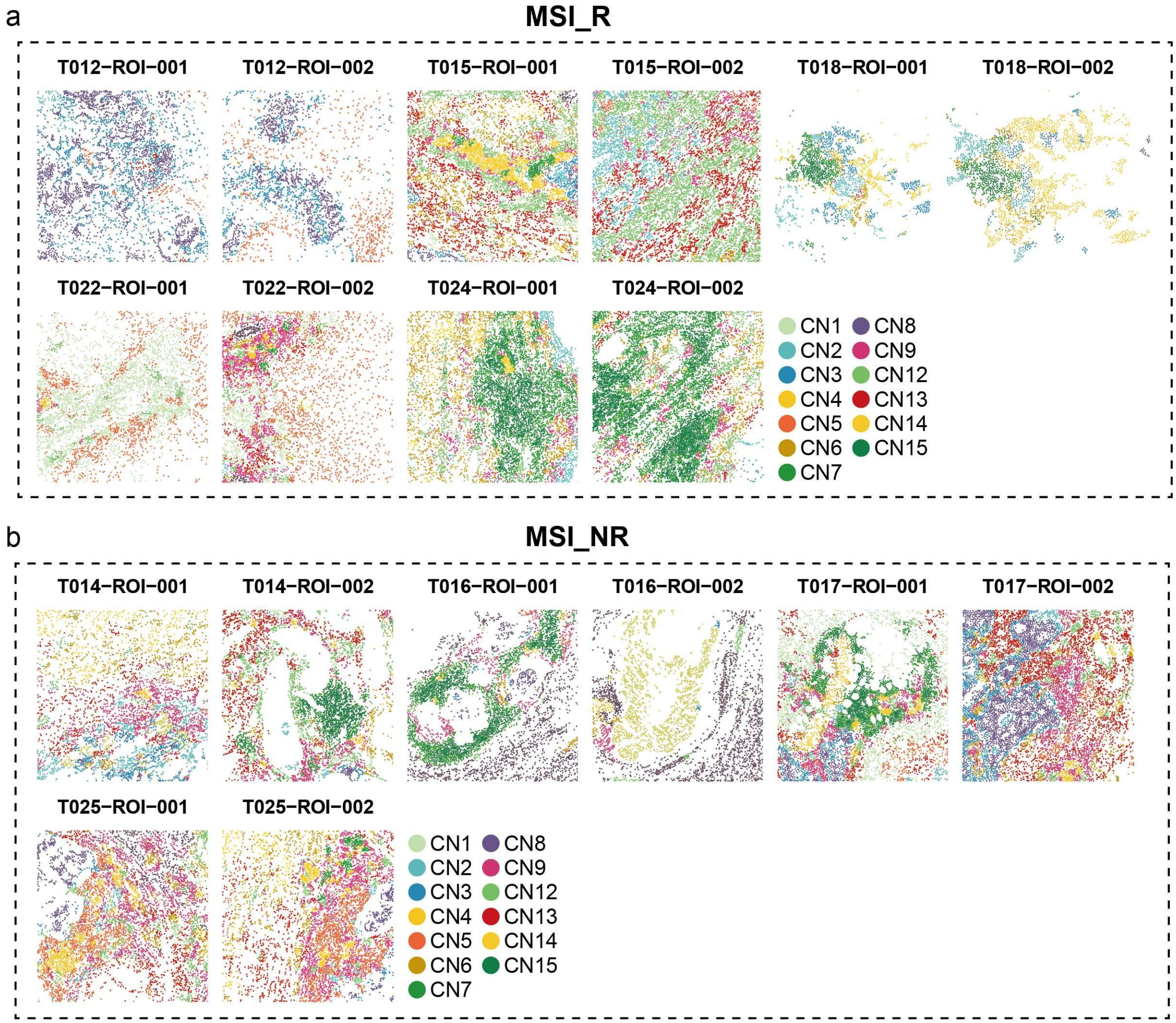

Supplementary Fig. S15. Spatial of CNs in MSI samples. (a-b) Spatial distribution of CNs in the tumor TME of MSI-R (a) and MSI-NR (b) CRC samples.

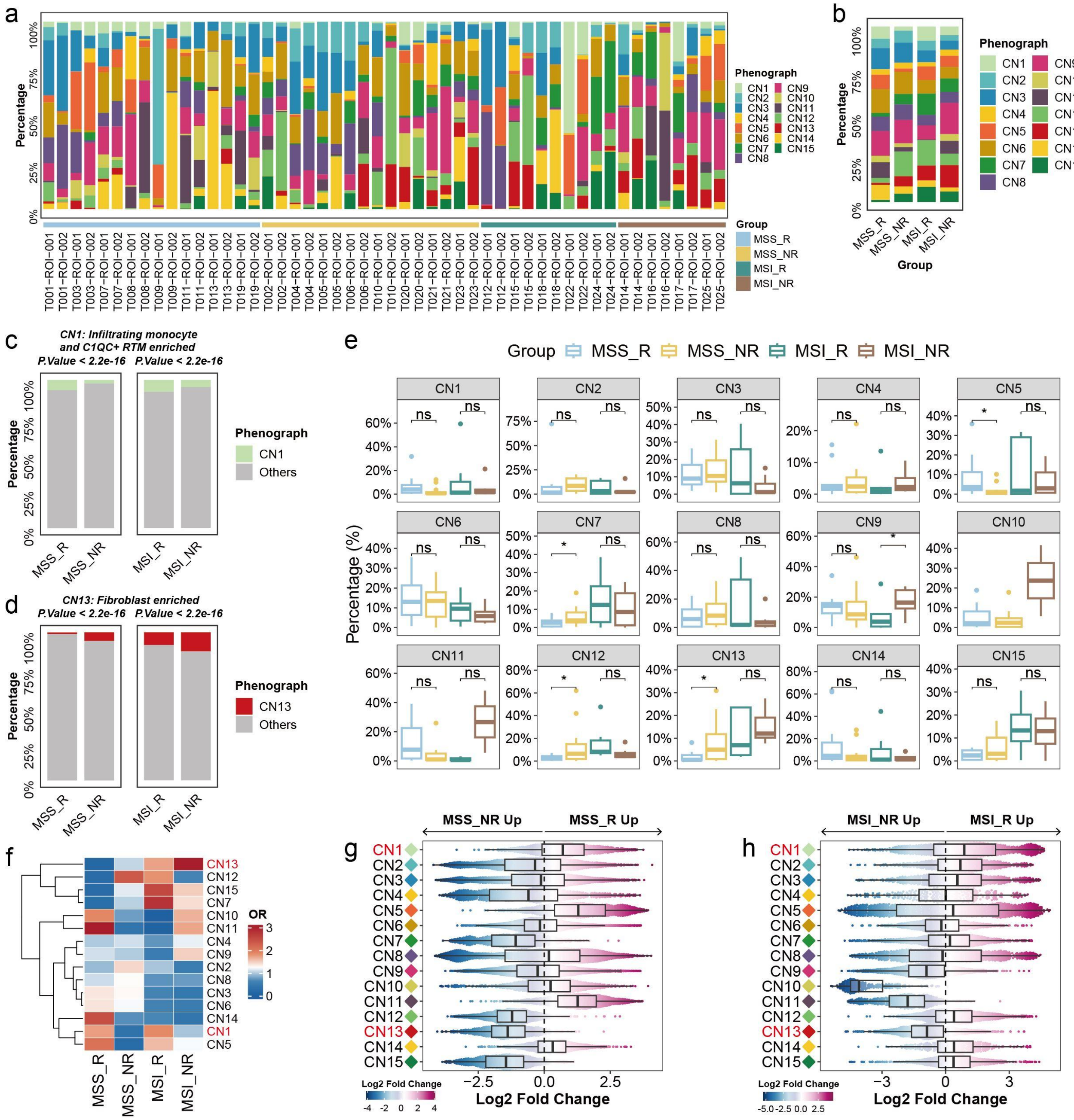

Supplementary Fig. S16. CN composition across different groups based on IMC data. (a) Stacked bar plots displaying the percentages of various CNs in each sample. (b) The frequency of each CN across the four groups. (c) Chi-square test assessing the difference in the proportion of CN1 between MSS_R and MSS_NR (left), as well as between MSI_R and MSI_NR (right). (d) Chi-square test assessing the difference in the proportion of CN13 between MSS_R and MSS_NR (left), as well as between MSI_R and MSI_NR (right). (e) Box plots displaying the proportions of each CN relative to the total CNs across the four groups. Significance was evaluated by the t-tests. (f) Prevalence of immunotherapy outcomes for each CN, estimated by the OR analysis. (g-h) Beeswarm and box plots showing neighborhood differences in different CNs between the MSS_R

and MSS_NR groups (g), as well as between MSI_R and MSI_NR (h), estimated by Milo differential abundance testing.

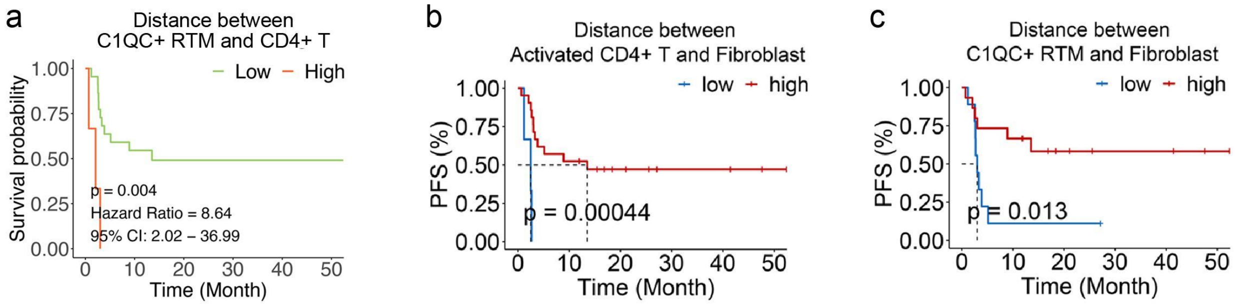

Supplementary Fig. S17. Spatial proximity of immune cells and its impact on prognosis. (a) Kaplan-Meier plot showing the association between the distance of $C1QC^+$ RTM from $CD4^+$ T and patient prognosis (b) Kaplan-Meier plot showing the association between the distance of activated $CD4^+$ T cells from fibroblasts and patient prognosis. (c) Kaplan-Meier plot showing the association between the distance of $C1QC^+$ RTM cells from fibroblasts and patient prognosis. The Univariate Cox Test was adopted to evaluate the statistical significance. The group with high and low distances was stratified using the best cut-off method from “survminer” package.

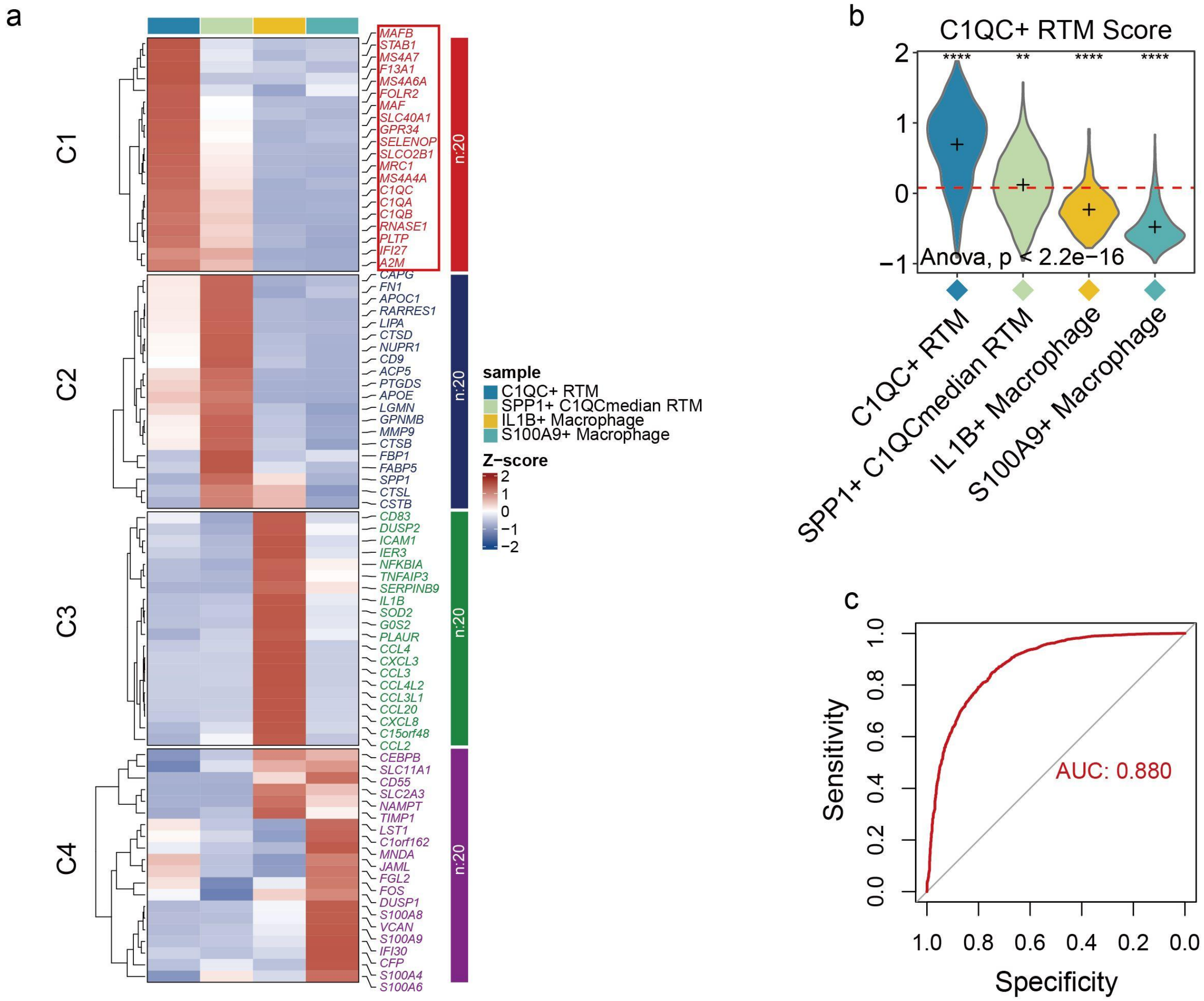

Supplementary Fig. S18. Specificity of the C1QC$^+$ RTM signature genes. (a) Heatmap displaying the expression of top 20 marker genes specifically associated with each macrophage subpopulation. (b) C1QC$^+$ RTM scores across distinct monocyte and macrophage subpopulations, calculated using the AddModuleScore method based on the C1QC$^+$ RTM gene signature. (c) Receiver operating characteristic (ROC) curve evaluating the ability of the C1QC$^+$ RTM score to distinguish C1QC$^+$ RTM from other macrophage subpopulations.

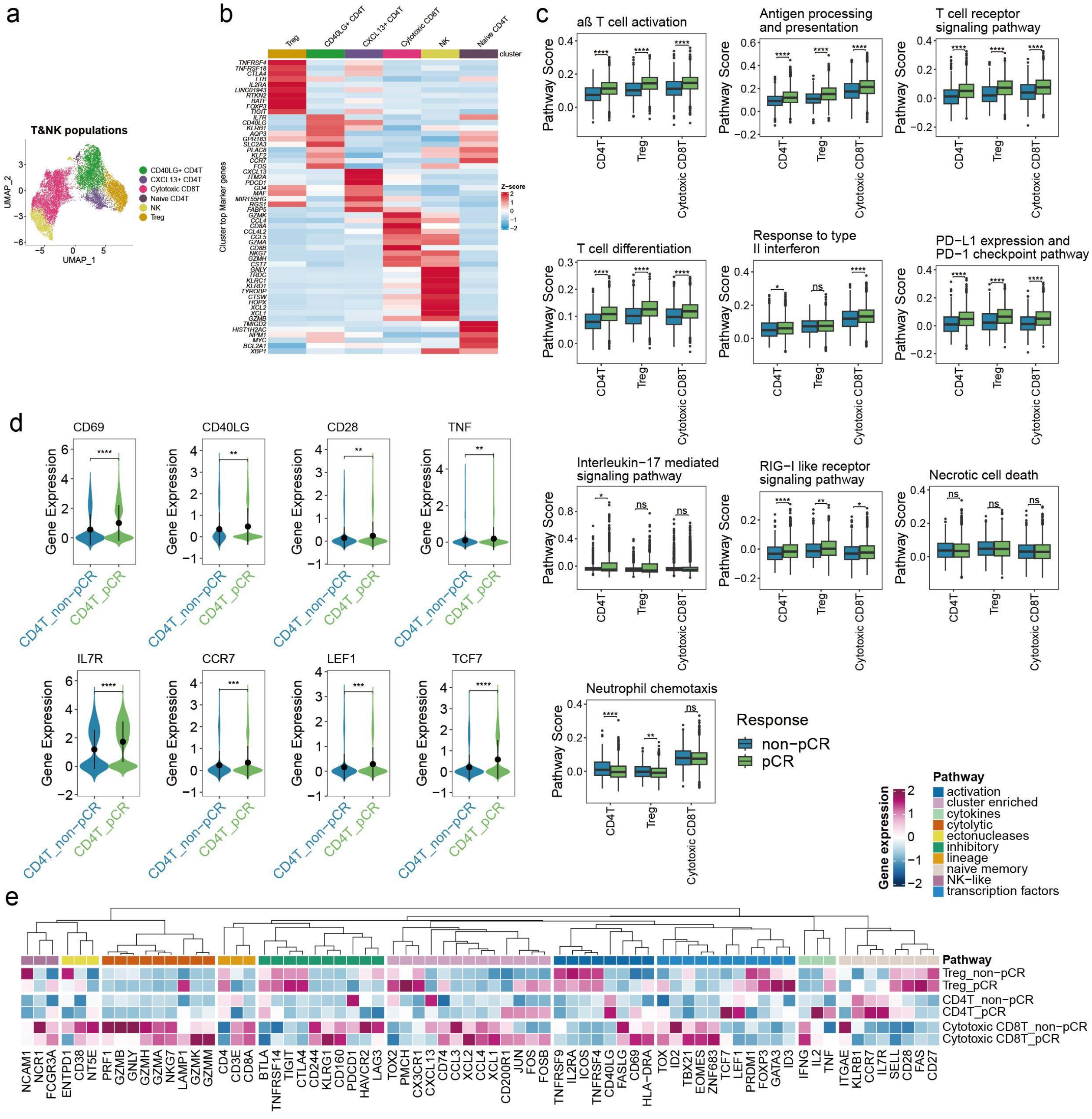

Supplementary Fig. S19. Molecular and pathway characteristics of CD4^{+} T cell activation in pCR. (a) UMAP plot of CD40LG^{+} CD4^{+} T cell, CXCL13^{+} CD4^{+} T cell, cytotoxic CD8^{+} T cell, naïve CD4^{+} T cell, NK cell and Treg. (b) Top marker genes of T/NK cells for further cell-cell interaction analysis. (c) Box plot comparing the pathway activities of T cell-related pathways in pCR and non-pCR tissues. (d) Violin plot showing the expression levels of T cell-related genes in pCR and non-pCR tissues. (e) Heatmap illustrating the expression levels of T cell-related genes in CD4^{+} T cells, CD8^{+} T cells, and Tregs in pCR and non-pCR tissues, colored by z-score normalized expression levels.

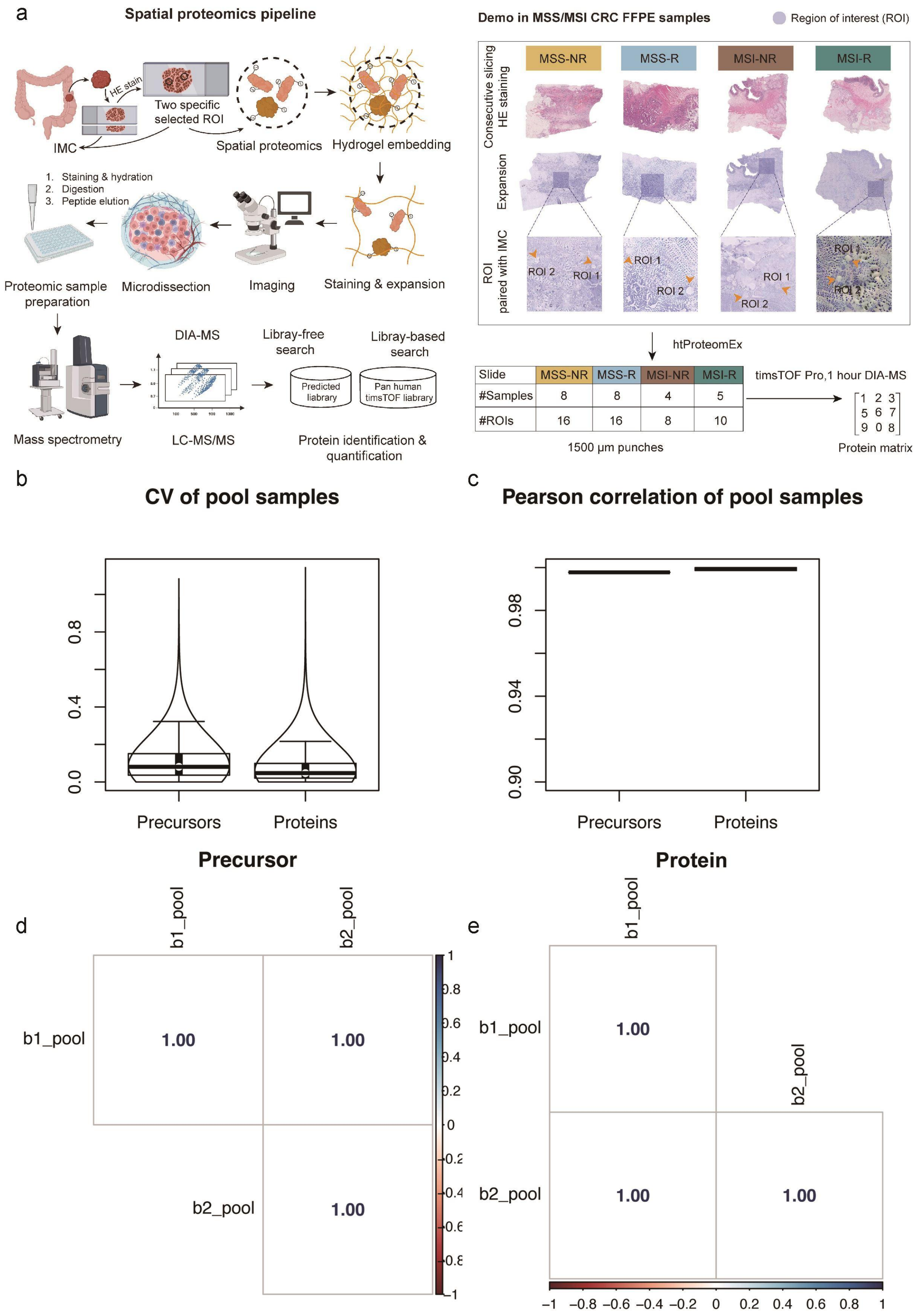
a
Spatial proteomics pipeline
HE stain
Two specific selected ROI
IMC
Spatial proteomics
Hydrogel embedding
1. Staining & hydration
2. Digestion
3. Peptide elution
Proteomic sample preparation
Microdissection
Imaging
Staining & expansion
DIA-MS
Libray-free search
Libray-based search
Predicted liabrary
Pan human timsTOF liabrary
Mass spectrometry
LC-MS/MS
Protein identification & quantification
Demo in MSS/MSI CRC FFPE samples
Region of interest (ROI)
MSS-NR
MSS-R
MSI-NR
MSI-R
Consecutive slicing HE staining
Expansion
ROI paired with IMC
ROI 1
ROI 2
htProteomEx
Slide
#Samples
#ROIs
8
8
4
5
16
16
8
10
1500 μm punches
timsTOF Pro,1 hour DIA-MS
Protein matrix
b
CV of pool samples
Precursors
Proteins
c
Pearson correlation of pool samples
Precursors
Proteins
Precursor
d
b1_pool
b2_pool
1.00
Protein
e
b1_pool
b2_pool
1.00

Supplementary Fig. S20. Quality control of identification results of spatial proteomics. (a) The workflow of spatial proteomics and demo in MSS/MSI samples. 25 patients with 50 regions of interest (ROIs) were analyzed. (b) Coefficient of variation of the global precursors and proteins. (c) Pearson correlation distribution of the global precursors and proteins. (d-e) Pearson correlations of proteins and peptides quantification of pool samples.

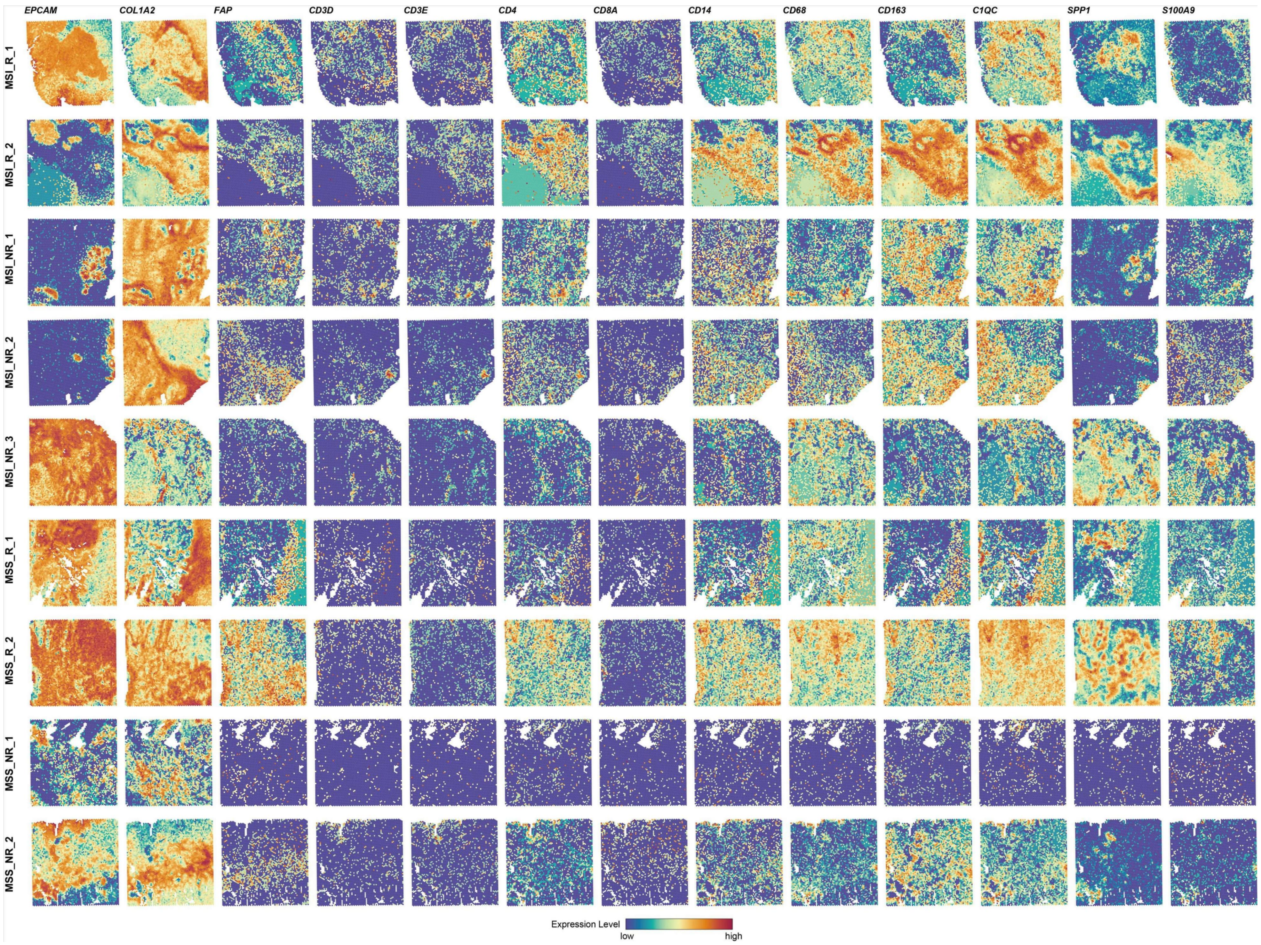

Supplementary Fig. S21. The spatial feature plot showing the expression level of lineage genes in 9 in-house CRC samples.

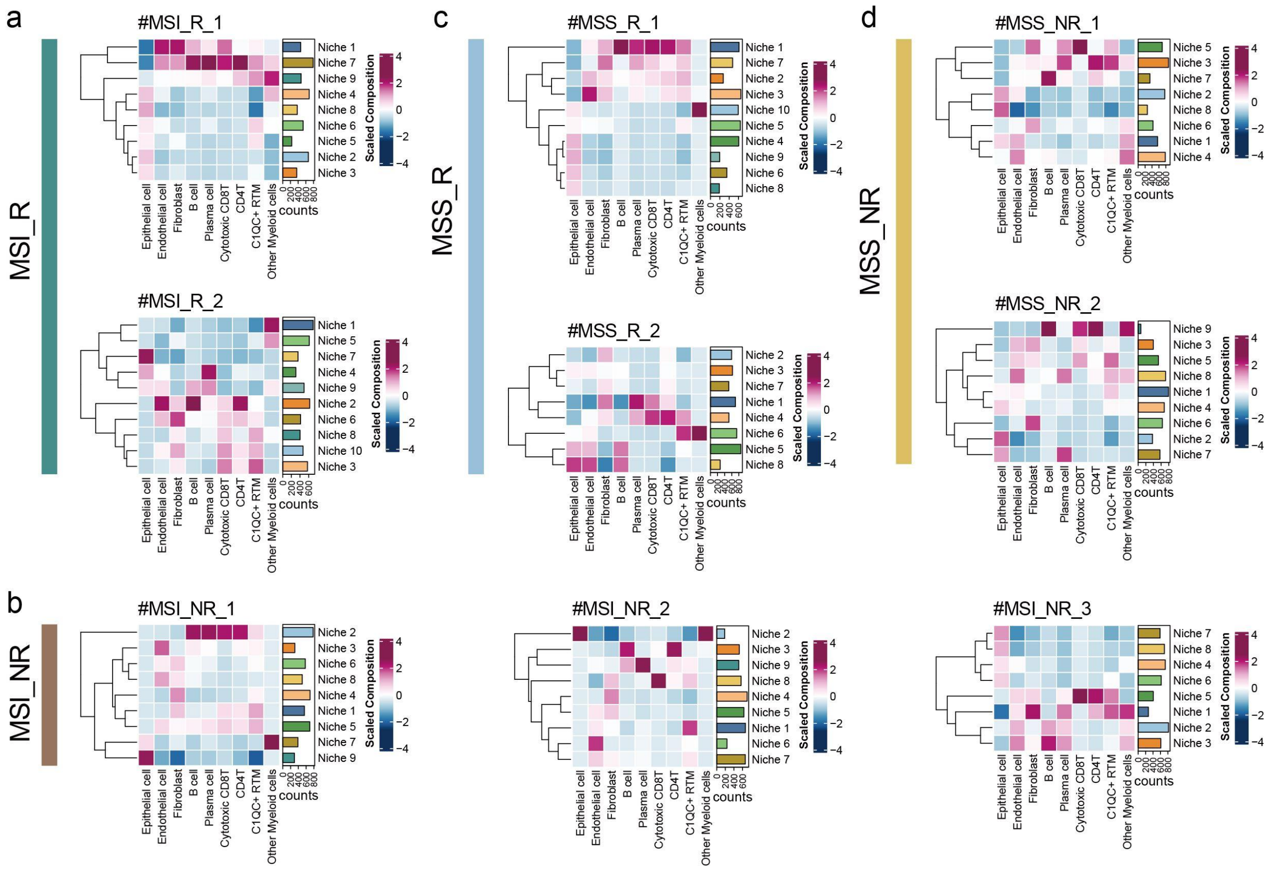

Supplementary Fig. S22. Distinct cellular niche compositions across MSI and MSS CRC. (a-d) Heatmaps displaying the predominant cell types within each niche for MSI_R (a), MSI_NR (b), MSS_R (c), and MSS_NR (d) samples.

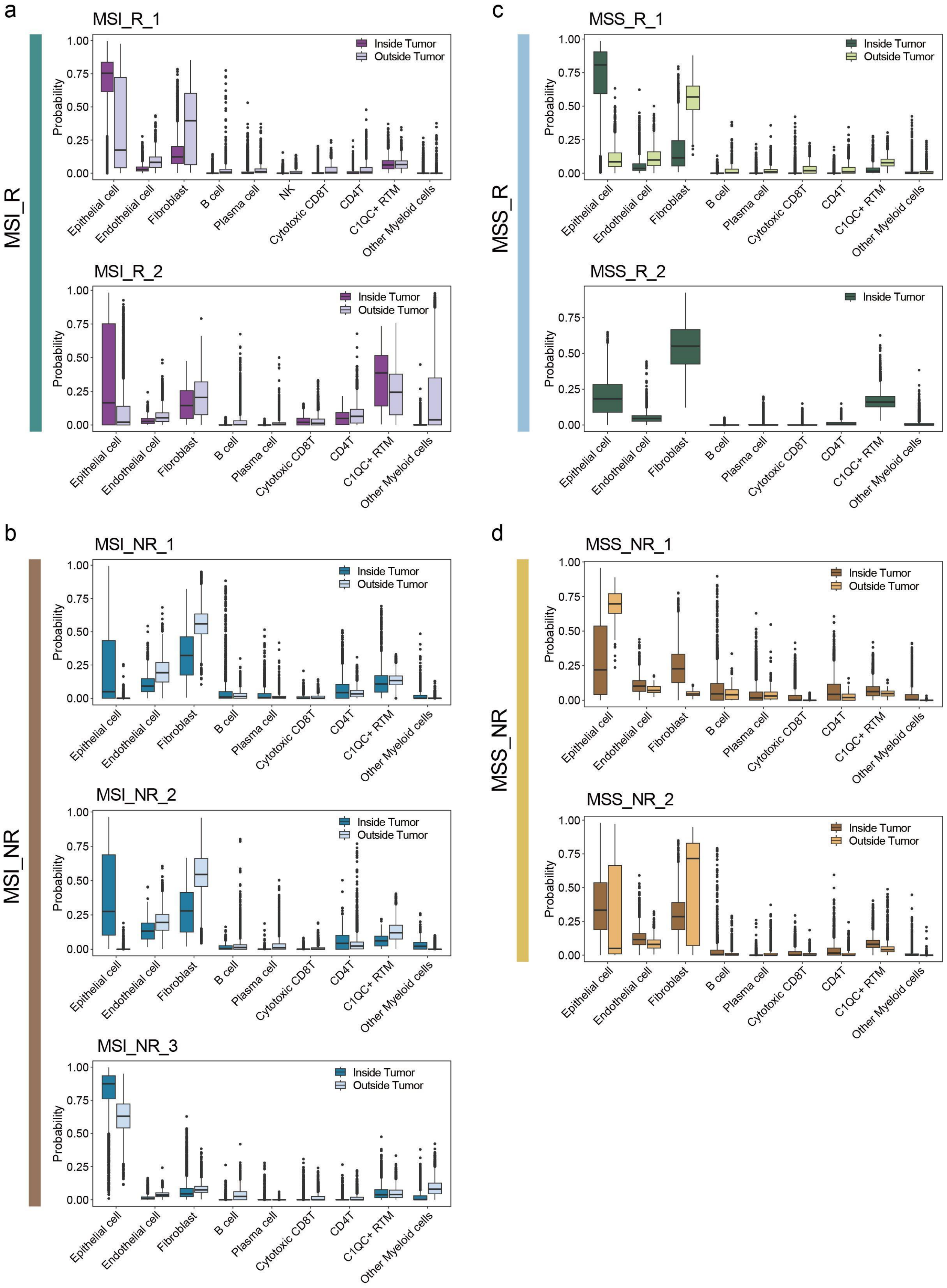
a
MSI_R
MSI_R_1
MSI_R_2
b
MSI_NR
MSI_NR_1
MSI_NR_2
MSI_NR_3
c
MSS_R
MSS_R_1
MSS_R_2
d
MSS_NR
MSS_NR_1
MSS_NR_2
Inside Tumor
Outside Tumor
Probability
1.00
0.75
0.50
0.25
0.00
Epithelial cell
Endothelial cell
Fibroblast
B cell
Plasma cell
NK
Cytotoxic CD8T
CD4T
C1QC+ RTM
Other Myeloid cells

Supplementary Fig. S23. Spatial distribution of cell clusters inside and outside the tumor. The spatial distribution and intensities of cell clusters are shown for (a) MSI_R, (b) MSI_NR, (c) MSS_R, and (d) MSS_NR. The MSS_R_2 section contains only intratumoral tissue.

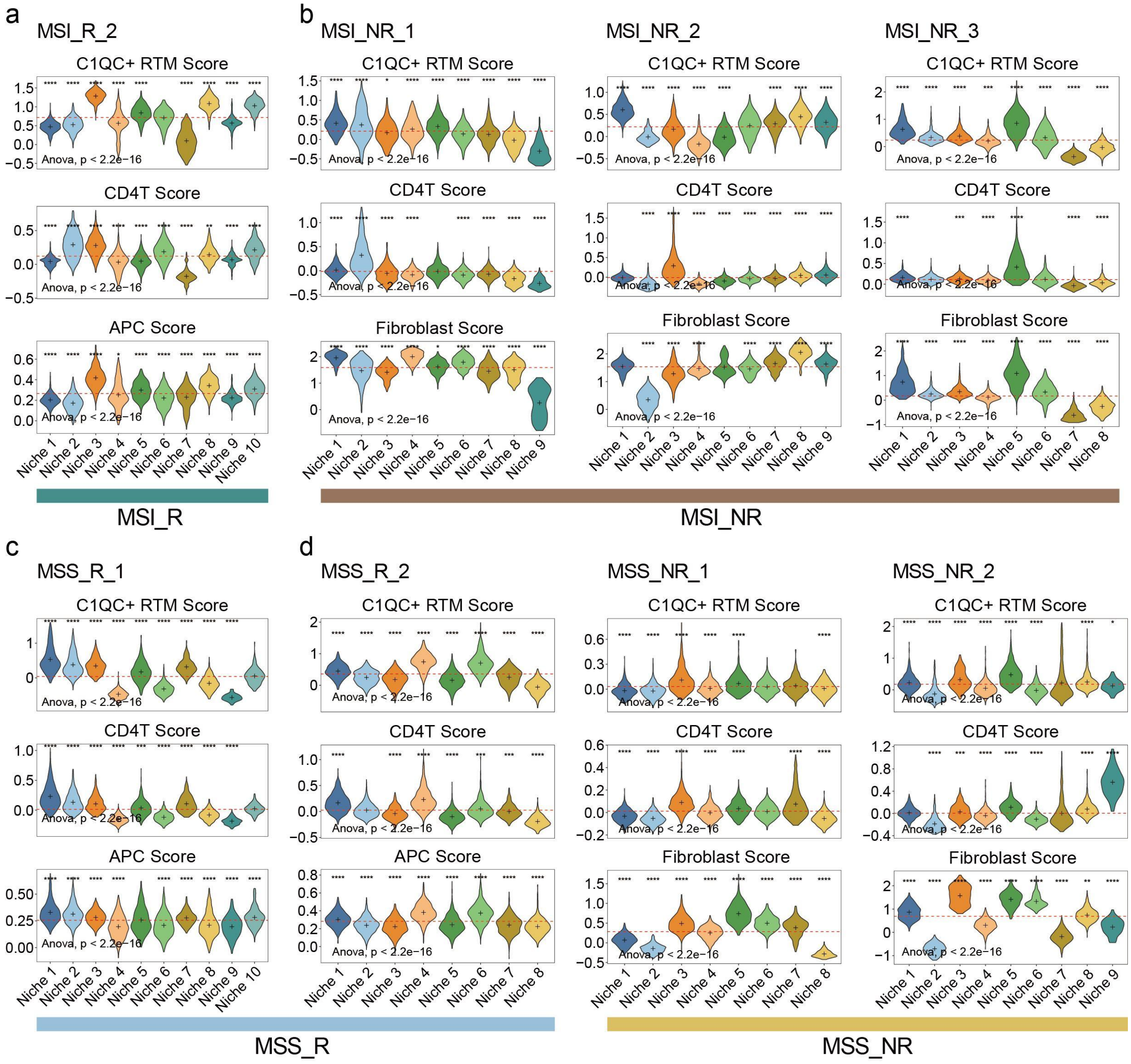

Supplementary Fig. S24. Violin plots illustrating the gene set scoring results for each niche in the MSI_R (a), MSI_NR (b), MSS_R (c), and MSS_NR (d) samples.

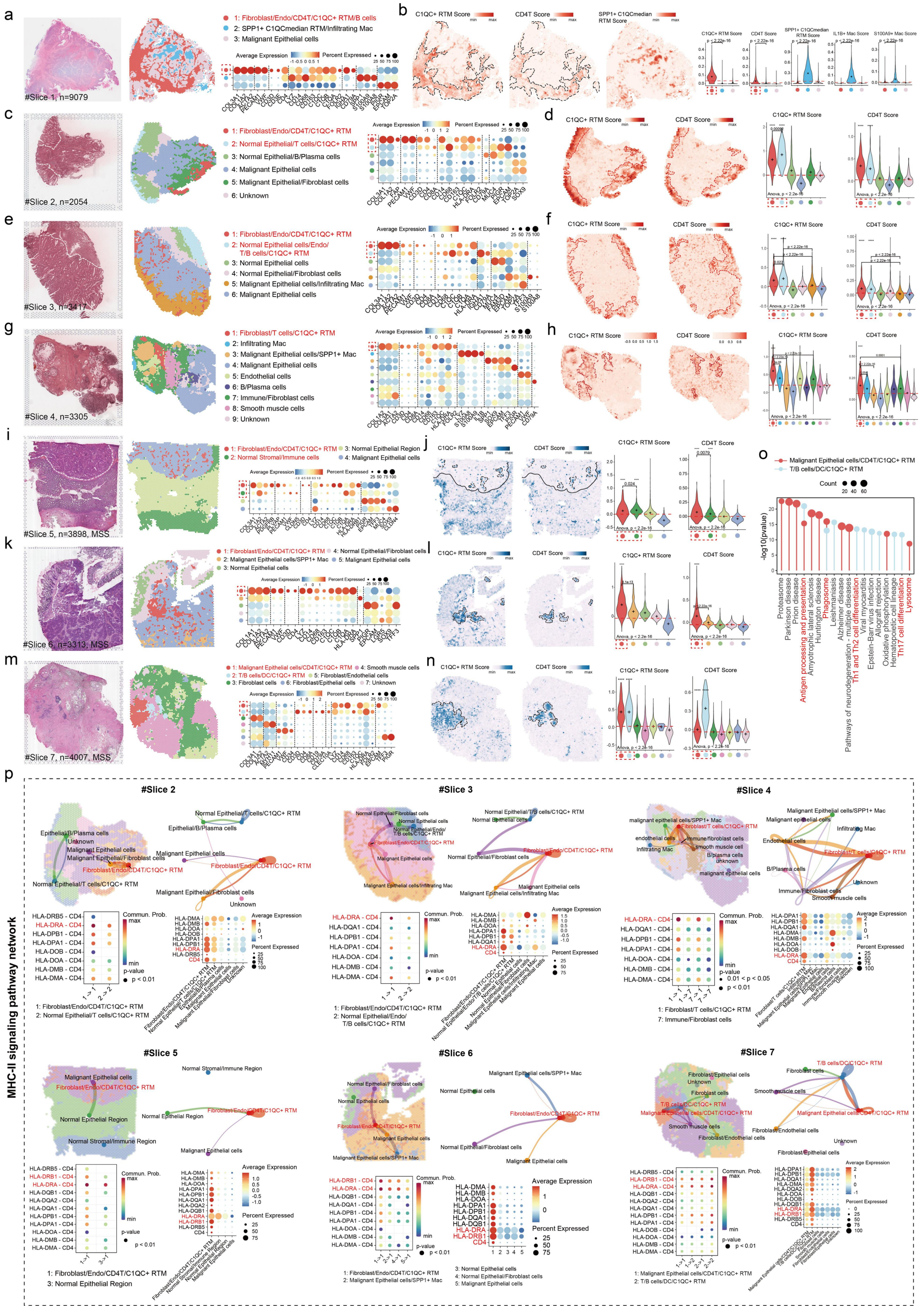

Supplementary Fig. S25. C1QC^{+} RTM and CD4^{+} T cells co-locate and interact through the MHC-II signaling pathway revealed by spatial transcriptomic data from external cohort. (a, c, e, g, i, k, m) H&E staining of tissue sections (left), unbiased clustering and annotation of the spots (middle), and dot plots showing average expression of classical markers in each cluster (right) in 7 CRC slices from the external cohort. The white dashed line in the H&E section separated the para tumor from the tumor. (b) Through AddModuleScore-based gene set scoring, spatial feature plots and violin plots showed signature scores of C1QC^{+} RTM, CD4^{+} T cells, and other macrophage subsets in #slice 1. The dashed line in violin plots indicated the mean signature score of all clusters. (d, f, g, h, j, l, n) Through AddModuleScore-based gene set scoring, spatial feature plots and violin plots showed signature scores of C1QC^{+} RTM and CD4^{+} T cells in 6 CRC slices. The dashed line in violin plots indicated the mean signature score of all clusters. The One-way Anova Test were adopted to evaluate the statistical significance. (o) KEGG enrichment of clusters containing C1QC^{+} RTM in #slice 7. (p) Spatial interaction plots and circle plots showed differences in the MHC-II pathway interaction of various clusters. The thicker the line, the stronger the connection. The dot plot showed the contribution degree of the ligand-receptor pairs and the expression level of ligand-receptor genes in each cluster.

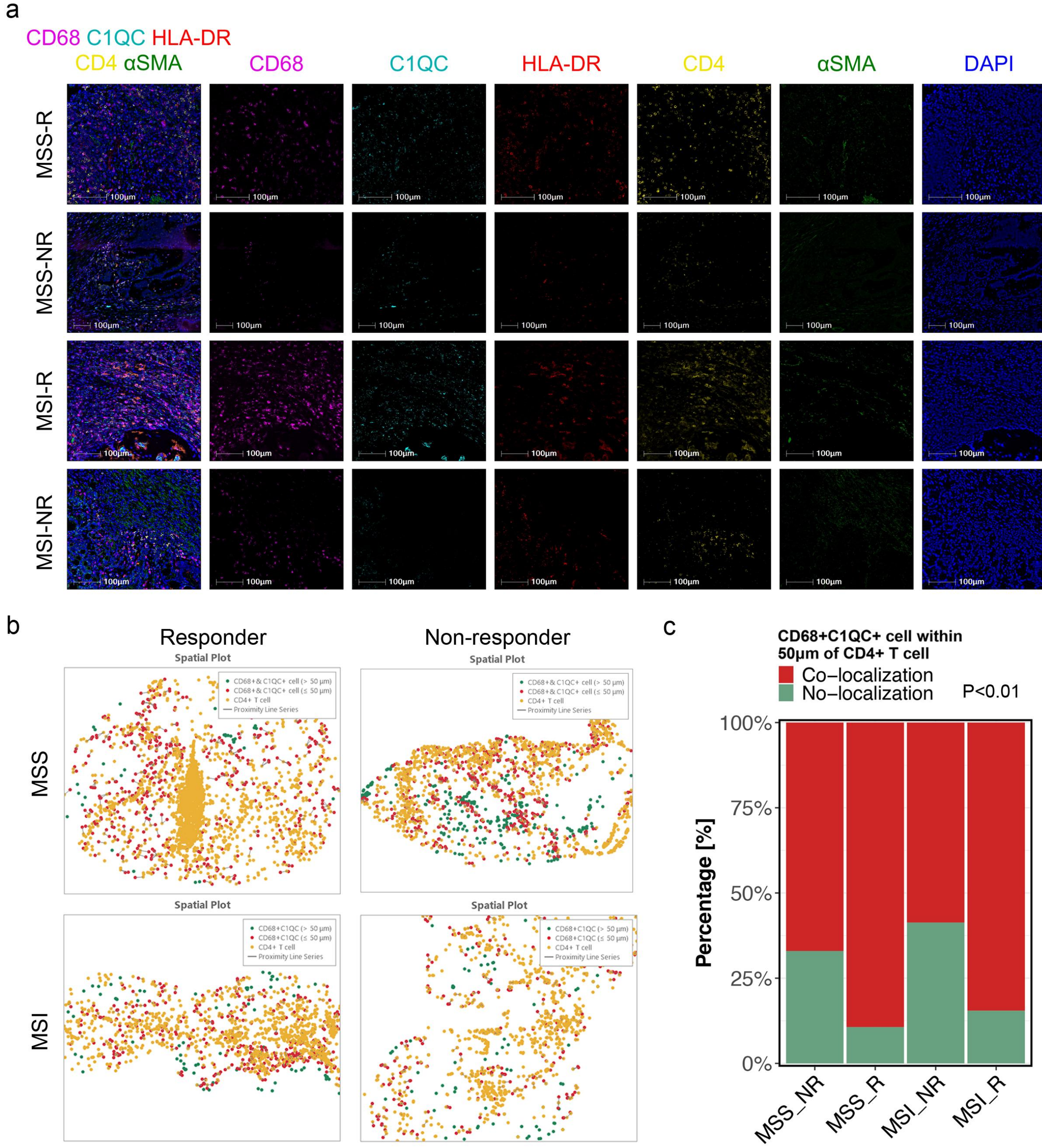

Supplementary Fig. S26. The mIF staining to detect the co-localization of CD4^{+} T cells and C1QC^{+} CD68^{+} cells. (a) The staining of CD4 (yellow), CD68 (magenta), C1QC (cyan), HLA-DR (red), αSMA (green) and DAPI (blue) in per group. (b) The spatial plot for each group showing the co-localization of C1QC^{+} CD68^{+} cells and CD4^{+} T cells (defined as a distance of less than 50μm between C1QC^{+} CD68^{+} cells and CD4^{+} T cells). (c) The proportion of co-localization for each group. *n* = 4 for each group. The Chi-square test was applied to detect the significance.

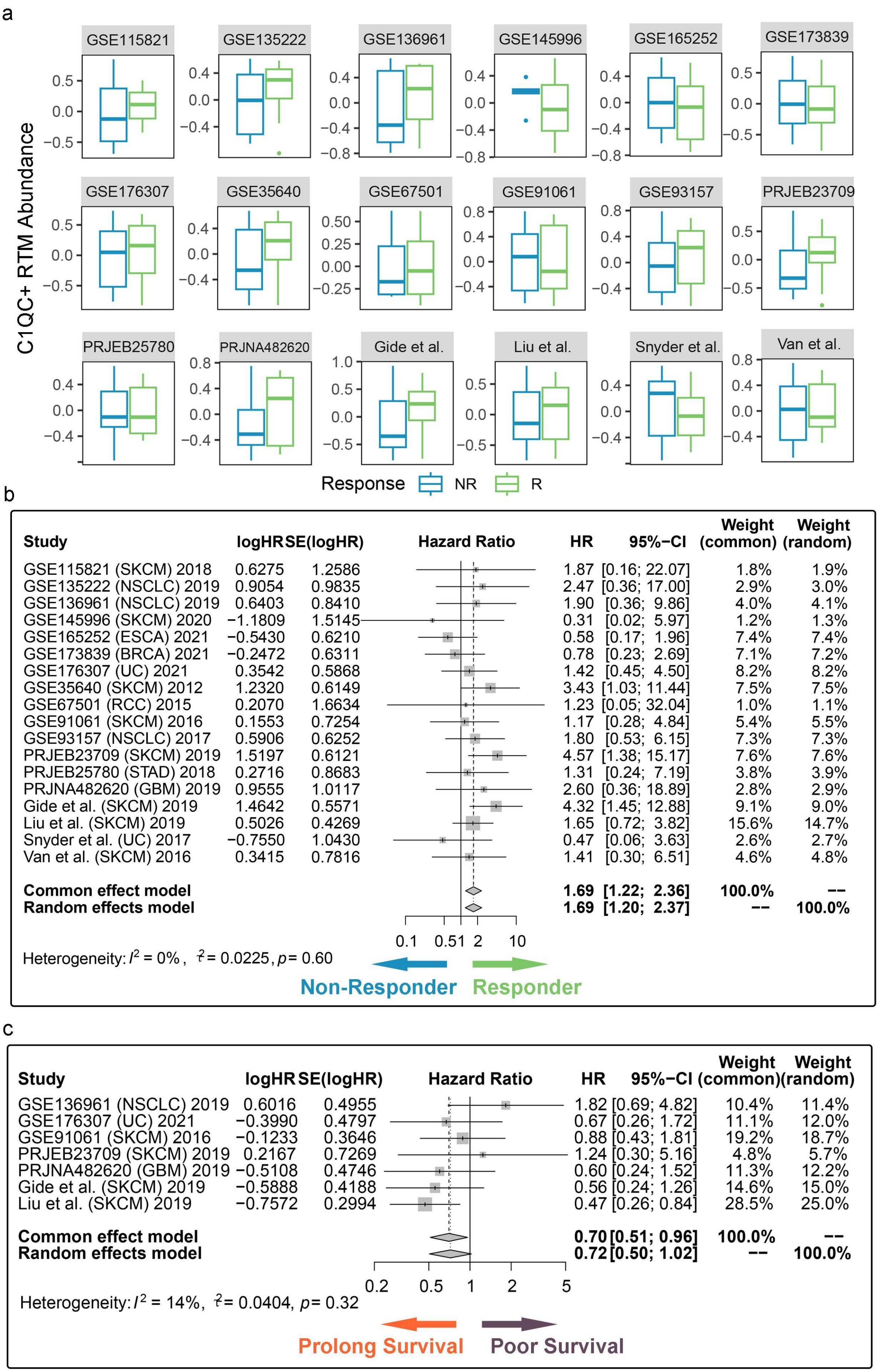
a
C1QC+ RTM Abundance
GSE115821
GSE135222
GSE136961
GSE145996
GSE165252
GSE173839
GSE176307
GSE35640
GSE67501
GSE91061
GSE93157
PRJEB23709
PRJEB25780
PRJNA482620
Gide et al.
Liu et al.
Snyder et al.
Van et al.
Response NR R
b
Study logHR SE(logHR) Hazard Ratio HR 95%−CI Weight (common) Weight (random)
GSE115821 (SKCM) 2018 0.6275 1.2586 1.87 [0.16; 22.07] 1.8% 1.9%
GSE135222 (NSCLC) 2019 0.9054 0.9835 2.47 [0.36; 17.00] 2.9% 3.0%
GSE136961 (NSCLC) 2019 0.6403 0.8410 1.90 [0.36; 9.86] 4.0% 4.1%
GSE145996 (SKCM) 2020 −1.1809 1.5145 0.31 [0.02; 5.97] 1.2% 1.3%
GSE165252 (ESCA) 2021 −0.5430 0.6210 0.58 [0.17; 1.96] 7.4% 7.4%
GSE173839 (BRCA) 2021 −0.2472 0.6311 0.78 [0.23; 2.69] 7.1% 7.2%
GSE176307 (UC) 2021 0.3542 0.5868 1.42 [0.45; 4.50] 8.2% 8.2%
GSE35640 (SKCM) 2012 1.2320 0.6149 3.43 [1.03; 11.44] 7.5% 7.5%
GSE67501 (RCC) 2015 0.2070 1.6634 1.23 [0.05; 32.04] 1.0% 1.1%
GSE91061 (SKCM) 2016 0.1553 0.7254 1.17 [0.28; 4.84] 5.4% 5.5%
GSE93157 (NSCLC) 2017 0.5906 0.6252 1.80 [0.53; 6.15] 7.3% 7.3%
PRJEB23709 (SKCM) 2019 1.5197 0.6121 4.57 [1.38; 15.17] 7.6% 7.6%
PRJEB25780 (STAD) 2018 0.2716 0.8683 1.31 [0.24; 7.19] 3.8% 3.9%
PRJNA482620 (GBM) 2019 0.9555 1.0117 2.60 [0.36; 18.89] 2.8% 2.9%
Gide et al. (SKCM) 2019 1.4642 0.5571 4.32 [1.45; 12.88] 9.1% 9.0%
Liu et al. (SKCM) 2019 0.5026 0.4269 1.65 [0.72; 3.82] 15.6% 14.7%
Snyder et al. (UC) 2017 −0.7550 1.0430 0.47 [0.06; 3.63] 2.6% 2.7%
Van et al. (SKCM) 2016 0.3415 0.7816 1.41 [0.30; 6.51] 4.6% 4.8%
Common effect model 1.69 [1.22; 2.36] 100.0% --
Random effects model 1.69 [1.20; 2.37] -- 100.0%
Heterogeneity: I² = 0%, τ² = 0.0225, p = 0.60
Non-Responder
Responder
c
Study logHR SE(logHR) Hazard Ratio HR 95%−CI Weight (common) Weight (random)
GSE136961 (NSCLC) 2019 0.6016 0.4955 1.82 [0.69; 4.82] 10.4% 11.4%
GSE176307 (UC) 2021 −0.3990 0.4797 0.67 [0.26; 1.72] 11.1% 12.0%
GSE91061 (SKCM) 2016 −0.1233 0.3646 0.88 [0.43; 1.81] 19.2% 18.7%
PRJEB23709 (SKCM) 2019 0.2167 0.7269 1.24 [0.30; 5.16] 4.8% 5.7%
PRJNA482620 (GBM) 2019 −0.5108 0.4746 0.60 [0.24; 1.52] 11.3% 12.2%
Gide et al. (SKCM) 2019 −0.5888 0.4188 0.56 [0.24; 1.26] 14.6% 15.0%
Liu et al. (SKCM) 2019 −0.7572 0.2994 0.47 [0.26; 0.84] 28.5% 25.0%
Common effect model 0.70 [0.51; 0.96] 100.0% --
Random effects model 0.72 [0.50; 1.02] -- 100.0%
Heterogeneity: I² = 14%, τ² = 0.0404, p = 0.32
Prolong Survival
Poor Survival

Supplementary Fig. S27. The C1QC$^+$ RTM abundance is an effective biomarker of survival and immunotherapy efficacy in pan-cancer patients. (a) Boxplot showing the median abundance of C1QC$^+$ RTM stratified from different Gene Expression Omnibus (GEO) cohorts, across treatment response groups. (b) Hazard ratio of C1QC$^+$ RTM abundance in responder and non-responder across each GEO cohort. (c) Hazard ratio of C1QC$^+$ RTM abundance in survival.

Supplementary Table S1. Clinical metadata for enrolled 25 patients

Pts	Age	Gender	Tumor Location	TNM	Tumor stage	dMMR/ pMMR	MSI/ MSS	TMB (Muts/Mb)	ORR	Treatment Regimen
P01	68	Male	Sigmoid colon	T4N1M1	IV	pMMR	MSS	NA	CR	Sintilimab +Regrofenib
P02	43	Male	Sigmoid colon	T3N1M1	IV	pMMR	MSS	NA	SD	Toripalimab +Regrofenib
P03	61	Male	Ascending colon	T3N1M1	IV	pMMR	MSS	NA	CR	Sintilimab +Regrofenib
P04	60	Female	Sigmoid colon	T4N2M1	IV	pMMR	MSS	NA	PR	Sintilimab +Regrofenib
P05	49	Male	Low rectum	T3N1M1	IV	pMMR	MSS	NA	PR	Tislelizumab +Regrofenib
P06	57	Male	Low rectum	T4N1M1	IV	pMMR	MSS	NA	SD	Sintilimab +Regrofenib
P07	66	Male	Low rectum	T3N2M1	IV	pMMR	MSS	NA	SD	Sintilimab +Regrofenib
P08	60	Male	Descending colon	T3N2M1	IV	pMMR	MSS	NA	PR	Sintilimab +Regrofenib
P09	60	Male	Ascending colon	T4N1M1	IV	pMMR	MSS	4.23	PD	Sintilimab +Regrofenib
P10	64	Female	Low rectum	T3N1M1	IV	pMMR	MSS	5.29	PD	Sintilimab +Regrofenib
P11	46	Female	Sigmoid colon	T3N1M1	IV	pMMR	MSS	0	PD	Sintilimab +Regrofenib
P12	71	Male	Descending colon	T4N1M1	IV	pMMR	MSS	6.34	PD	Sintilimab +Regrofenib
P13	44	Male	Low rectum	T3N2M1	IV	pMMR	MSS	4.23	PD	Sintilimab +Regrofenib
P14	60	Male	Sigmoid colon	T4N1M1	IV	pMMR	MSS	9.2	PD	Sintilimab +Regrofenib
P15	51	Male	Low rectum	T3N2M1	IV	pMMR	MSS	NA	PD	Sintilimab +Regrofenib
P16	66	Male	Sigmoid colon	T3N1M1	IV	pMMR	MSS	NA	PD	Sintilimab +Regrofenib
P17	59	Male	Ascending colon	T4N1M1	IV	dMMR	MSI	112.95	SD	QL1706 (PD-1 antibody)
P18	73	Male	Ascending colon	T3N2M1	IV	dMMR	MSI	77.7	PR	Pembrolizumab
P19	33	Female	Descending colon	T4N2M1	IV	dMMR	MSI	50.85	PR	Pembrolizumab
P20	60	Female	Transverse colon	T3N1M1	IV	dMMR	MSI	NA	PR	Pembrolizumab
P21	34	Male	Descending colon	T4N1M1	IV	dMMR	MSI	NA	SD	Pembrolizumab
P22	28	Female	Sigmoid colon	T4N2M1	IV	dMMR	MSI	NA	PD	Pembrolizumab
P23	74	Female	Descending colon	T4N2M1	IV	dMMR	MSI	49.27	PD	Toripalimab

P24	47	Female	Ascending colon	T3N1M1	IV	dMMR	MSI	NA	PD	Sintilimab
P25	43	Male	Ascending colon	T3N1M1	IV	dMMR	MSI	35.89	PD	Sintilimab

Supplementary Table S2. Clinical characteristics of enrolled patients

	MSS NR	MSS R	*P* value	MSI NR	MSI R
**Age (years)**			1.0		
Range	41-70	48-75		28-77	35-73
> 50	6	6		1	3
≤50	2	2		3	2
**Sex**			0.52		
Male	7	6		1	3
Female	1	2		3	2
**Anatomic**			1.0		
Left-sided	7	7		2	2
Right- sided	1	1		2	3
**Gene mutation**			0.57	NA	NA
Wild type	5	4			
RAS mutation	3	3			
BRAF mutation	0	1			
**Metastasis organ**					
Liver	6	4	0.30	0	1
Lung	3	5	0.32	0	1
Peritoneal	2	0	0.13	4	1
Other	4	2	0.30	2	4
**Treatment lines**			1.0		
First line	0	0		4	5
Third line	8	8		0	0

Supplementary Table S3. IMC antibody list

Channel	Marker	Source	Identifier	Clone
115In	S100A9	abcam	ab271864	EPR3555
141Pr	CD14	abcam	ab226121	EPR3653
142Nd	FOXP3	CST	74816SF	D2W8E
143Nd	CD16	abcam	ab256582	EPR16784
144Nd	HLA-1	abcam	ab239788	EPR22172
145Nd	CD4	abcam	ab181724	EPR6855
146Nd	CD8	biolegend	372902	C8/144B
147Sm	Collagen I	abcam	ab215969	EPR7785
148Nd	CXCL13	abcam	ab270408	EPR23400-92
149Sm	CD31	CST	85873SF	89C2
150Nd	TIM3	abcam	ab242080	EPR22241
151Eu	HIF1a	abcam	ab210073	EP1215Y
152Sm	CD45	CST	47937SF	D9M8I
153Eu	CD38	abcam	ab226034	EPR4106
154Sm	CD163	abcam	ab215976	EPR14643-36
155Gd	CD103	abcam	ab254201	EPR22590-27

156Gd	PD-L1	abcam	ab226766	73-10
158Gd	CCR7	abcam	ab272938	EPR23192-57
159Tb	CD68	biolegend	916104	KP1
160Gd	CD11b	abcam	ab209970	EPR1344
161Dy	CD20	abcam	ab213033	IGEL/773
162Dy	CD11c	abcam	ab216655	EP1347Y
163Dy	CD15	biolegend	323035	W6D3
164Dy	Granzyme B	abcam	ab219803	EPR20129-217
165Ho	PD-1	CST	63815SF	D4W2J
166Er	Ki-67	BD	550609	B56
168Er	HLA-DR	NOVUS	NB600-989	TAL 1B5
169Tm	eIF1A	abcam	ab243919	EPR12466（B）
170Er	CD3	CST	24581SF	D7A6E
171Yb	TNFα	proteintech	60291-1-Ig	7B8A11
172Yb	CD208	abcam	ab281573	EPR24265-8
173Yb	CD45RO	biolegend	304239	UCHL1
174Yb	CD57	BD	555618	NK-1
175Lu	C1QC	abcam	ab247391	EPR2984Y
176Yb	Pan-cytokeratin	biolegend	914204	AE-1/AE-3
194Pt	αSMA	biolegend	904601	1A4
198PT	Vimentin	CST	46173SF	D21H3
191/193Ir	DNA	Standard BioTools	NA	NA

Supplementary Table S4. Macrophage top 20 highly variable genes

Cluster	Gene list
IL1B⁺ Macrophage	CCL4, CCL4L2, CCL2, CCL3, CCL3L1, G0S2, IL1B, CXCL8, CCL20, IER3, C15orf48, CXCL3, ICAM1, PLAUR, TNFAIP3, NFKBIA, DUSP2, CD83, SOD2, SERPINB9
S100A9⁺ Macrophage	S100A8, VCAN, S100A9, TIMP1, S100A4, SLC2A3, FGL2, C1orf162, CFP, NAMPT, FOS, CD55, MNDA, DUSP1, CEBPB, SLC11A1, S100A6, LST1, JAML, IFI30
SPP1⁺ C1QC^{median} RTM	SPP1, APOE, APOC1, GPNMB, FBP1, MMP9, FABP5, CD9, LIPA, LGMN, CSTB, CTSD, FN1, ACP5, CTSB, NUPR1, CAPG, RARRES1, PTGDS, CTSL
C1QC⁺ RTM	C1QC, C1QA, C1QB, MS4A6A, SLCO2B1, SELENOP, FOLR2, F13A1, SLC40A1, MS4A4A, PLTP, GPR34, STAB1, MRC1, MAF, RNASE1, IFI27, MAFB, MS4A7, A2M

Supplementary Table S5. Macrophage-related gene set

Signature	Gene list
RTM	CD163, LYVE1, FOLR2, APOE, C1QC, C1QA, C1QB
M2	GPR86, P2RY5, TGFBR2, HRH1, TLR5, DCL-1, MSR1, CXCR4, DECTIN1, P2RY14, DCSIGN, CLECSF13, MS4A4A, MRC1, IGF1, CCL23, CCL18, CCL13, SLC21A9, SLC4A7, SLC38A6, CTSC,

	HEXB, LIPA, ADK, HNMT, TPST2, CERK, HS3ST2, LTA4H, CA2, ALOX15, HS3ST1, TGFBI, SEPP1, CHN2, FN1, FGL2, GAS7, EGR2, MAF
M1	CCR7, IL2RA, IL15RA, IL7R, CXCL11, CCL19, CXCL10, CXCL9, TNF, CCL5, CCL15, IL12B, IL15RA, TRAIL, IL6, CCL20, PBEF1, ECGF1, BCL2A1, FAS, BIRC3, GADD45G, HSXIAPAF1, SLC7A5, SLC21A15, SLC2A6, SLC31A2, INDO, PLA1A, OASL, CHI3L2, HSD11B1, AK3, SPHK1 ,PFKFB3, PSME2, PFKP, PSMB9, PSMA2, OAS2, PTX3, CSPG2, APOL3, IGFBP4, APOL1, PDGFA, EDN1, APOL2, INHBA, POL6, HESX1, IRF1, ATF3, IRF7

Supplementary Table S6. IPRES geneset

Signature<br>Gene list	MAPKi_INDUCED_EMT<br>ADAM12, AURKA, BCAT1, BGN, CDH11, CENPF, CKS2, COL10A1, COL11A1, COL3A1, COL5A1, COL5A2, COL6A2, COL6A3, DTL, EPYC, FAP, FCGR1B, FN1, GREM1, IGHM, INHBA, KIF2C, LOXL2, LRRC15, MMP11, NCAPG, NID2, NUAK1, RRM2, SLC16A3, SULF1, TNFAIP6, VCAN
Signature<br>Gene list	MAPKi_INDUCED_ANGIOGENESIS<br>ANPEP, BGN, BMP4, CDH5, COL3A1, CYR61, DLL4, EDN1, EMCN, ID1, KDR, NRP1, PLAU, PPAP2B, PROK2, PRRX2, RHOB, ROBO4, SOX17, SOX18, TGFB2, THBS1, THY1, VEGFA, VEGFC
Signature<br>Gene list	EP_BLOOD_VESS_DEVEL_DN_IN_R<br>FGF9, PGF, S100A7, PDGFA, TNFRSF12A, EDN1, ANPEP, PRRX2, SRF, CDH5, TGFB2, SHB, HAND2, HMOX1, ROBO4, RHOB, IL1B, SOX18, SOX17, THBS1, ANGPT2, PPAP2B, CYR61, BMP4, KLF5, FLT1, JUNB, SLIT2, KDR, PROK2, VEGFC, BGN, MEOX2, EREG, ID1, JMJD6, DLL4, VEGFA
Signature<br>Gene list	PH_BLOOD_VESS_DEVEL_DN_IN_R<br>EMCN, ACVRL1, LMO2, IL18, COL3A1, FGF10, ANPEP, ENPEP, PRRX2, GJA4, CXCL12, MMP2, CDH5, TGFB2, EDNRA, ACE, S1PR1, PTK2B, TDGF1, ROBO4, PLCD1, SOX18, SOX17, LOX, PPAP2B, COL18A1, BMP4, SELP, EPAS1, EGFL7, TGFBR2, COL15A1, TBX1, KDR, THY1, PROK2, BGN, ID1, PROK1, DLL4, PLXDC1, NOTCH4, ECSCR, COL1A2, ZFPM2, ATPIF1, ENG
Signature<br>Gene list	EP_RESP_TO_WOUNDING_DN_IN_R<br>F2RL2, S100A8, PDGFB, PDGFA, F2RL1, S100A9, TLR2, CXCR1, CXCR2, IL11, TGFB2, CASP6, FOS, S1PR3, MYD88, LTB4R, HMOX1, SERPINE1, CCL3L3, IL1B, IRAK2, F11, IL18RAP, F10, PLAUR, PROK2, TNFAIP6, THBD, IL20RB, EREG, CARD18, SERPINB2, DSP, RIPK2, KDM6B, NGF, CXCL1, CCL3, CCL2, C9, CXCL3, DRD5, CXCL2, CCL8, BDKRB1, PF4, FPR2, CXCL6, TRIM72, CCL5, SRF, CCL7, TNFRSF1A, PCSK1, MEFV, CCL20, GP1BB, POU2F3, KLKB1, KRT1, CD24, THBS1, PTX3, KLK8, IL6, CEBPB, MAP2K3, S100A12, SOD2, CCL11,

	ITGA5, HBEGF, ID3, SELE, F2R
Signature<br>Gene list	PH_RESP_TO_WOUNDING_DN_IN_R<br>F2RL3, ACVRL1, ADORA3, MASP1, TACR1, TGFB3, CXCR1, FGF10, CXCR2, MMRN1, TGFB2, CFHR1, CFP, CASP6, GP5, DYSF, AOAH, CCL3L3, CFH, MS4A2, LOX, LBP, CFD, XCR1, IL18RAP, PTGER3, CCL4L2, SERPING1, CDO1, MECOM, PROK2, SIGLEC1, CCR7, KLRG1, CD36, THBD, CD40LG, SERPINF2, PLA2G7, TFPI, AOC3, CYSLTR1, C6, COL3A1, C1R, PF4, GPR68, C1S, CCL5, IL23A, CCL23, MEFV, GP1BB, CNR2, NFATC4, NOX4, SELP, KL, EFEMP2, TGFBR2, IGF2, C4BPA, CCL16, CCL18, NOTCH3, VWF, ID3, ENG, SELE, IGFBP4, BMP6
Signature<br>Gene list	MS_RESP_TO_WOUNDING_UP_IN_MAPKi_aPDL1_NR<br>ADORA3, PDGFA, TLR2, ADORA1, TGFB1, IL10, CD97, S1PR3, GPX1, NLRC4, MYD88, CCL3L1, SERPINA3, CHST2, STXBP1, CCL4L2, SERPING1, CD40, GAL, PLAUR, C8G, ADM, CTSB, VSIG4, PLA2G2D, TPST1, GGCX, TF, CCL2, RTN4RL1, ADORA2A, CCL8, C1R, ITGB3, FPR2, TIMP3, TNFRSF4, CCL7, TNFRSF1A, SLC11A1, TNFRSF1B, GP1BB, IL10RB, RAC1, SCN9A, ENO3, C2, SCG2, FN1, SPP1, NOX4, PLAT, IL2RA, TNFSF4, STAT3, CCNB1, APOL2, CD55, TFRC, C1RL, SYT17, TF, CCL2, FLT1, ACTN4, SOCS3, PDGFA, ALDOC, EGLN3, NR4A2, TGFB1, DDIT4, CD38, HYOU1, HSP90B1, PLOD1, TFRC, ADM, PLOD2, VEGFA, PSEN2, MT3, ANGPTL4
Signature<br>Gene list	POST_OP_WOUNDHEALING<br>MMP3, PPBP, CXCL5, PTX3, PTHLH, TDO2, SPINK6, SPP1, MMP10, IL8, MME, GREM1, CTSZ, CXCL6, THBS1, SCG5, TFPI2, PTGS2, CXCL1, IL1A, PCSK1, AREG, IL13RA2, KIAA1199, CCL18, FST, LILRB1, CTNNB1, CLC, CXCL3, CEACAM6, LILRB2, ITCH, S100A12, CCDC102B, GLIS3, MS4A6E, RARRES1, NRG1, PHLDA1, MS4A4A, HAS2, TFEC, CCR1, ANXA3, CR1, IL1RL1, ADAM12, CCNA1, PLA2G7, ENPEP, SPON1, INHBA, STEAP1, STEAP4, TMSB15A, FGF7, PI15, C8orf4, CYBB, MED18, IGSF6, SAA1, RGS13, DEFB4A, SLC16A3, CCL3, AQPEP, CYP1B1, FAM20A, DKK 1.00, IKBIP, SULF1, PXDN, HMOX1, FMO3, SERPINA3, NAA15, MSR1, CCL8, TMEFF1, KLK6, C13orf33, TNFAIP6, MGST1, SRSF6, SRGN, IGF2BP3, PCSK5, LAMC2, OLFML2B, NCEH1, FABP4, IL6, C5AR1, ALDH1A3, PDPN<br>LYZ, CD163, RAB12, RGS18, HBB, TIMP1, CNN3, FAM83A, CYR61, TNC, DPYSL3, PRR16, BAG2, DSEL, LIPG, PLAC8, CXCL2, FCER1G, SUSD5, NEXN, KLHL6, LMNB1, GPRC5A, TCEAL7, FPR1, APOBEC3A, ITGB6, HS3ST1, GBP6, ITGB5, ADIPOQ, CPXM1, PKP2, NNMT, OLR1, PPP3R1, BUB1, BCL2A1, MAP9, GCLM, S100P, F3, TMPRSS11E, BEND6, FCGR3A, DDX3Y, PI3, MS4A7, FCN1, TLR4, UCHL1, CYTL1, ST8SIA4, MMP9, ALDH1L2, DEPDC1, RNASE2, SPINK7

Signature<br>Gene list	HOEK_INVASIVE_SIG<br>ADAM12, AMOTL2, AXL, BIRC3, CDH13, CDK14, COL13A1, CRIM1, CRISPLD2, CYR61, DPYD, EFEMP1, EGFR, F2RL1, FGF2, FLNB, FOXD1, FST, FZD2, HEG1, HS3ST3A1, ITGA2, ITGA3, KCNMA1, LOXL2, MYOF, NRP1, NTM, NUAK1, OSMR, PDGFC, PODXL, S100A2, SLC22A4, SLIT2, SYNJ2, TCF4, THBS1, TLE4, TNFRSF11B, TPBG, TPM1, TRAM2, WNT5A, ZEB1
Signature<br>Gene list	HOEK_PROLIFEATIVE_SIG<br>ACP5, ADCY2, APOE, ASAH1, BIRC7, C21orf91, CAPN3, CDH1, CDK2, CDK5R1, CEACAM1, DAPK1, DCT, FAM174B, GALNT3, GNPTAB, GPM6B, GPR143, GPRC5B, GYG2, HPS4, INPP4B, IRF4, IVNS1ABP, KAZ, MBP, MICAL1, MITF, MLANA, MYO1D, NR4A3, OCA2, PHACTR1, PIR, PLXNC1, PMEL, RAB27A, RAB38, RGS20, RHOQ, RRAGD, SEMA6A, SIRPA, SLC45A2, ST3GAL6, STX7, TNFRSF14, TRPM1, TYR, TYRP1, WDR91, ZFYVE16
Signature<br>Gene list	MAPKR_BLOOD_VESS_DEVEL_UP<br>CAV1, NRP1, EPAS1, COL3A1, EDN1, COL5A1, CITED2, CDH13, VEGFC, S1PR1, JUN, CCBE1, PLCD3, FOXC2, COL1A1, FGF2, PLAU, CYR61
Signature<br>Gene list	DTPP_BLOOD_VESS_DEVEL_UP<br>CAV1, NRP1, LMO2, EDN1, COL3A1, MMP2, CXCL12, CITED2, AGT, CCBE1, PLCD3, RHOB, SEMA3C, THBS1, FGF2, CEACAM1, SCG2, CYR61, BMP4, COL18A1, EPAS1, MMP19, MYH9, ARHGAP24, COL5A1, THY1, CDH13, VEGFC, BGN, EPGN, JUN, VEGFA, NTRK2, COL1A2, COL1A1, PLAU
Signature<br>Gene list	DTP_BLOOD_VESS_DEVEL_UP<br>CAV1, NRP1, LMO2, EDN1, COL3A1, TNFSF12, MMP2, CITED2, ANGPTL6, CXCR4, PLCD3, RHOB, QKI, SEMA3C, THBS1, CYR61, KLF5, COL18A1, EPAS1, MMP19, MYH9, ARHGAP24, COL5A1, ANXA2, THY1, SMO, CDH13, BGN, JUN, NTRK2, COL1A2, COL1A1, PLAU
Signature<br>Gene list	MAPKR_REG_CELL_PROLIF_UP<br>CAV2, RBP4, CAV1, FOSL2, CCL2, NRP1, IGFBP7, CLU, EDN1, NFKBIA, IL15, IL34, SOX9, S1PR3, AGTR1, BDNF S1PR1, SPEG, HLX, SERPINE1, NKX3-1, PDGFC, CD24, NRG1, RUNX2, FGF2, EGFR, PTGER2, TP53I11, IL6, IRS1, PDCD1LG2, MXD4, VEGFC, CDH13, TNFRSF9, ADRB2, JUN, F3, BNC1, IL12A, PDGFRB, TGFB1I1, PLAU, NGF
Signature<br>Gene list	DTPP_REG_CELL_PROLIF_UP<br>RARRES3, FOSL2, FGF7, NRP1, PDGFB, FGF17, IGFBP7, EDN1, GJA1, FOXO4, CXADR, VIPR1, GLI3, IL31RA, AZGP1, AGTR1, WISP2, BDNF, GPC3, HLX, SERPINE1, PDGFC, NRG1, FGF2, EBI3, EGFR, PRKCA, PTPRK, PTGER2, CD40, IRS1, PDCD1LG2, MXD4, MYCN, TNS3, VEGFC, ADRB2, ADAMTS8, CCND2, CHRM1, F3, JUN, BTG4, GRN, VEGFA, IL12A, PDGFRB, NGFR, TGFB1I1, PMP22, NGF, CAV2, CAV1, CCL2, IFITM1, CLU, PTH1R,

	NFKBIA, KIT, BDKRB2, IL34, TIMP2, SOX9, ADA, VDR, IL12RB1, SPEG, AGT, ADRA2A, NKX3-1, CD24, THBS1, PPAP2A, RUNX2, SCG2, BMP4, COL18A1, IL6, TP53I11, TNFSF4, KAT2B, HCLS1, KLF11, TAX1BP3, CDH13, ATF3, NUPR1, EPGN, ETS1, DLX5, BNC1, FABP4, NR5A2, PLAU, KCTD11, F2R
Signature	DTPP_CELL_ADHESION_UP
Gene list	CADM3, NRP1, THRA, CADM1, TLN2, IGFBP7, NPNT, FERMT2, BCAM, L1CAM, EDIL3, CXADR, CXCL12, VCL, NRCAM, AZGP1, WISP2, TGFBI, RHOB, LOXL2, NEGR1, BOC, CEACAM1, CDH24, CYR61, SPON1, EGFR, F11R, PTPRK, PCDHB7, NRXN2, CNTN6, SDK1, CPXM2, MYH9, THY1, JUP, CD36, CLDN1, LAMC2, TGFB1I1, PARVA, ACHE, CCL2, COL3A1, ITGA11, COL28A1, SPOCK1, IL32, CDH3, PCDHB11, SOX9, APLP1, ALCAM, COL17A1, LAMB2, SORBS1, FAT4, AGT, TTYH1, COL6A3, MSLN, CD24, THBS1, SELPLG, THBS3, APBA1, NPHP1, FN1, COL18A1, FLRT1, BGLAP, BMP1, COL13A1, ITGA1, NFASC, HSPG2, CELSR2, NID2, PCDH17, COL5A3, COL16A1, COL5A1, COL4A6, CDH13, ERBB2IP, ITGA5, PKP3, ADAM22, NTM, FEZ1
Signature	PLX2D_CELL_ADHESION_UP
Gene list	THRA, TLN2, FERMT2, L1CAM, BCAM, EDIL3, VCL, AZGP1, WISP2, RHOB, LOXL2, COL11A1, CYR61, PCDHB5, CNTN6, MYH9, JUP, NCAM2, CD36, CD99L2, TGFB1I1, PARVA, COL3A1, PCDHB15, NINJ1, PCDHB11, PKD1L1, CLDN14, ALCAM, SORBS1, ROPN1B, TTYH1, PVRL2, MSLN, ACAN, CD24, THBS1, GPNMB, APBA1, THBS3, COL18A1, MAG, FLRT1, ADAM23, ITGA1, HSPG2, CELSR3, NID2, PCDH17, COL16A1, COL5A3, PCDH18, COL14A1, FREM2, CDH19, CYFIP2, ANTXR1, ABL2
Signature	DTPP_RESP_TO_WOUNDING_UP
Gene list	F2RL2, NRP1, FGF7, PDGFB, F2RL1, TLR3, DYSF, SERPINE1, CFH, NRG1, FGF2, IRAK2, F11R, LY96, CD40, SDC1, CD36, F3, TFPI, NGFR, NGF, ACHE, CCL2, RTN4RL1, C3, CXCL3, COL3A1, CXCL2, CLU, C1R, BDKRB2, C1S, CDH3, MDK, TPM1, CCL26, IGSF10, LAMB2, NFATC4, CD24, THBS1, FN1, SCG2, IL6, TNFSF4, EFEMP2, MSTN, COL5A1, APOL3, PLSCR4, NUPR1, ITGA5, CD59, AOX1, PLA2G4C, HDAC9, PLAU, IGFBP4, F2R

Supplementary Table S7. MHC gene set

MHC-I	B2M, HLA-A, HLA-B, HLA-C, NLRC5, TAP1, TAP2, TAPBP
MHC-II	CIITA, HLA-DMA, HLA-DMB, HLA-DPA1, HLA-DPB1, HLA-DQA1, HLA-DQB1, HLA-DRA, HLA-DRB1

Supplementary Table S8. Gene sets of CD4T, Fibroblast, and APC

Signature	Gene list
CD4T	TRAC, IL7R, KLRB1, TRBC1, LTB, IL32, TRBC2, TNFRSF4, CD2, SPOCK2, CD3D, TNFRSF18, RGS1, CD3G, BATF, MAF, CXCL13
Fibroblast	COL1A1, COL3A1, COL1A2, LUM, DCN, TAGLN, RARRES2, TIMP1, ACTA2, COL6A2, MGP, MMP2, COL6A3, CALD1, SPARC, FBLN1, MYL9
APC	IFNG, TNF, PSME1, PSME2, PSME3, HSPA8, HSPA1A, HSPA1L, HSPA1B, HSPA6, HSPA2, HSPA4, HSP90AA1, HSP90AB1, HLA-A, HLA-B, HLA-C, HLA-F, HLA-G, HLA-E, HSPA5, CANX, B2M, PDIA3, CALR, TAPBP, TAP1, TAP2, CD8A, CD8B, CD8B2, KIR3DL2, KIR3DL1, KIR3DL3, KIR2DL2, KIR2DL1, KIR2DL3, KIR2DL4, KIR2DL5A, KLRC1, KLRC2, KLRC3, KLRC4, KLRD1, KIR2DS1, KIR2DS3, KIR2DS4, KIR2DS5, KIR2DS2, IFI30, LGMN, CTSB, HLA-DMA, HLA-DMB, HLA-DOA, HLA-DOB, HLA-DPA1, HLA-DPB1, HLA-DQA1, HLA-DQA2, HLA-DQB1, HLA-DRA, HLA-DRB1, HLA-DRB3, HLA-DRB4, HLA-DRB5, CD74, CTSL, CTSS, CD4, CIITA, RFX5, RFXANK, RFXAP, CREB1, NFYA, NFYB, NFYC

Supplementary Table S9. Immunotherapy cohort

Dataset	Cancer	Patient	Treatment
GSE115821	SKCM	41	aPD-1, aCTLA-4
GSE135222	NSCLC	45	aPD-1, aPD-L1
GSE136961	NSCLC	21	aPD-1
GSE145996	SKCM	52	aPD-1
GSE165252	ESCA	40	aPD-L1
GSE173839	BRCA	105	aPD-L1
GSE176307	UC	103	aPD-1, aPD-L1
GSE35640	SKCM	65	MAGE-A3
GSE67501	RCC	11	aPD-1, aPD-L1
GSE91061	SKCM	65	aPD-1, aCTLA-4
GSE93157	NSCLC	65	aPD-1
PRJEB23709	SKCM	158	aPD-1
PRJEB25780	STAD	45	aPD-1
PRJNA482620	GBM	17	aPD-1

Gide et al.	SKCM	73	aPD-1, aPD1+aCTLA-4
Liu et al.	SKCM	121	aPD-1
Snyder et al.	UC	25	aPD-L1
Van et al.	SKCM	36	aCTLA-4
